# Copper pyrithione complexes with endoplasmic reticulum localisation showing anticancer activity *via* ROS generation

**DOI:** 10.1039/d4sc06628f

**Published:** 2025-10-08

**Authors:** Atreyee Mishra, Dominic J. Black, Thomas S. Bradford, Karrera Y. Djoko, Benjamin J. Hofmann, Jamie J. Hunter, Rianne M. Lord, Robert Pal, Harvey J. Smart, Tameryn Stringer, James W. Walton

**Affiliations:** a Durham University, Department of Chemistry Durham DH1 3LE UK james.walton@durham.ac.uk; b Durham University, Department of Biosciences Durham DH1 3LE UK; c School of Chemistry, University of East Anglia Norwich NR4 7TJ UK; d Department of Chemistry, University of Warwick Coventry CV4 7SH UK; e School of Science, The University of Waikato Hamilton 3210 New Zealand

## Abstract

Copper complexes have great potential to overcome the disadvantages of platinum-based anticancer agents, owing to their lower systemic toxicity. Copper pyrithione showed early promise as an anticancer candidate, but further studies have not been forthcoming. Herein, we report a series of copper pyrithione derivatives that show between 1 and 2 orders of magnitude higher activity than cisplatin against pancreatic and breast cancer cell lines, along with good selectivity over healthy cells. Reactive oxygen species (ROS) generation is determined to be a likely mode of action. A fluorescent analogue shows localisation in the endoplasmic reticulum of cells, which is highly unusual for metal-based therapeutics and opens up the potential for unique modes of therapeutic action.

## Abstract 2

A series of copper pyrithione derivatives show sub-micromolar IC_50_ activity against cancer cell lines, good selectivity over non-cancerous cells, reactive oxygen species generation and endoplasmic reticulum localisation.

## Graphical abstract

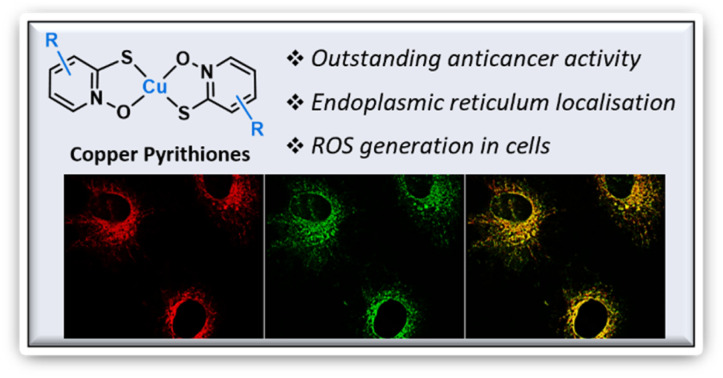

## Introduction

Cisplatin and related platinum complexes are used in many cancer treatments, with particular benefit in lung and bladder cancers.^1,2^ The negative side-effects of these complexes include nephrotoxicity, nausea and ototoxicity, and have led to the search for alternative metal-based therapeutics.^3,4^ Many examples of Pt,^5,6^ Ru,^7–12^ Ir^13–15^ and Rh^16–18^ complexes have undergone initial studies. However, transition to the clinic has been limited owing, in part, to the high cost of synthesis, low earth abundance and potential systemic toxicity of these platinum group metals. The development of metal-based complexes containing earth-abundant metals with low inherent systemic toxicity offers a better approach to drug design.

**Fig. 1:**
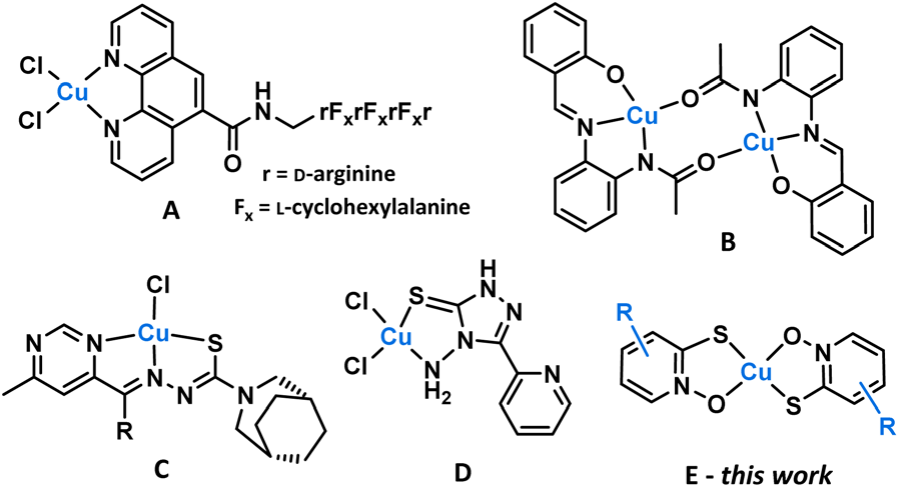
Copper complexes with anticancer activity, including examples of (A) polypyridyl,^21^ (B) *N*,*N*,*O*-chelator,^23^ (C) thiosemicarbazone^26^ and (D) disulfiram^27^ ligands. (E) [Cu(PT)_2_] derivatives featured in this work.

Copper is an essential element, required for several key processes in the human body.^19^ Copper salts (*e.g.*, CuCl_2_) show low cytotoxicity in cell assays, but coordinating ligands can be designed to impart cytotoxicity, leading to potential anticancer agents.^20^ Copper complexes incorporating polypyridyl,^21,22^*N*,*N*,*O* chelator,^23^ thiosemicarbazone,^24–26^ and disulfiram^27–29^ ligands have been developed for this application (Fig. 1).

Metal-based anticancer therapeutics typically target nuclear DNA (*e.g.*, cisplatin) or enzymes in the cytoplasm (*e.g.*, Au(i) complexes targeting thioredoxin reductase^30^). However, targeting other organelles could lead to improved and/or complementary treatments. The endoplasmic reticulum (ER) plays crucial roles in the production of proteins, lipids and hormones. Metal-based complexes that target the ER are rare, but targeting this novel site of action may lead to more effective therapeutics that overcome the limitations of current compounds.

Copper pyrithione complexes represent a promising new class of copper-based therapeutics.^31,32^ The parent complex [Cu(PT)_2_] (PT = pyrithione) was found to have cytotoxicity, with submicromolar IC_50_ values against MCF-7 (breast), HepG2 (liver), U266 and NCI-H929 (myeloma) cancer cell lines.^33^ Activity was linked to the inhibition of deubiquitinase, leading to disruption of the proteasome. Recently, we reported a series of highly water-soluble polyethylene glycol derivatives of copper pyrithione, which retained high therapeutic activity.^34^ Despite the promise of [Cu(PT)_2_] as a potential anticancer agent, there have been no further studies exploring pyrithione derivatives for this application. Herein, we investigate a series of substituted copper pyrithione complexes (Fig. 1E) that look to understand the link between structure and activity. Outstanding therapeutic activity is observed, along with cell localisation in the endoplasmic reticulum, which is highly unusual and indicates a potential novel mode of action.

## Results and discussion

We set out to determine the localisation of copper pyrithione complexes within the cell and establish whether substituents on the PT pyridyl ring improve therapeutic activity and physical properties (lipophilicity and stability). Substituents include electron-donating groups, electron-accepting groups, aromatics and a fluorescent tag, to allow us to track cell localisation and the relationship between structure and activity.

### Synthetic procedures and photophysical characterisation

**Fig. 2:**
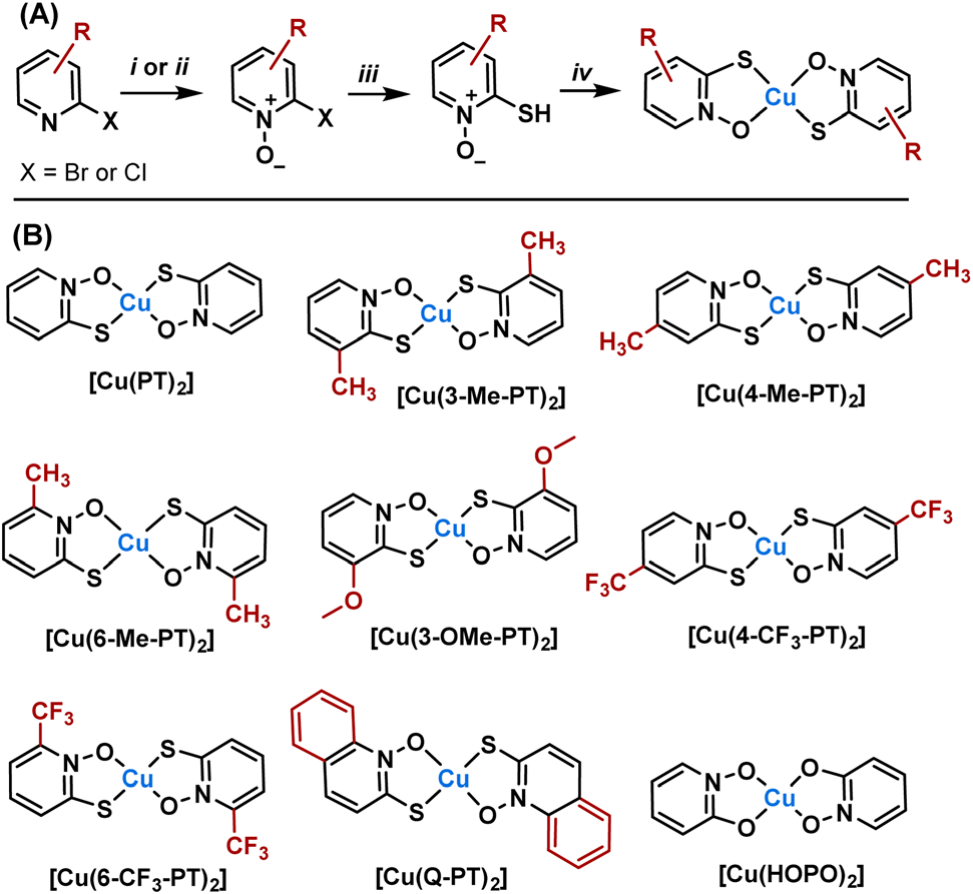
(A) Synthesis of copper pyrithione complexes. (i) *m*CPBA, CH_2_Cl_2_, 25 °C, 16 h. (ii) Urea hydrogen peroxide, trifluoroacetic anhydride, CH_2_Cl_2_, 25 °C, 16 h. (iii) NaSH·H_2_O, H_2_O, 25 °C, 2 h, (iv) CuCl_2_·2H_2_O, H_2_O and (B) list of complexes synthesised.

A synthetic route was designed starting from 2-halopyridines, which are commercially available (Fig. 2A). Oxidation to the *N*-oxide was achieved using either *meta*-chloro perbenzoic acid or urea hydrogen peroxide and trifluoroacetic anhydride. The latter was preferred as purification avoided chromatographic separation. Next, substitution of the halide for a thiol was achieved using sodium hydrogen sulfide. Finally, reaction with copper chloride under basic conditions led to complexation. Copper complexes were purified by preparative HPLC and characterised by mass spectrometry, elemental analysis, analytical HPLC and, where possible, single crystal X-ray diffraction. In total, seven new complexes were produced (Fig. 2B), with substituents ranging from electron-donating (Me, OMe) to electron-withdrawing (CF_3_) and aromatic groups. Regioisomers were also produced to determine how isomerism affects activity. For comparison, the parent [Cu(PT)_2_] and the analogous complex [Cu(HOPO)_2_] were also synthesised. Full synthetic and characterisation data are available in the SI.

**Scheme 1:**
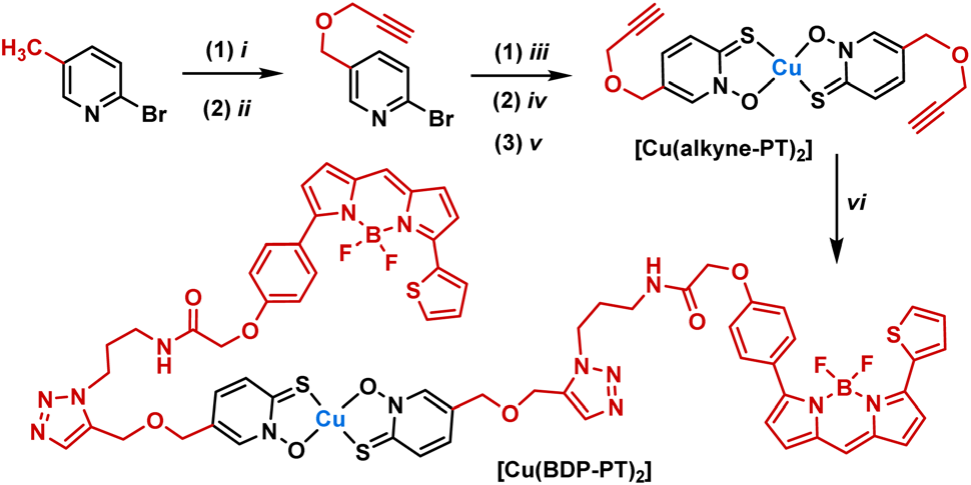
Synthesis of fluorescent copper pyrithione complex [Cu-(BDP-PT)_2_]. (i) *N*-Bromosuccinimide, benzoyl peroxide, CHCl_3_, 62 °C, 16 h, (ii) propargyl alcohol, NaH, THF, 25 °C, 16 h (iii) *m*CPBA, CH_2_Cl_2_, 25 °C, 16 h. (iv) NaSH·H_2_O, H_2_O, 25 °C, 2 h, (v) CuCl_2_·2H_2_O, H_2_O, (vi) BDP-TR-azide, CuSO_4_, (+)-sodium-l-ascorbate, THF : H_2_O : CH_2_Cl_2_ (5 : 2.5 : 1), 55 °C, 3 h.

To understand localisation within the cells, a fluorescent complex was also synthesised (Scheme 1). Initially, an alkyne derivative of copper pyrithione, [Cu(alkyne-PT)_2_], was generated (Scheme 1). Subsequently, a copper catalysed azide–alkyne cycloaddition was carried out with a BODIPY azide, BDP-TR. The resultant complex, [Cu(BDP-PT)_2_] was purified by preparative HPLC and characterised by HRMS and analytical HPLC. Initial photophysical characterisation of [Cu(BDP-PT)_2_] was carried out in MeCN. The complex has an *λ*_max_(absorption) = 587 nm (*ε* = 76 800 M^−1^ cm^−1^), *λ*_max_(emission) = 619 nm and a photoluminescent quantum yield of 43% (full details in SI). These properties are similar to those of free BOPIDY, although the quantum yield is lower, potentially due to quenching from the copper centre. Overall, the data suggest that the complex is suitable for cellular microscopy studies.

### Structural characterisation

**Fig. 3:**
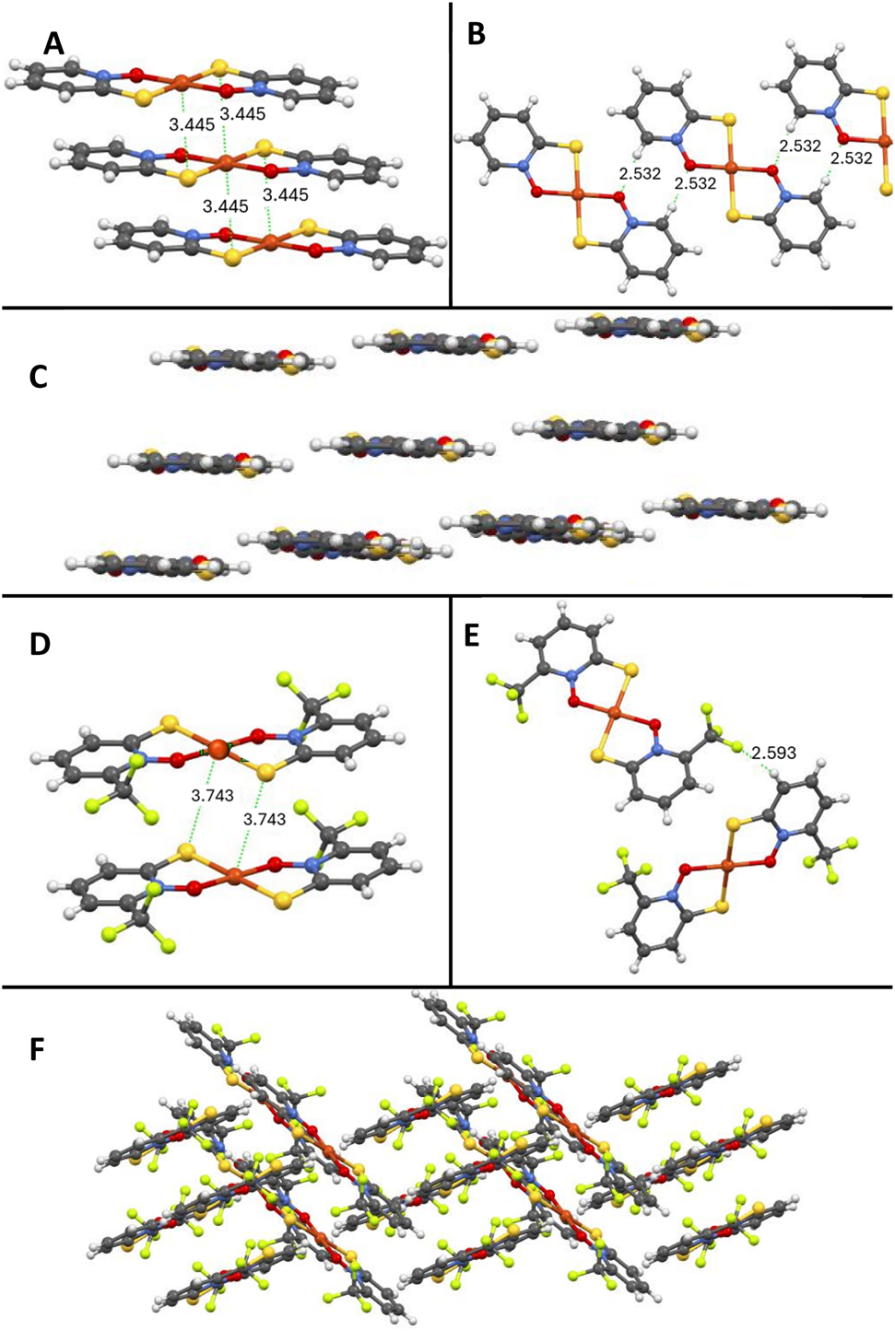
Single crystal X-ray structure of copper pyrithione complexes with key distances highlighted.^35^ [Cu(PT)_2_] intermolecular interactions (A and B) and extended structure (C). [Cu(6-CF_3_-PT)_2_] intermolecular interactions (D and E) and extended structure (F).

Single crystals suitable for X-ray studies of five novel copper pyrithione complexes ([Cu(3-Me-PT)_2_], [Cu(4-Me-PT)_2_], [Cu(6-Me-PT)_2_], [Cu(3-OMe-PT)_2_] and [Cu(6-CF_3_-PT)_2_]) were grown (see SI for method). As an example, the X-ray structure of [Cu(6-CF_3_-PT)_2_] is shown (Fig. 3D–F), while data for the other complexes are provided in the SI. The Cu(ii) centre in [Cu(6-CF_3_-PT)_2_] is square planar in a *trans* configuration. Intermolecular interactions include a F–H hydrogen bond (2.593 Å, Fig. 3E) between adjacent molecules and an interplanar Cu–S interaction (3.743 Å, Fig. 3D). The extended structure shows a herringbone arrangement (Fig. 3F). This structure has several differences compared to the parent complex, [Cu(PT)_2_] (Fig. 3A–C), whose single crystal X-ray structure was previously determined.^35^ For example, [Cu(PT)_2_] has a layered, rather than herringbone, structure and a shorter distance between planes (Fig. 3A, Cu–S interaction at 3.445 Å). These interactions lead to a compact, layered, extended structure (Fig. 3C), which likely contributes to the low solubility of [Cu(PT)_2_]. By observation, [Cu(6-CF_3_-PT)_2_] and the other pyrithione derivatives have greater aqueous and organic solubility than [Cu(PT)_2_], likely due to disruption of the layered packing.

Next, we looked to determine whether the bulk samples were predominantly *cis* or *trans* isomers about the Cu centre. HPLC methods were unsuccessful in determining *cis*/*trans* ratio of complexes, presumably due to similar retention times. We turned to computational modelling to estimate the *cis*/*trans* ratio in the bulk. The results (SI Table S2) show that for all copper pyrithione complexes, the energy difference between *cis* and *trans* isomers is small (between 1.31 and 4.2 kJ mol^−1^), resulting in bulk samples with predicted mixtures of *cis* and *trans* isomers. To understand whether this prediction matches the real data, we measured the FTIR spectrum of [Cu(PT)_2_] (Fig. S21) and compared it to simulated IR spectra of pure *cis* and pure *trans* isomers (Fig. S19 and S20). The region 1000–1500 cm^−1^ corresponds to the N–O asymmetric stretch and the S–O asymmetric stretch. Isomerically pure samples are predicted to show two peaks in this region, whereas the measured sample of [Cu(PT)_2_] showed fours peaks. This data implies that the bulk sample contains a mixture of *cis* and *trans* isomers (see SI for more details).

### Lipophilicity and thermodynamic stability studies

Lipophilicity is an important consideration when designing potential anticancer therapeutics, as it can impact uptake of the compound into the cell.^36^ An octanol : water shake-flask method was used to determine the lipophilicity of a selection of the copper pyrithione complexes (Table 1). Due to the low aqueous solubility of [Cu(PT)_2_], inductively coupled plasma-optical emission spectroscopy (ICP-OES) was used to quantify copper in the octanol and water layers (see SI). The parent complex [Cu(PT)_2_] gave the highest lipophilicity, with a log *P* value of 2.34. Substitution of the pyridyl ring universally lowers the lipophilicity of the complexes (Table 1) and increases observed solubility in aqueous and non-aqueous solvents. Regioisomerism also affects lipophilicity, with [Cu(3-Me-PT)_2_] and [Cu(6-Me-PT)_2_] showing log *P* values of 1.73 and 2.12, respectively. [Cu(6-CF_3_-PT)_2_] has the lowest log *P* value of all those tested, likely due to the increased polarity of the C–F bonds compared to C–H bonds.^37^

**Table 1:** Lipophilicity (log *P*) and stability (log *K*) data for selected copper pyrithione complexes. Full experimental details can be found in the SI

Complex	Log *P*	Log(*K*/(M^−2^))
[Cu(PT)_2_]	2.34	7.528 ± 0.004
[Cu(3-Me-PT)_2_]	1.73	Not measured
[Cu(6-Me-PT)_2_]	2.12	7.498 ± 0.005
[Cu(3-OMe-PT)_2_]	1.75	7.560 ± 0.002
[Cu(6-CF_3_-PT)_2_]	1.45	7.619 ± 0.008

The thermodynamic stability of metal complexes can influence their activity, particularly if ligand exchange is involved in their therapeutic mode of action. We measured the stability constants of [Cu(PT)_2_], [Cu(6-Me-PT)_2_], [Cu(3-OMe-PT)_2_] and [Cu(6-CF_3_-PT)_2_] using the Benesi–Hildebrand method^38^ (Table 1, see SI for details). These complexes were chosen specifically to assess how the electron-donating or withdrawing properties of the substituents influence the apparent stability. In fact, very little variation was observed, with all measured log *K* values falling between 7.498 and 7.619, which implies that binding is dominated by the direct coordination sphere around the copper centre, with strong electrostatic interactions between the charged pyrithione donor atoms and Cu(ii), further enhanced by the chelate effect.

### Therapeutic evaluation

The potential therapeutic activity of the copper pyrithione complexes was determined by screening complexes for 24 hours against human cancer cell lines MCF-7 (breast) and MIA PaCa-2 (pancreatic), alongside a non-cancerous epithelial cell line ARPE-19 (retinal). As controls, cisplatin and CuCl_2_·2H_2_O were also included in the screen (Table 2). Against the MCF-7 cell line, all copper pyrithione complexes were more active than CuCl_2_, highlighting the importance of the ligands around the copper centre. More importantly, all copper pyrithione complexes were more active than cisplatin, in some cases up to 100-fold more, highlighting the therapeutic potential for this class of complexes. Improved activity over cisplatin could lead to drugs requiring lower dosages and, therefore, fewer side effects. Along with [Cu(PT)_2_], the most active species are the methyl- and methoxy-substituted complexes, all showing IC_50_ values in the range 0.2–0.7 μM, implying electron-donating groups on the pyridyl ring improve activity. Indeed, the methoxy-substituted complex shows the highest activity of all tested. Both complexes incorporating trifluoromethyl groups show around 10-fold lower activity than the other pyrithione complexes, which correlates with the lower lipophilicity observed for [Cu(6-CF_3_-PT)_2_].

**Table 2:** IC_50_ values (μM) ± SD for all copper pyrithione complexes, CuCl_2_·2H_2_O and cisplatin against breast cancer MCF-7, pancreatic cancer MIA PaCa-2 and non-cancerous retinal epithelial ARPE-19 cells (24 h incubation, *n* = 9)

Compounds	MCF-7	MIA PaCa-2	ARPE-19
[Cu(PT)_2_]	0.27 ± 0.01	2.22 ± 0.08	0.41 ± 0.05
[Cu(3-Me-PT)_2_]	0.347 ± 0.006	7.1 ± 0.2	2.2 ± 0.1
[Cu(4-Me-PT)_2_]	0.69 ± 0.02	8.5 ± 0.3	1.8 ± 0.1
[Cu(6-Me-PT)_2_]	0.57 ± 0.01	2.1 ± 0.1	0.57 ± 0.03
[Cu(3-OMe-PT)_2_]	0.22 ± 0.01	2.7 ± 0.1	0.87 ± 0.04
[Cu(4-CF_3_-PT)_2_]	8.9 ± 0.3	21.6 ± 0.4	17.3 ± 0.4
[Cu(6-CF_3_-PT)_2_]	4.72 ± 0.03	9.8 ± 0.3	8.9 ± 0.2
[Cu(Q-PT)_2_]	4.6 ± 0.1	17.3 ± 0.2	10.4 ± 0.4
[Cu(HOPO)_2_]	35.9 ± 0.2	70 ± 2	45 ± 2
CuCl_2_·2H_2_O	>100	>100	83 ± 2
Cisplatin	40 ± 1	>100	13.0 ± 0.1

Against the MIA PaCa-2 cell line, pyrithione complexes are more active than CuCl_2_, [Cu(HOPO)_2_] or cisplatin. Compared to MCF-7 cells, broadly similar trends in pyrithione derivatives are seen, with slightly lower overall activity (Table 2). The parent [Cu(PT)_2_], methoxy-derivative and 6-methyl complexes are again the most active, showing excellent cytotoxicity.

**Fig. 4:**
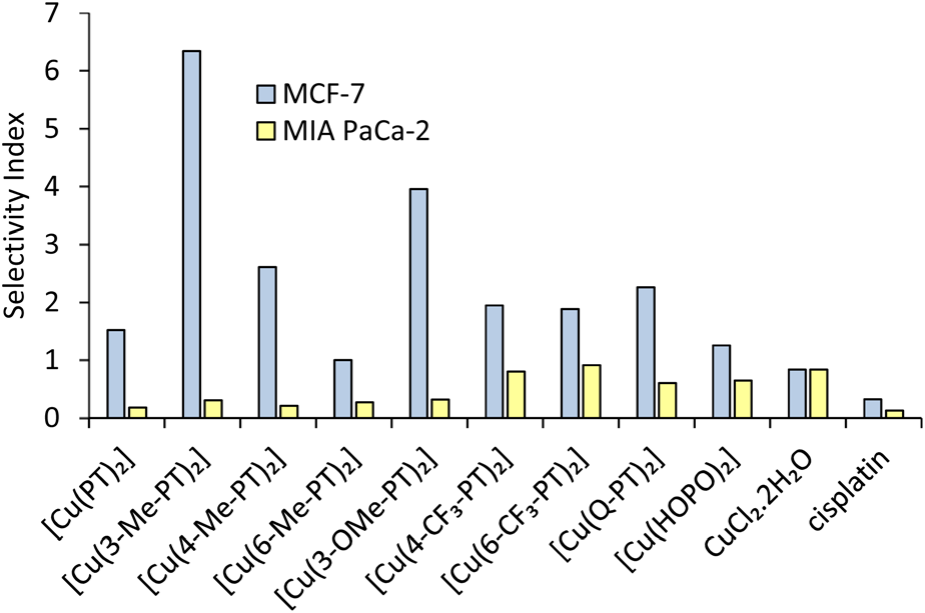
Selectivity index, defined as the ratio of IC_50_ values against healthy and cancerous cells, for all copper pyrithione complexes, CuCl_2_·2H_2_O and cisplatin against breast cancer MCF-7 (blue bars) and pancreatic cancer MIA PaCa-2 cells (yellow bars).

A selectivity index (SI) can be defined as the IC_50_ value against non-cancerous cells divided by the IC_50_ against cancerous cells (Fig. 4). Under our conditions, cisplatin shows low selectivity, with SI values of 0.35 and <0.13 against MCF-7 and MIA PaCa-2, respectively. All pyrithione complexes show SI values >1 against the MCF-7 cell line (Fig. 4 blue bars), showing higher activity against cancer cells than non-cancer cells. Generally, substitution of the pyridyl ring leads to increased selectivity, with the 3-methyl and 3-methoxy derivatives showing the highest SI values of 6.34 and 3.95, respectively.

Overall, the cytotoxicity data show that copper pyrithione complexes have excellent potential to act as anticancer agents, especially as they are more active and selective than cisplatin. Furthermore, substitution of the pyridyl ring modulates activity and improves selectivity.

### Cellular localisation studies

**Fig. 5:**
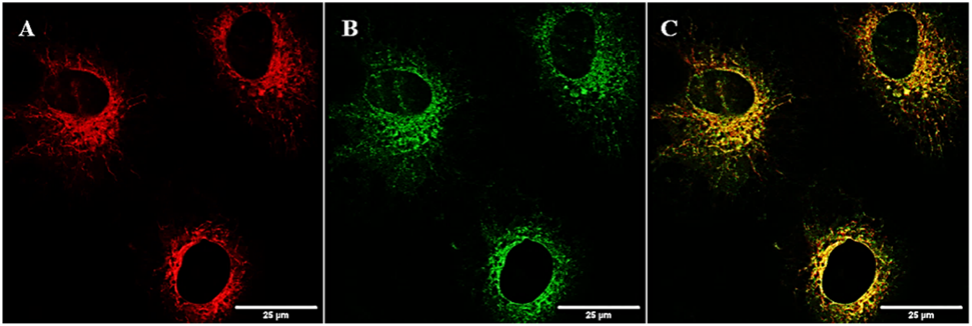
Fluorescence microscopy images with NIH-3T3 cells. (A) Fluorescence from [Cu(BDP-PT)_2_] (1 μM, 2 h incubation, *λ*_ex_ = 543 nm, *λ*_em_ = 600–650 nm) (B) fluorescence from ER-Tracker Green™ (*λ*_ex_ = 496 nm, *λ*_em_ = 510–550 nm) (C) overlayed image.

Knowing the sub-cellular localisation of copper pyrithione complexes can aid in determining potential modes of therapeutic action. To establish sub-cellular localisation of pyrithione complexes, NIH-3T3 fibroblast cells were dosed with the fluorescent complex [Cu(BDP-PT)_2_] at 1 μM concentration and microscopy studies were carried out. After 2 hours incubation, images were collected following excitation at 543 nm and fluorescence measured at 600–650 nm. Fig. 5A shows that the complex enters the cell and remains fluorescent. To understand complex localisation, cells were co-stained with commercial dyes. MitoTracker Green™ showed poor overlap with the fluorescence from [Cu(BDP-PT)_2_] (Fig. S17, Manders' overlap coefficient = 0.40 (ref. 39)), suggesting that the complex does not localise in the mitochondria. LysoTracker Green™ showed similarly poor overlap (Fig. S18, Maunders' coefficient = 0.45). By contrast, ER-Tracker Green™ (Fig. 5B) showed excellent overlap of fluorescence (Fig. 5C, Manders' overlap coefficient = 0.80 (ref. 39)), strongly suggesting that this copper complex localises at the endoplasmic reticulum.

ER targeting of metal-based therapeutics is rare. More common sites of action are the nucleus, mitochondria and lysosomes.^40^ ER stress is associated with apoptosis, and metal-based therapeutics that target the ER have great potential in leading to new anticancer agents. Although limited reports of Ir, Ru and Pt complexes that target the ER have been published,^41^ to the best of our knowledge, only one previous example of a copper complex locating in the ER is known.^42^ The combination of therapeutic activity matching with the best-in-class for copper complexes and targeting an alternative site of action within the cell gives the copper pyrithiones excellent potential as novel and complementary cancer therapies.

### Reactive oxygen species formation

**Fig. 6:**
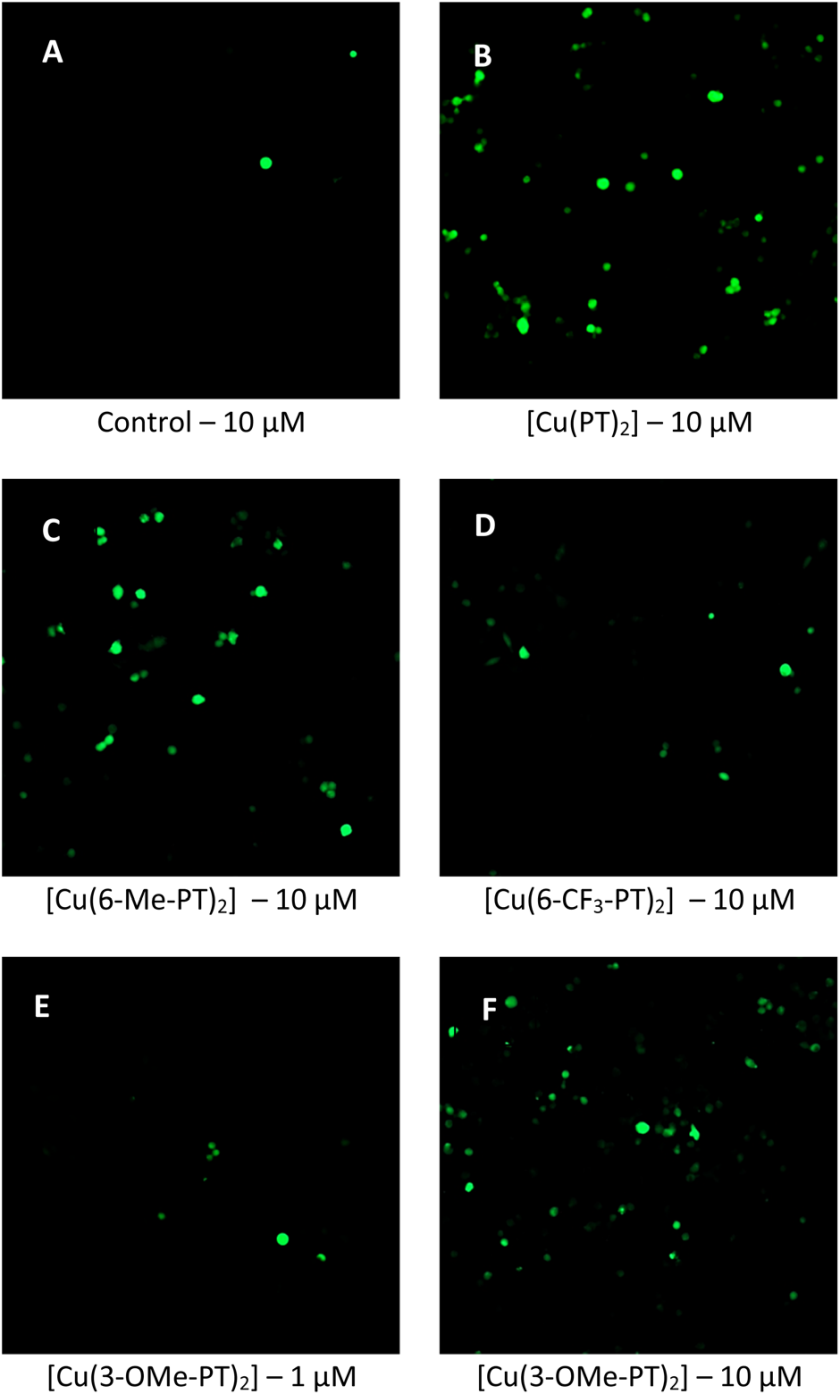
Fluorescence microscopy images of MCF-7 cells after 1 h dosed with (A) no additional complex (untreated control cells), (B) [Cu(PT)_2_] at 10 μM, (C) [Cu(6-Me-PT)_2_] at 10 μM, (D) [Cu(6-CF_3_-PT)_2_] at 10 μM, (E) [Cu(3-OMe-PT)_2_] at 1 μM and (F) [Cu(3-OMe-PT)_2_] at 10 μM. Staining was conducted *via* a 30-minute incubation with H_2_DCFDA, excitation at 488 nm and emission at 509 nm (see SI for full details).

To understand a potential mechanism of action, we determined production of reactive oxygen species (ROS) using fluorescent microscopy assays. ROS production is a common mode of therapeutic activity of copper complexes.^43^ MCF-7 cells were dosed with [Cu(PT)_2_], [Cu(6-Me-PT)_2_], [Cu(6-CF_3_-PT)_2_] and [Cu(3-OMe-PT)_2_] at 10 μM for 1 h and a fluorescent ROS probe 2′,7′-dichlorodihydrofluorescein diacetate (H_2_DCFDA) was added at 20 μM final concentration. In the presence of ROS, H_2_DCFDA converts to a fluorescent dye that can be observed using microscopy. As can be seen in Fig. 6B–F, fluorescence appears in all cells dosed with pyrithione complexes. By contrast, control cells with no pyrithione complex added show little fluorescence (Fig. 6A). Between the four complexes tested, the brightest fluorescence was observed for cells treated with [Cu(PT)_2_] (Fig. 6B), [Cu(6-Me-PT)_2_] (Fig. 6C), and [Cu(3-OMe-PT)_2_] (Fig. 6F), whereas fluorescence from cells treated with [Cu(6-CF_3_-PT)_2_] (Fig. 6D) was weaker. This variation correlates well with the cytotoxicity data in Table 2, with the methyl and methoxy complexes also showing the highest activity. Thus, these results support the hypothesis that ROS production leads to toxicity.

Next, cells were dosed at two concentrations of pyrithione complex to determine whether production of ROS is dose-dependent. Results for [Cu(3-OMe-PT)_2_] are shown at 1 μM (Fig. 6E) and 10 μM (Fig. 6F) after 1 hour; results for other complexes are shown in the SI (Fig. S11 and 12). In all cases, the fluorescence intensity increases in assays with 10 μM complex, compared to assays with 1 μM complex, supporting a mode of action where increasing complex concentration leads to increased ROS generation and higher cytotoxicity.

To provide a comparison with compounds that are known to generate ROS, MCF-7 cells were dosed with either [Cu(PT)_2_] or menadione (a known oxidative stress inducer). Dosed cells were fixed and treated with the CellROX stain. The results show that lower levels of ROS are generated with [Cu(PT)_2_], compared to menadione (Fig. S13). This result implies that, while ROS generation is important, it may not be the only contributing factor to copper pyrithione toxicity.

Finally, an additional assay was carried out to determine whether the observed therapeutic selectivity between cancerous and non-cancerous cells was linked to ROS generation. Non-cancerous ARPE-19 and cancerous MCF-7 cells were dosed with selected copper pyrithione complexes, fixed and imaged using the CellROX green stain. A general trend emerged whereby dosed MCF-7 cells showed consistently higher ROS levels than the dosed ARPE-19 cells (Table S1). While it is known that MCF-7 cells have higher basal levels of ROS than ARPE-19,^44^ the data support the hypothesis that ROS generation is a factor in the observed selectivity.

## Conclusion

In conclusion, we present a novel series of copper pyrithione complexes that show a potential future replacement for cisplatin. The complexes are produced *via* a straightforward, 3-step synthesis from readily available precursors and an earth-abundant metal salt. In assays against two cancer cell lines, the complexes show up to 100-fold higher cytotoxicity than cisplatin, which matches the best-in-class for anticancer copper complexes. Furthermore, we show the ability to tune selectivity towards cancerous over non-cancerous cells through substituents around the pyridine ring. By measuring physical properties, including lipophilicity, we have started to understand the factors that control activity, and through cell mechanistic studies, we have shown that ROS production is a credible mode of therapeutic action of these complexes. Most importantly, we have shown that the endoplasmic reticulum is a likely target for these complexes. The novelty of ER-targeting metal complexes makes the copper pyrithione series of complexes very exciting for further study. Overall, this first study of a copper pyrithione family as anticancer agents augurs well for future development.

## Author contributions

Project design by JWW, KYD and AM. Synthesis and characterisation by AM, JJH and HJS. Cytotoxicity and ROS assays by RML, TS and BJH. Microscopy studies by TSB, DJB and RP. Computational work by HJS. Manuscript written by AM and JWW, with edits from all authors.

## Conflicts of interest

There are no conflicts to declare.

## Supplementary Material

SC-016-D4SC06628F-s001

SC-016-D4SC06628F-s002

## Data Availability

CCDC 2377352–2377357 contain the supplementary crystallographic data for this paper.^45*a*–*f*^ The data supporting this article have been included as part of the supplementary information (SI). Supplementary information: synthetic details, physical measurements, details of biological assays, computational study, X-ray crystallography data and NMR spectra. See DOI: https://doi.org/10.1039/d4sc06628f.
