# Supplementary material for: Copper pyrithione complexes with endoplasmic reticulum localisation showing anticancer activity via ROS generation
Source: Chem Sci. 2025 Oct 8;16(44):21104–10. doi: 10.1039/d4sc06628f (PMC12519996; doi:10.1039/d4sc06628f)
Supplement: SC-016-D4SC06628F-s001 [file SC-016-D4SC06628F-s001.pdf]

Supplementary Information (SI) for Chemical Science.
This journal is © The Royal Society of Chemistry 2025

Copper Pyrithione Complexes with Endoplasmic Reticulum Localisation Showing Anticancer Activity via ROS Generation

Atreyee Mishra,[a] Dominic J. Black,[a] Thomas S. Bradford,[a] Karrera Y. Djoko,[b] Benjamin J. Hofmann,[d] Jamie J. Hunter ,[a] Rianne M. Lord,[c,d] Robert Pal,[a] Harvey J. Smart,[a] Tameryn Stringer[c,e] and James W. Walton,*[a]

[a.] *Durham University, Department of Chemistry, Durham, DH1 3LE, UK.*
[b.] *Durham University, Department of Biosciences, Durham, DH1 3LE, UK.*
[c.] *School of Chemistry, Pharmacy and Pharmacology, University of East Anglia, Norwich, NR4 7TJ, UK.*
[d.] *Department of Chemistry, University of Warwick, Coventry, CV4 7SH, UK*
[e.] *School of Science, The University of Waikato, Hamilton 3210, New Zealand*
**contact: james.walton@durham.ac.uk*
†Supplementary Information available: [details of any supplementary information available should be included here]. See DOI: 10.1039/x0xx00000x

Contents

Synthetic Procedures: 2
- Materials: 2
- Synthetic Methods: 2
- HPLC Analysis: 21

General procedure for logP measurement: 25
General procedure for stability study: 25
Cell viability assay: 26
Reactive oxygen species study: 26
- Method 1: 2′,7′-dichlorodihydrofluorescein diacetate (H_2DCFDA) stain: 26
- Method 2: CellROX stain: 26
- Results: 28

Photophysical data for [Cu-BDP-PT)$_2$]: 32
Materials and methods for the cell localisation study: 34
Isomerisation in the Bulk Material 36
Crystallography data: 39
NMR Data 52

Supplementary Information

Synthetic Procedures:

Materials:

Commercially available reagents were purchased from Merck Life-Sciences, Fischer Scientific and Fluorochem. They were used as received. Solvents were HPLC grade or laboratory reagent grade and dried when required. Reactions requiring anhydrous conditions were carried out under an atmosphere of nitrogen using Schlenk-line techniques.

NMR spectra (^{1}H, ^{13}C{^{1}H}) were recorded on a Varian VXR-600 spectrometer (^{1}H at 599 MHz, ^{13}C{^{1}H} at 151 MHz). Spectra were recorded at 295 K in commercial deuterated solvents and referenced to the residual solvent proton resonances. Electrospray (ES), Atmospheric Pressure (ASAP) and high-resolution (HRMS) mass spectrometry were performed on an SQD mass spectrometer with Acquity UPLC.

Synthetic Methods:

Copper(2-mercapto-pyridine-1-oxide)$_2$ - [Cu(PT)$_2$]]

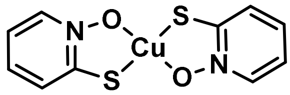

[Cu(PT)$_2$]]

2-Mercaptopyridine-*N*-oxide sodium salt (200 mg, 1.34 mmol) was dissolved in H_2O (4 ml). While stirring, a solution of copper sulphide dihydrate (114 mg, 0.67 mmol) in H_2O (6 ml) was added dropwise, and the reaction mixture was stirred for 2 h. The green precipitate that formed was collected through filtration and washed with water and ethanol. The product was dried *in vacuo* to obtain the *title compound* as a light green solid (303 mg, 72%).

m/z (ASAP HRMS^{+}) 314.9323 $[M+H]^{+\bullet}$ ($C_{10}H_9CuN_2O_2S_2$ requires 314.9316); Anal. Found (Expected) C 37.80 (38.03) H 2.51 (2.55) N 8.79 (8.87). Analytical HPLC: t_R = 7.61 min.

2-Bromo-3-methylpyridine-N-oxide, 1a

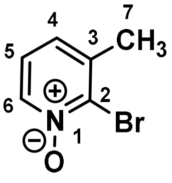

2-Bromo-3-methylpyridine (0.13 ml, 1.16 mmol) was added to dichloromethane (8 ml), and the mixture was allowed to stir at room temperature. 3-Chloroperbenzoic acid (402 mg, 2.33 mmol) was added slowly, and the reaction mixture was stirred for a further 16 h. The reaction was quenched with the addition of a saturated aqueous solution of sodium bicarbonate (approx. 10 ml), and the product was extracted with dichloromethane (3 x 10 ml). The organic layers were combined, dried over magnesium sulfide, filtered and the solvent was removed under reduced pressure. The crude brown solid was dissolved in H_2O, and the undissolved solid was removed from the filtrate through gravity filtration. The solvent was evaporated under vacuo to produce the *title compound* as a light brown solid (96 mg, 44%).

^{1}H NMR (600 MHz, D_2O) **δ** 8.21 (1H, dd, $^3J_{H\text{-}H}$ 6.5 Hz, $^4J_{H\text{-}H}$ 1.5 Hz, H^6), 7.44 (1H, dd, $^3J_{H\text{-}H}$ 7.9 Hz, $^4J_{H\text{-}H}$ 1.5 Hz, H^4), 7.30 (1H, dd, $^3J_{H\text{-}H}$ 7.9 Hz, $^3J_{H\text{-}H}$ 6.5 Hz, H^5), 2.35 (3H, s, H^7); ^{13}C{^{1}H} NMR (151 MHz, D_2O) **δ** 140.5 (C^3), 137.8 (C^6), 135.5 (C^2), 132.6 (C^4), 124.3 (C^5), 21.8 (C^7); *m/z* (ESI HRMS$^+$) 187.9713 $[M + H]^+$ ($C_6H_7{}^{79}BrNO$ requires 187.9711); R_f = 0.45 (silica, CH_2Cl_2 : 5% MeOH).

3-Methylpyridine-2-thiol-N-oxide, 1b

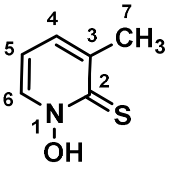

2-Bromo-3-methylpyridine-*N*-oxide (60 mg, 0.32 mmol) was added to a saturated aqueous solution of NaSH.H_2O (5 ml), and the reaction mixture was allowed to stir overnight at room temperature. The reaction was quenched with aqueous 4 M HCl (15 ml), and the hydrogen sulfide gas produced during the quenching process was trapped in an aqueous $ZnCl_2$ solution. The desired organic compound was extracted in ethyl acetate (3 x 10 ml), dried over $MgSO_4$ (anhydrous) and the solvent was evaporated under reduced pressure. Acetone (5 ml) was added

to the yellow residue, and the undissolved yellow impurity was removed by filtration. Evaporation of solvent under reduced pressure gave the *title compound* as a yellow residue, from which a sample was taken for characterisation. To avoid decomposition, the remaining compound was dissolved in aqueous NaOH (1 M, 5 ml). This solution was directly used for the next step of copper complex formation.

^{1}H NMR (600 MHz, D_2O) **δ** 7.90 – 7.78 (1H, m, H^6), 7.13 (1H, d, $^3J_{H\text{-}H}$ 7.3 Hz, H^4), 6.68 (1H, t, $^3J_{H\text{-}H}$ 7.3 Hz, H^5), 2.23 (3H, s, H^7); $^{13}C\{^1H\}$ NMR (151 MHz, D_2O) **δ** 163.8 (C^2), 140.0 (C^3), 136.2 (C^6), 129.2 (C^4), 115.6 (C^5), 22.7 (C^7); *m/z* (ESI HRMS$^+$) 142.0330 $[M + H]^+$ (C_6H_8NOS requires 142.0327).

[Copper(3-methylpyridine-2-thiolate-N-oxide)$_2$] – [Cu(3-Me-PT)$_2$]

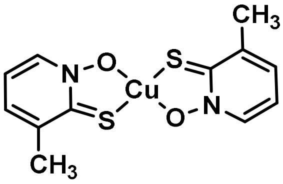

[Cu(3-Me-PT)$_2$]

To a solution (5 ml) of 3-methylpyridine-2-thiol-*N*-oxide (100 mg, 0.71 mmol) in aqueous NaOH (43 mg, 0.71 mmol), a solution of copper sulphide dihydrate (60 mg, 0.35 mmol) in H_2O (5 ml) was added dropwise. The reaction mixture was stirred for 2 h. The dark green precipitate that formed was collected by filtration and washed with water and ethanol. The crude dark green solid was dissolved in dichloromethane (3 ml). The solution was decanted from undissolved solid, and evaporation of solvent produced the *title compound* as a dark green solid (82 mg, 34%*). Single crystals of the desired complex were achieved by the layer diffusion method of dichloromethane solvent in methanol.

*yield based on two steps from 2-bromo-3-methylpyridine-N-oxide.

m/z (ASAP HRMS$^+$) 343.9712 $[M+H]^{+\bullet}$ ($C_{12}H_{13}{}^{63}CuN_2O_2S_2$ requires 343.9714). Analytical HPLC t_R = 9.08 min (>99% purity).

2-Chloro-4-methylpyridine N-oxide, 2a

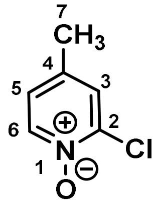

2-Chloro-4-methylpyridine (0.18 ml, 1.57 mmol) was added to dichloromethane (10 ml), and the mixture was allowed to stir at room temperature. 3-Chloroperbenzoic acid (541 mg, 3.14 mmol) was added slowly, and the reaction mixture was stirred for a further 14 h. The reaction was quenched with the addition of a saturated aqueous solution of sodium bicarbonate (approx. 10 ml), and the product was extracted with dichloromethane (3 x 20 ml). The organic layers were combined, dried over magnesium sulphide, filtered and the solvent was removed under reduced pressure. The crude brown oily liquid was purified by column chromatography on silica (CH_2Cl_2: 3% MeOH) to produce the *title compound* as a light brown solid (126 mg, 56%).

^{1}H NMR (600 MHz, Chloroform-*d*) **δ** 8.17 (1H, d, $^3J_{H-H}$ 6.8 Hz, H^6), 7.25 (1H, d, $^4J_{H-H}$ 2.4 Hz, H^3), 6.97 (1H, dd, $^3J_{H-H}$ 6.8, $^4J_{H-H}$ 2.4 Hz, H^5), 2.28 (3H, s, H^7); $^{13}C\{^1H\}$ NMR (151 MHz, Chloroform-*d*) **δ** 141.2 (C^2), 139.8 (C^6), 138.5 (C^4), 127.5 (C^3), 125.0 (C^5), 20.1 (C^7); *m/z* (ESI HRMS$^+$) 144.0213 $[M + H]^+$ ($C_6H_7{}^{35}ClNO$ requires 144.0216); Rf = 0.5 (silica, CH_2Cl_2 : 5% MeOH).

4-Methylpyridine-2-thiol-N-oxide, 2b

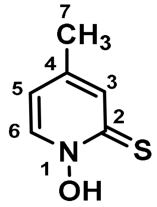

2-Chloro-4-methylpyridine-*N*-oxide (100 mg, 0.7 mmol) was added to a saturated aqueous solution of $NaSH.H_2O$ (5 ml), and the orange reaction mixture was allowed to stir overnight at room temperature. The reaction was quenched with aqueous 4 M HCl (15 ml), and the hydrogen sulfide gas produced during quenching was trapped in aqueous $ZnCl_2$ solution. The desired organic compound was extracted with ethyl acetate (3 x 10 ml), dried over $MgSO_4$ (anhydrous) and the solvent was evaporated under reduced pressure. Acetone (10 ml) was added to the yellow residue, and the undissolved yellow impurity was removed by filtration. Evaporation of solvent under reduced pressure gave the *title compound* as a yellow solid

residue, from which a sample was taken for characterisation. To avoid decomposition, the remaining compound was dissolved in aqueous NaOH (1 M, 10 ml). This solution was directly used for next step of copper complex formation.

^{1}H NMR (600 MHz, D_2O) **δ** 7.76 (1H, d, $^{3}J_{H\text{-}H}$ 6.7 Hz, H^{6}), 7.27 (1H, d, $^{4}J_{H\text{-}H}$ 2.5 Hz, H^{3}), 6.63 (1H, dd, $^{3}J_{H\text{-}H}$ 6.7 Hz, $^{4}J_{H\text{-}H}$ 2.5 Hz, H^{5}), 2.07 (3H, s, H^{7}); ^{13}C{^{1}H} NMR (151 MHz, D_2O) 162.6 (C^{2}), 141.0 (C^{4}), 137.7 (C^{6}), 132.4 (C^{5}), 119.0 (C^{3}), 19.2 (C^{7}); *m/z* (ESI HRMS^{+}) 142.0329 [M + H]$^{+}$ (C_6H_8NOS requires 142.0327).

[Copper(4-methylpyridine-2-thiolate-N-oxide)$_2$] - [Cu(4-Me-PT)$_2$]

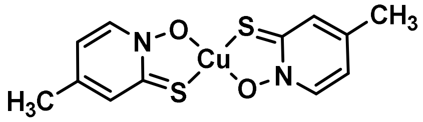

[Cu(4-Me-PT)$_2$]

To a solution (5 ml) of 4-methylpyridine-2-thiol-*N*-oxide (100 mg, 0.71 mmol) in aqueous NaOH (43 mg, 0.71 mmol), a solution of copper sulphide dihydrate (60 mg, 0.35 mmol) in H_2O (5 ml) was added dropwise. The reaction mixture was stirred for 2 h. The dark green precipitate that formed was collected by filtration and washed with water and ethanol. The crude dark green solid was dissolved in dichloromethane (3 ml). The solution was decanted from undissolved solid, and evaporation of solvent produced the *title compound* as a dark green solid (78 mg, 32%*). Single crystals of the desired complex were achieved by layer diffusion of dichloromethane solvent in methanol.

*yield based on two steps from 2-chloro-4-methylpyridine N-oxide.

m/z (ASAP HRMS^{+}) 343.9711 [M+H]$^{+\bullet}$ ($C_{12}H_{13}{}^{63}CuN_2O_2S_2$ requires 343.9714); Anal. Found (Expected) C 41.77 (41.91) H 3.50 (3.52) N 8.02 (8.15). Analytical HPLC t_R = 8.66 min (>99% purity).

2-Bromo-6-methylpyridine N-oxide, 3a

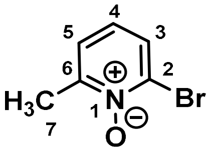

2-Bromo-6-methylpyridine (0.33 ml, 2.91 mmol) was added to dichloromethane (10 ml), and the mixture was allowed to stir at room temperature. 3-Chloroperbenzoic acid (1.00 g, 5.81 mmol) was added slowly, and the reaction mixture was stirred for a further 14 h. The reaction was quenched with the addition of a saturated aqueous solution of sodium bicarbonate (approx. 10 ml), and the product was extracted with dichloromethane (3 x 25 ml). The organic layers were combined, dried over magnesium sulphide, filtered and the solvent removed under reduced pressure. The crude yellow solid was purified by column chromatography on silica (CH_2Cl_2: 3% MeOH) to produce the *title compound* as a light brown solid (300 mg, 55%).

^{1}H NMR (600 MHz, Chloroform-*d*) δ 7.54 (1H, d, $^{3}J_{H-H}$ 8.0 Hz, H^5), 7.22 (1H, d, $^{3}J_{H-H}$ 8.0 Hz, H^3), 6.99 (1H, t, $^{3}J_{H-H}$ 8.0 Hz, H^4), 2.57 (3H, s, H^7); $^{13}C\{^{1}H\}$ NMR (151 MHz, Chloroform-*d*) δ 150.9 (C^6), 133.3 (C^2), 128.4 (C^5), 124.9 (C^3), 124.7 (C^4), 19.0 (C^7); *m/z* (ESI HRMS$^+$) 187.9715 $[M + H]^+$ ($C_6H_7{}^{79}BrNO$ requires 187.9711); R_f = 0.17 (silica, CH_2Cl_2 : 5% MeOH).

6-Methylpyridine-2-thiol-N-oxide, 3b

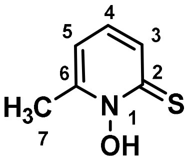

2-Bromo-6-methylpyridine-*N*-oxide (200 mg, 1.06 mmol) was added to an aqueous saturated solution of $NaSH.H_2O$ (10 ml), and the mixture was allowed to stir at room temperature for 1 h. The solution was acidified with aqueous 4 M HCl (H_2S gas generated was quenched in aqueous solution of $ZnCl_2$) and extracted with ethyl acetate (3 x 50 ml). The organic layers were combined, dried over $MgSO_4$ and the solvent was evaporated under reduced pressure. Acetone (10 ml) was added to the purple residue, and the undissolved yellow impurity was removed by filtration. Evaporation of the solvent under reduced pressure gave the *title compound* as a solid residue, from which a sample was taken for characterisation. To avoid decomposition, the remaining compound was dissolved in ethyl acetate and extracted with

aqueous NaOH (1M, 10 ml). This solution was directly used for the next step of copper complex formation.

^{1}H NMR (600 MHz, D_2O) **δ** 7.28 (1H, d, $^{3}J_{H\text{-}H}$ 7.9 Hz, H^3), 7.20 (1H, t, $^{3}J_{H\text{-}H}$ 7.9 Hz, H^4), 6.78 (1H, d, $^{3}J_{H\text{-}H}$ 7.9 Hz, H^5), 2.36 (3H, s, H^7); ^{13}C{^{1}H} NMR (151 MHz, D_2O) **δ** 166.6 (C^2), 143.0 (C^6), 132.2 (C^3), 129.1 (C^4), 115.1 (C^5), 18.1 (C^7); *m/z* (ESI HRMS$^+$) 142.0317 [M + H]$^+$ (C_6H_8NOS requires 142.0327).

[Copper(6-methylpyridine-2-thiolate-N-oxide)$_2$] - [Cu(6-Me-PT)$_2$]

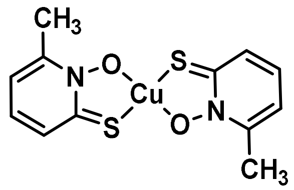

[Cu(6-Me-PT)$_2$]

To an aqueous solution (10 ml) of the sodium salt of 6-methylpyridine-2-thiol-*N*-oxide (200 mg, 1.42 mmol), a solution of copper sulphide dihydrate (121 mg, 0.71 mmol) in H_2O (5 ml) was added dropwise. The reaction mixture was stirred for 2 h. The grey precipitate that formed was collected by filtration and washed with water and ethanol. The crude grey solid obtained was purified by column chromatography on silica (CH_2Cl_2) to produce the *title compound* as a dark brown solid (120 mg, 25%*). Single crystals of the desired complex were achieved by the layer diffusion method of dichloromethane solvent in methanol.

*yield based on two steps from 2-bromo-6-methylpyridine *N*-oxide.

m/z (ASAP HRMS$^+$) 342.9611 [M]$^{+\bullet}$ ($C_{12}H_{12}{}^{63}CuN_2O_2S_2$ requires 342.9636); Anal. Found (Expected) C 42.01 (41.91) H 3.51 (3.52) N 8.04 (8.15); Analytical HPLC t_R = 9.03 min (>99% purity).

2-Bromo-3-methoxypyridine-N-oxide, 4a

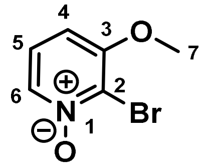

To a solution of 2-bromo-3-methoxypyridine (200 mg, 1.06 mmol) in CH_2Cl_2 (15 ml) at 0 °C, trifluoroacetic anhydride (0.3 ml, 2.13 mmol) was added dropwise. This was followed by the addition of urea hydrogen peroxide (200 mg, 2.13 mmol) at 0 °C with stirring. The reaction mixture was allowed to warm to room temperature and stirred for 14 h. The reaction was quenched with a saturated aqueous solution of $NaHCO_3$ (20 ml), and the mixture was extracted with CH_2Cl_2 (3 x 15 ml). The organic layers were combined, dried over $MgSO_4$, and the solvent was evaporated under reduced pressure. The crude brown oil was purified by column chromatography on silica (CH_2Cl_2: 3% MeOH) to produce the *title compound* as a white solid (120 mg, 55%).

^{1}H NMR (599 MHz, Chloroform-*d*) **δ** 8.08 (1H, dd, $^3J_{H\text{-}H}$ 6.6 Hz, $^4J_{H\text{-}H}$ 1.2 Hz, H^6), 7.14 (1H, dd, $^3J_{H\text{-}H}$ 8.5 Hz, $^3J_{H\text{-}H}$ 6.6 Hz, H^5), 6.77 (1H, dd, $^3J_{H\text{-}H}$ 8.5 Hz, $^4J_{H\text{-}H}$ 1.2 Hz, H^4), 3.95 (3H, s, H^7); ^{13}C{^{1}H} NMR (151 MHz, Chloroform-*d*) **δ** 156.36 (C^3), 133.6 (C^6), 126.03 (C^2), 122.8 (C^5), 107.8 (C^4), 57.0 (C^7); *m/z* (ESI HRMS$^+$) 203.9685 [M + H]$^+$ ($C_6H_7{}^{79}BrNO_2$ requires 203.9660); R_f = 0.6 (silica, CH_2Cl_2 : 10% MeOH).

3-Methoxypyridine-2-thiol-N-oxide, 4b

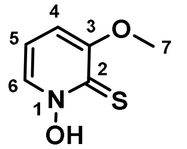

2-Bromo-3-methoxypyridine-*N*-oxide (100 mg, 0.49 mmol) was added to a saturated aqueous solution of NaSH.H_2O (5 ml), and the orange reaction mixture was allowed to stir overnight at 80 °C. The reaction was then quenched with aqueous 4M HCl (25 ml) and the hydrogen sulfide gas produced during the quenching process was trapped in aqueous $ZnCl_2$ solution. The desired organic compound was extracted in ethyl acetate (3 x 10 ml). The organic layers were combined, dried over $MgSO_4$ (anhydrous) and the solvent was evaporated under reduced pressure. Acetone (15 ml) was added to the yellow residue, and the undissolved yellow

impurity was removed by filtration. Evaporation of solvent under reduced pressure gave the *title compound* as a solid residue, from which a sample was taken for characterisation. To avoid decomposition, the remaining compound was dissolved in aqueous NaOH (1M, 15 ml). This solution was directly used for the next step of copper complex formation.

^{1}H NMR (600 MHz, D_2O) **δ** 7.63 (1H, d, $^3J_{H\text{-}H}$ 5.6 Hz, H^6), 6.76 (1H, d, $^3J_{H\text{-}H}$ 8.5 Hz, H^4), 6.70 (1H, dd, $^3J_{H\text{-}H}$ 8.5 Hz, $^3J_{H\text{-}H}$ 5.6 Hz, H^5), 3.76 – 3.63 (3H, br s, H^7); $^{13}C\{^1H\}$ NMR (151 MHz, D_2O) **δ** 157.8 (C^3), 155.8 (C^2), 131.4 (C^6), 114.7 (C^5), 108.6 (C^4), 56.2 (C^7); *m/z* (ESI HRMS$^+$) 158.0302 $[M + H]^+$ ($C_6H_8NO_2S$ requires 158.0276).

[Copper(3-methoxy-2-thiolate-N-oxide)$_2$] - [Cu(3-OMe-PT)$_2$]

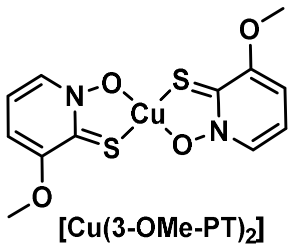

[Cu(3-OMe-PT)$_2$]

To a solution (10 ml) of the sodium salt of 3-methoxypyridine-2-thiol-*N*-oxide (100 mg, 0.63 mmol) in aqueous NaOH (25 mg, 0.63 mmol), a solution of copper chloride dihydrate (54 mg, 0.32 mmol) in H_2O (5 ml) was added dropwise. The reaction mixture was stirred for 1 h. The green precipitate that formed was collected by filtration and washed with water. The crude dark green solid obtained was dissolved in dichloromethane (5 ml). The solution was decanted from undissolved solid, and evaporation of solvent produced the *title compound* as a dark green solid (25 mg, 11%*). Single crystals of the desired complex were achieved *via* slow evaporation of dichloromethane.

*yield based on two steps from 2-bromo-3-methoxypyridine-*N*-oxide.

m/z (ASAP HRMS$^+$) 375.9618 $[M + H]^+$ ($C_{12}H_{13}{}^{63}CuN_2O_4S_2$ requires 375.9613); Anal. Found (Expected) C 38.39 (38.34) H 3.29 (3.22) N 7.22 (7.45). Analytical HPLC t_R = 7.03 min (>99% purity).

2-Chloro-4-trifluoromethylpyridine-N-oxide, 5a

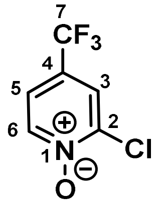

2-Chloro-4-trifluoromethylpyridine (0.36 ml, 2.75 mmol) was dissolved in a mixture of trifluoroacetic acid (5 ml) and trifluoroacetic anhydride (4.5 ml, 12 equiv.). The solution was cooled on ice, followed by the dropwise addition of 30% H_2O_2 (30% aq., 3 ml, 12 equiv.). The solution was then allowed to warm to room temperature before being heated to reflux at 80 °C overnight. The solvent was removed *in vacuo,* and the crude yellow liquid product (321 mg, 59%) was used without further purification.

^{1}H NMR (600 MHz, D_2O) **δ** 8.46 – 8.33 (1H, m, H^6), 8.07 – 7.95 (1H, m, H^3), 7.69 – 7.55 (1H, m, H^5); ^{13}C{^{1}H} NMR (151 MHz, D_2O) **δ** 142.8 (C^2), 141.3 (C^6), 131.1 (q, $^2J_{C\text{-}F}$ 36.0 Hz, C^4), 125.2 (q, $^3J_{C\text{-}F}$ 3.9 Hz, C^3), 121.7 (q, $^3J_{C\text{-}F}$ 3.6 Hz, C^5), 121.5 (q, $^1J_{C\text{-}F}$ 272.5 Hz, C^7); ^{19}F NMR (375 MHz, Chloroform-*d*) **δ** -63.7; *m/z* (ESI HRMS$^+$) 197.9938 $[M+H]^{+\bullet}$ ($C_6H_4F_3{}^{35}ClNO$ requires 197.9934).

4-Trifluoromethyl-2-thiol-N-oxide, 5b

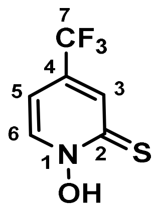

2-Chloro-4-trifluoromethylpyridine-*N*-oxide (100 mg, 1.01 mmol) was added to a saturated aqueous solution of NaSH.H_2O (5 ml), and the orange reaction mixture was allowed to stir at 80 °C overnight, until the starting material was consumed. The reaction was quenched with aqueous 4 M HCl (10 ml) and extracted with ethyl acetate (3 x 5 ml). The organic layers were combined, dried over $MgSO_4$ (anhydrous) and the solvent was evaporated under reduced pressure. Acetone (5 ml) was added to the purple residue, and the undissolved yellow impurity was removed by filtration. Evaporation of the solvent under reduced pressure gave the *title compound* as a solid residue, from which a sample was taken for characterisation. To avoid decomposition, the remaining compound was dissolved in ethyl acetate and extracted with

aqueous NaOH (1 M, 15 ml). This solution was directly used for the next step of copper complex formation.

^{1}H NMR (600 MHz, D_2O) **δ** 8.09 (1H, d, $^3J_{H\text{-}H}$ 6.9 Hz, H^6), 7.75 (1H, m, H^3), 7.05 (1H, m, H^5); ^{13}C{^{1}H} NMR (151 MHz, D_2O) **δ** 143.1 (C^2), 139.6 (C^6), 137.5 (q, $^2J_{C\text{-}F}$ 30.2 Hz, C^4), 128.9 (q, $^3J_{C\text{-}F}$ 3.0 Hz, C^3), 112.9 ($^3J_{C\text{-}F}$ 3.0 Hz C^5), 107.7 (q, $^1J_{C\text{-}F}$ 271.8 Hz, C^7); ^{19}F NMR (375 MHz, Chloroform-*d*) **δ** -64.3; *m/z* (ESI LRMS$^+$) 196.0054 [M+H]$^+$ ($C_6H_5F_3NOS$ requires 196.0044).

[Copper(4-trifluoromethyl-2-thiolate-N-oxide)₂] - [Cu(4-CF₃-PT)₂]

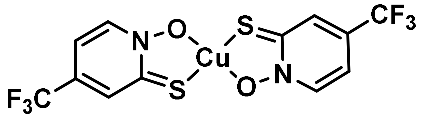

[Cu(4-CF$_3$-PT)$_2$]

To a solution (5 ml) of 4-trifluoromethylpyridine-2-thiol-*N*-oxide (200 mg, 1.02 mmol) in aqueous NaOH, a solution of copper sulphide dihydrate (87 mg, 0.51 mmol) in H_2O (10 ml) was added dropwise. The reaction mixture was stirred for 2 h. The dark green precipitate that formed was collected by filtration and washed with water and ethanol. The crude dark green solid obtained was dissolved in dichloromethane (3 ml). The solution was removed from the undissolved solid, and evaporation of solvent produced the *title compound* as a dark green solid (80 mg, 17%*).

*yield based on two steps from 2-chloro-4-trifluoromethylpyridine-*N*-oxide.

m/z (ASAP HRMS$^+$) 451.9132 [M+H]$^{+\bullet}$ ($C_{12}H_7{}^{63}CuF_6N_2O_2S_2$ requires 451.9149); Anal. Found (Expected) C 32.00 (31.9) H 1.34 (1.34) N 6.18 (6.20). Analytical HPLC t_R = 10.74 min (>99% purity).

2-Chloro-6-trifluoromethylpyridine-N-oxide, 6a

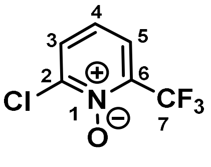

2-Chloro-6-trifluoromethylpyridine (500 mg, 2.21 mmol) was dissolved in a mixture of trifluororacetic acid (5 ml) and trifluoroacetic anhydride (5 ml, 16 equiv.). The solution was

cooled on ice, followed by the dropwise addition of 30% H_2O_2 (30% aq., 3 ml, 12 equiv.). The solution was then allowed to warm to room temperature before being heated to reflux for 45 minutes. The solvent was removed *in vacuo,* and the crude yellow oil product was used without further purification.

^{1}H NMR (600 MHz, Chloroform-*d*) **δ** 7.69 (1H, dd, $^3J_{H-H}$ 8.2 Hz, $^4J_{H-H}$ 2.0 Hz, H^3), 7.65 (1H, m, H^5), 7.29 (1H, t, $^3J_{H-H}$ 8.2 Hz, H^4); $^{13}C\{^1H\}$ NMR (151 MHz, Chloroform-*d*) **δ** 144.6 (C^2), 140.9 (q, $^2J_{C-F}$ 33.8 Hz, C^6), 129.7 (C^3) 124.1 (C^4), 123.1 (q, $^1J_{C-F}$ 4.2 Hz, C^5), 119.5 (q, $^3J_{C-F}$ 272.9 Hz, C^7); ^{19}F NMR (375 MHz, Chloroform-*d*) -69.2; *m/z* (ESI LRMS$^+$) 197.997 $[M + H]^+$ ($C_6H_4F_3{}^{35}ClNO$ requires 197.993).

6-Trifluoromethyl-2-thiol-N-oxide, 6b

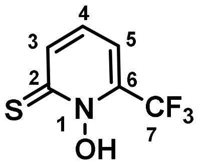

2-Chloro-6-trifluoromethylpyridine-*N*-oxide (440 mg, 2.22 mmol) was added to a saturated aqueous solution of $NaSH.H_2O$ (20 ml), and the orange reaction mixture was allowed to stir at room temperature for 3 h, until the starting material was consumed. The reaction was quenched with aqueous 4 M HCl (25 ml) and extracted with ethyl acetate (3 x 15 ml). The organic layers were combined, dried over $MgSO_4$ (anhydrous) and the solvent was evaporated under reduced pressure. Acetone (15 ml) was added to the purple residue, and the undissolved yellow impurity was removed by filtration. Evaporation of the solvent under reduced pressure gave the *title compound* as a solid residue, from which a sample was taken for characterisation. To avoid decomposition, the remaining compound was dissolved in ethyl acetate and extracted with aqueous NaOH (1 M, 15 ml). This solution was directly used for the next step of copper complex formation.

^{1}H NMR (700 MHz, D_2O) **δ** 7.69 (1H, d, $^3J_{H-H}$ 8.0 Hz, H^3), 7.27 (1H, m, H^5), 7.11 (1H, t, $^3J_{H-H}$ 8.0 Hz, H^4); $^{13}C\{^1H\}$ NMR (175 MHz, D_2O) **δ** 151.4 (C^6), 135.6 (C^3), 128.0 (C^2), 126.7 (C^5), 117.1 (C^4), 106.3 (C^7); *m/z* (ESI HRMS$^+$) 196.034 $[M+H]^+$ ($C_6H_5F_3NOS$ requires 196.004).

[Copper(6-trifluoromethyl-2-thiolate-N-oxide)$_2$] - [Cu(6-CF$_3$-PT)$_2$]

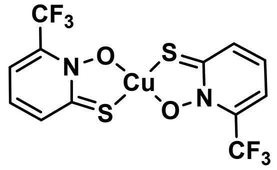

[Cu(6-CF$_3$-PT)$_2$]

To a solution of the sodium salt of 6-trifluoromethylpyridine-2-thiolate-*N*-oxide (200 mg, 1.02 mmol), a solution of copper sulphide dihydrate (87 mg, 0.51 mmol) in H_2O (5 ml) was added dropwise. The reaction mixture was stirred for 2 h. The green precipitate that formed was collected by filtration and washed with water. The crude dark green solid obtained was dissolved in dichloromethane (5 ml). Insoluble material was removed by syringe filtration before the solution was purified by column chromatography on silica (100% CH_2Cl_2) to produce the *title compound* as a dark brown solid (93 mg, 20%*). Single crystals of the product were produced *via* a slow evaporation of CH_2Cl_2.

*yield based on two steps from 2-chloro-6-trifluoromethylpyridine-*N*-oxide

m/z (ASAP HRMS$^+$) 450.9066 $[M]^{+\bullet}$ ($C_{12}H_6{}^{63}CuF_6N_2O_2S_2$ requires 450.9071); Anal. Found (Expected) C 32.12 (31.9) H 1.45 (1.34) N 6.08 (6.20); Analytical HPLC t_R = 9.80 min (>99% purity) .

2-Chloro-quinoline-N-oxide, 7a

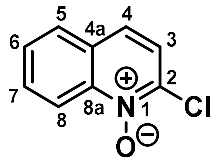

To a solution of 2-chloro-quinoline (200 mg, 1.22 mmol) in CH_2Cl_2 (10 ml) at 0 °C, trifluoroacetic anhydride (0.33 ml, 2.44 mmol) was added dropwise. This was followed by the addition of urea hydrogen peroxide (230 mg, 2.44 mmol) at 0 °C with stirring. The reaction mixture was allowed to warm to room temperature and stirred for 14 h. The reaction was quenched with a saturated aqueous solution of $NaHCO_3$ (20 ml), and the mixture was extracted with CH_2Cl_2 (3 x 30 ml). The organic layers were combined, dried over $MgSO_4$, and the solvent was evaporated under reduced pressure. The crude brown oil was purified by dissolving in H_2O. Undissolved material was removed by filtration, and the filtrate was collected, and the

solvent was evaporated under reduced pressure to give the *title compound* as a brown solid (156 mg, 70%).

^{1}H NMR (600 MHz, Chloroform-*d*) **δ** 8.75 (1H, d, $^{3}J_{H-H}$ 8.8 Hz, H^{8}), 7.88 – 7.82 (1H, m, H^{3}), 7.78 (1H, td, $^{3}J_{H-H}$ 8.8, $^{4}J_{H-H}$ 4.2, H^{7}), 7.68 – 7.58 (2H, m, H$^{4, 6}$), 7.47 (1H, dd, $^{3}J_{H-H}$ 8.8, $^{4}J_{H-H}$ 4.2 Hz, H^{5}); ^{13}C{^{1}H} NMR (151 MHz, Chloroform-*d*) **δ** 142.6 (C^{2}), 138.7 (C^{8a}), 131.1 (C^{7}), 128.7 (C^{6}), 128.7 (C^{4a}), 128.2 (C^{3}), 125.7 (C^{4}), 122.7 (C^{5}), 119.9 (C^{8}); *m/z* (ESI HRMS^{+}) 180.0214 [M+H]$^{+}$ ($C_9H_7{}^{35}ClNO$ requires 180.0216).

Quinoline-2-thio-N-oxide, 7b

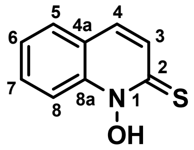

2-Chloro-quinoline-*N*-oxide (200 mg, 1.13 mmol) was added to a saturated solution of NaSH.H_2O (10 ml) and stirred at 80 ˚C for 20 h. The reaction was quenched with aqueous 4 M HCl (25 ml) and extracted with ethyl acetate (3 x 50 ml). The organic layers were combined, dried over $MgSO_4$ and the solvent was evaporated under reduced pressure. Acetone (10 ml) was added to the purple residue, and the undissolved yellow impurity was removed by filtration. Evaporation of the solvent under reduced pressure gave the *title compound* as a brown oil, from which a sample was taken for characterisation. To avoid decomposition, the remaining compound was dissolved in ethyl acetate and extracted with aqueous NaOH (1 M, 10 ml). The yellow aqueous layer was directly used for the next step of copper complex formation.

^{1}H NMR (400 MHz, D_2O) **δ** 8.20 (1H, d, $^{3}J_{H-H}$ 8.8 Hz, H^{4}), 7.80 (1H, m, H^{8}), 7.71 (1H, m, H^{5}), 7.61– 7.50 (2H, m, H^{6,7}), 7.44 (1H, d, $^{3}J_{H-H}$ 8.8 Hz, H^{3}); *m/z* (ESI HRMS^{+}) 178.0319 [M+H]$^{+\bullet}$ (C_9H_8NOS requires 178.0327).

[Copper(quinoline-2-thiolate-N-oxide)$_2$] - [Cu(Q-PT)$_2$]

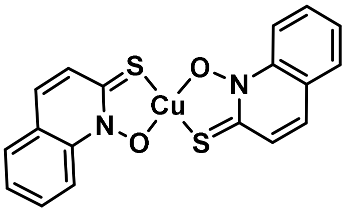

[Cu(Q-PT)$_2$]

To an aqueous solution of the sodium salt of quinoline-2-thiol-N-oxide (10 ml), a solution of copper sulphide dihydrate (114 mg, 0.71 mmol) in H_2O (5 ml) was added dropwise. The reaction mixture was stirred for 2 h at room temperature. The green precipitate that formed was collected by filtration and washed with water and ethanol. The crude light green solid obtained was purified by trituration with CH_2Cl_2. Undissolved blue solid was removed by filtration, and the solvent was removed to produce the *title compound* as a dark green solid (120 mg, 26%*).

*yield based on two steps from 2-chloro-quinoline-*N*-oxide

m/z (ASAP HRMS$^+$) 415.9714 $[M+H]^+$ ($C_{18}H_{13}{}^{63}CuN_2O_2S_2$ requires 415.9714); Anal. Found (Expected) C 51.25 (51.97) H 2.90 (2.91) N 6.41 (6.73). Analytical HPLC t_R = 10.78 min (9%) and 11.34 min (91%).

[Copper(2-hydroxypyridine-N-oxide)$_2$] - [Cu(HOPO)$_2$]

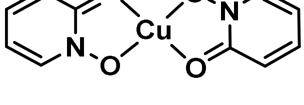

2-Hydroxypyridine-*N*-oxide (80 mg, 0.9 mmol) was dissolved in a solution of sodium hydroxide (36 mg, 0.9 mmol) in H_2O (10 ml). A solution of copper chloride dihydrate (77 mg, 0.45 mmol) in H_2O (2 ml) was added dropwise to the reaction mixture, and the reaction was stirred for 1 h. The blue precipitate that formed was collected by filtration and washed with water. The crude blue solid was dissolved in dichloromethane (3 ml). The solution was decanted from the undissolved solid, and evaporation of the solvent produced the *title compound* as a green-blue solid (77 mg, 38%).

m/z (ASAP HRMS$^+$) 283.9857 $[M+H]^+$ ($C_{10}H_9{}^{63}CuN_2O_4$ requires 283.9858); Anal. Found (Expected) **C** 42.03 (42.33) **H** 2.81 (2.84) **N** 9.68 (9.87).

2-Bromo-5-bromomethylpyridine, 8

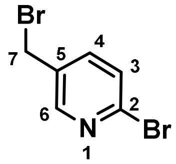

2-Bromo-5-methylpyridine (0.33 ml, 2.91 mmol) was dissolved in dry chloroform (20 ml) under N_2 atmosphere. To the solution was then added *N*-bromosuccinimide (570 mg, 3.2 mmol) and benzoyl peroxide (35 mg, 0.15 mmol), and the reaction mixture was heated at reflux for 14 h. After cooling the mixture to room temperature, the solvent was evaporated to obtain the crude product as a yellow oil. The crude product was purified by silica gel column chromatography on silica (90% hexane:10% EtOAc) to produce the *title compound* as a white solid (316 mg, 44%).

^{1}H NMR (600 MHz, Chloroform-*d*) **δ** 8.37 (1H, d, $^4J_{H\text{-}H}$ 2.5 Hz, H^6), 7.58 (1H, dd, $^3J_{H\text{-}H}$ 8.2 Hz, $^4J_{H\text{-}H}$ 2.5 Hz, H^4), 7.47 (1H, d, $^3J_{H\text{-}H}$ 8.2 Hz, H^3), 4.40 (2H, s, H^7); ^{13}C{^{1}H} NMR (151 MHz, Chloroform-*d*) **δ** 150.0 (C^6), 141.9 (C^2), 139.1 (C^4), 133.0 (C^5), 128.3 (C^3), 28.3 (C^7); *m/z* (ESI HRMS$^+$) 249.8872 [M + H]$^+$ ($C_6H_6{}^{79}Br_2N$ requires 249.8867); R_f = 0.5 (silica, Hexane : 20% EtOAc).

2-Bromo-5-(prop-2-ynoxy)pyridine, 9

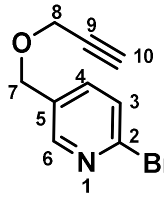

2-Bromo-5-(bromomethyl)pyridine (500 mg, 1.99 mmol) was added to a solution of 2-methoxyethanol (0.12 ml, 1.99 mmol) and sodium hydride (60% dispersion in mineral oil) (120 mg, 2.98 mmol) in dry THF (22 ml) under N_2 atmosphere at 0 °C. The reaction mixture was stirred at room temperature 16 hours under N_2 atmosphere. The reaction mixture was quenched with H_2O, and the product was extracted with EtOAc (2 x 20 ml). The organic layer was washed with H_2O (3 x 20 ml), dried over $MgSO_4$ and the solvent was evaporated under reduced pressure to produce the *title compound* as a yellow oil (370 mg, 83%).

^{1}H NMR (600 MHz, Chloroform-*d*) **δ** 8.33 (1H, d, $^{4}J_{H-H}$ 2.5 Hz, H^{6}), 7.55 (1H, dd, $^{3}J_{H-H}$ 8.1 Hz, $^{4}J_{H-H}$ 2.5 Hz, H^{4}), 7.46 (1H, d, $^{3}J_{H-H}$ 8.1 Hz, H^{3}), 4.57 (2H, s, H^{7}), 4.19 (2H, d, $^{4}J_{H-H}$ 2.4 Hz, H^{8}), 2.48 (1H, t, $^{4}J_{H-H}$ 2.4 Hz, H^{10}); $^{13}C\{^{1}H\}$ NMR (151 MHz, Chloroform-*d*) **δ** 149.6 (C^{6}), 141.6 (C^{2}), 138.1 (C^{4}), 132.3 (C^{5}), 127.8 (C^{3}), 78.6 (C^{9}), 75.2 (C^{10}), 68.1 (C^{7}), 57.4 (C^{8}); *m/z* (ESI $HRMS^{+}$) 225.9871 $[M + H]^{+}$ ($C_9H_9{}^{79}BrNO$ requires 225.9868).

2-Bromo-5-(prop-2-ynoxy)pyridine-N-oxide, 10

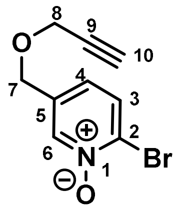

2-Bromo-5-(prop-2-ynoxy)pyridine (300 mg, 1.33 mmol) was added to chloroform (20 ml) and the mixture was allowed to stir at room temperature. 3-Chloroperbenzoic acid (687 mg, 3.99 mmol) was added slowly, and the reaction mixture was stirred for a further 16 h. The reaction was quenched with the addition of a saturated aqueous solution of sodium bicarbonate (approx. 20 ml), and the product was extracted with chloroform (3 x 15 ml). The organic layers were combined, dried over magnesium sulphide, filtered and the solvent was removed under reduced pressure. The crude brown solid was dissolved in H_2O and the undissolved solid impurities were removed by filtration. The solvent was then evaporated under reduced pressure to produce the *title compound* as a pale red solid (175 mg, 54%).

^{1}H NMR (600 MHz, Chloroform-*d*) **δ** 8.39 (1H, s, H^{6}), 7.61 (1H, d, $^{3}J_{H-H}$ 8.4 Hz, H^{4}), 7.09 (1H, d, $^{3}J_{H-H}$ 8.4 Hz, H^{3}), 4.53 (2H, s, H^{7}), 4.21 (2H, d, $^{4}J_{H-H}$ 2.4 Hz, H^{8}), 2.49 (1H, t, $^{4}J_{H-H}$ 2.4 Hz, H^{10}); $^{13}C\{^{1}H\}$ NMR (151 MHz, Chloroform-*d*) **δ** 139.3 (C^{6}), 136.0 (C^{5}), 130.4 ($C^{2,4}$), 125.0 (C^{3}), 78.5 (C^{9}), 75.8 (C^{10}), 67.2 (C^{7}), 57.9 (C^{8}); *m/z* (ESI $HRMS^{+}$) 241.9816 $[M + H]^{+}$ ($C_9H_9{}^{79}BrNO_2$ requires 241.9817)

5-(Prop-2-ynoxy)pyridine-2-thiol-N-oxide, 11

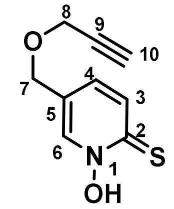

2-Bromo-5-(prop-2-ynoxy)pyridine-*N*-oxide (200 mg, 0.83 mmol) was dissolved in H_2O (5 ml). A solution of sodium sulfide (323 mg, 4.15 mmol) and sodium hydroxide (100 mg, 2.5 mmol) in H_2O (5 ml) was then added to the above solution, and the reaction mixture was stirred at 95 °C overnight. After cooling the reaction to room temperature, the yellow solution was acidified to pH 1-2 with 1 M HCl, and the product was extracted with EtOAc (3 x 20 ml). The organic layer was collected, dried over magnesium sulphide, filtered, and the solvent was removed under reduced pressure to obtain the *title compound* as a brown oil. The crude product was then directly used as a ligand for the next step of copper complex formation without further purification.

^{1}H NMR (400 MHz, Chloroform-*d*) **δ** 12.13 (1H, s, O-H), 8.14 (1H, d, $^4J_{H\text{-}H}$ 1.9 Hz, H^6), 7.68 (1H, d, $^3J_{H\text{-}H}$ 8.7 Hz, H^3), 7.27 (1H, dd, $^3J_{H\text{-}H}$ 8.7 Hz, $^4J_{H\text{-}H}$ 1.9 Hz, H^4), 4.53 (2H, s, H^7), 4.25 (2H, d, $^4J_{H\text{-}H}$ 2.4 Hz, H^8), 2.54 (1H, t, $^4J_{H\text{-}H}$ 2.4 Hz, H^{10}); *m/z* (ESI HRMS$^+$) 196.0425 [M + H]$^+$ ($C_9H_{10}NO_2S$ requires 196.0432).

[Copper(5(prop-2-ynoxy)pyridine-2-thiol-N-oxide)$_2$] - [Cu(alkyne-PT)$_2$]

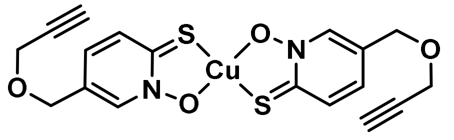

[Cu(alkyne-PT)$_2$]

5-(Prop-2-ynoxy)pyridine-2-thiol-N-oxide (600 mg, 3.07 mmol) was dissolved in a solution of sodium hydroxide (122 mg, 3.07 mmol) in H_2O (10 ml). A solution of copper chloride dihydrate (262 mg, 1.54 mmol) in H_2O (5 ml) was added dropwise to the reaction mixture and a green solid formed, which was separated by filtration. This crude product was further purified through trituration with dichloromethane as solvent. The solvent was evaporated under reduced pressure to obtain a dark green solid as the *title compound* (102 mg, 27%*).

*yield based on two steps from 2-bromo-5-(prop-2-ynoxy)pyridine-*N*-oxide.

m/z (ASAP HRMS$^+$) 451.9914 [M + H]$^+$ ($C_{18}H_{17}{}^{63}CuN_2O_4S_2$ requires 451.9926); Anal. Found (Expected) **C** 48.11 (47.83) **H** 3.48 (3.57) **N** 5.82 (6.20). Analytical HPLC: t_R = 8.41 min.

Complex [Cu(BDP-PT)₂]

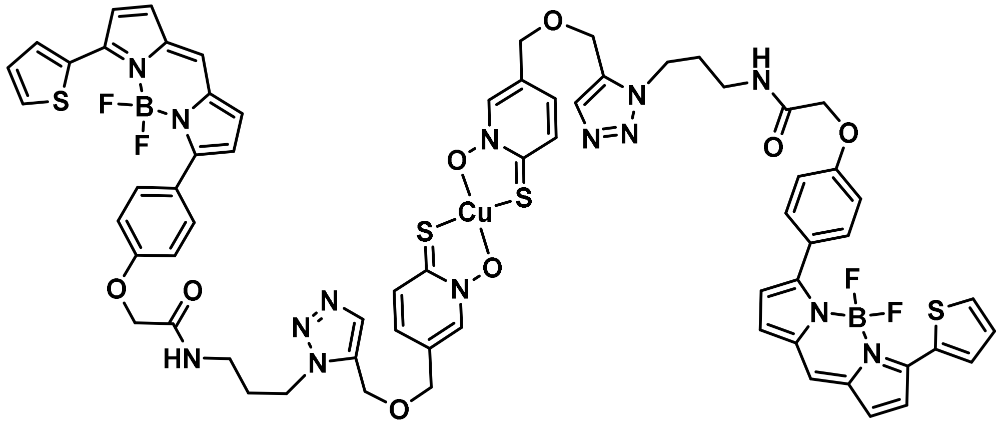

[Cu(BDP-PT)$_2$]

Copper[5(prop-2-ynoxy)pyridine-2-thiol-*N*-oxide]$_2$ (2 mg, 0.004 mmol) was dissolved in THF (1 ml). A solution of BDP-TR azide (4.5 mg, 0.01 mmol) in CH_2Cl_2 (0.2 ml) was added dropwise to the reaction mixture with stirring. Copper sulphide (1.6 mg, 0.009 mmol) and (+)sodium ascorbate (1.7 mg, 0.009 mmol) were added, and the reaction mixture was stirred at 55 °C. After 3 h, completion of the reaction was confirmed using ESI mass spectrometry. The reaction mixture was quenched with saturated aqueous solution of ammonium chloride (2 ml) and washed with brine (2 ml) and H_2O (3 ml). The organic layer was dried over anhydrous magnesium sulfide, and the solvent was evaporated under reduced pressure to obtain the crude product. Preparative high-performance liquid chromatography (HPLC) was used to purify the crude compound using the acetonitrile:H_2O solvent system as the eluent to give the *title compound* as a dark purple solid residue (3 mg, 46%).

m/z (ESI HRMS$^+$) 1464.2795 [M + H]$^+$ ($C_{66}H_{59}B_2{}^{63}CuF_4N_{14}O_8S_4$ requires 1464.2963), 1486.2576 [M + Na]$^+$ ($C_{66}H_{58}B_2{}^{63}CuF_4N_{14}NaO_8S_4$ requires 1464.2756); Analytical HPLC: t_R = 10.2 min; Photoluminescence quantum yield (MeCN) = 0.44.

HPLC Analysis:

Reverse phase HPLC analysis was performed at 298 K on a Interchim PuriFlash 4250 system, Waters XBridge Prep-C18 –19x50 mm (5 µm) column was used. A solvent system of H_2O / MeCN (gradient elution) was used. The UV/Vis and fluorescence detectors were set at appropriate wavelengths according to the species being analysed.

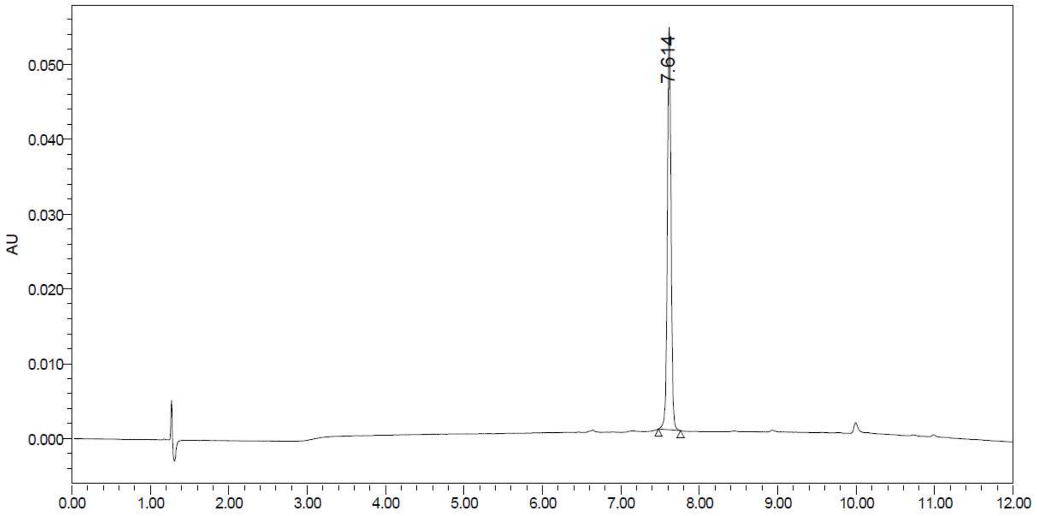

Figure S 1 Analytic HPLC trace for [Cu(PT)$_2$].

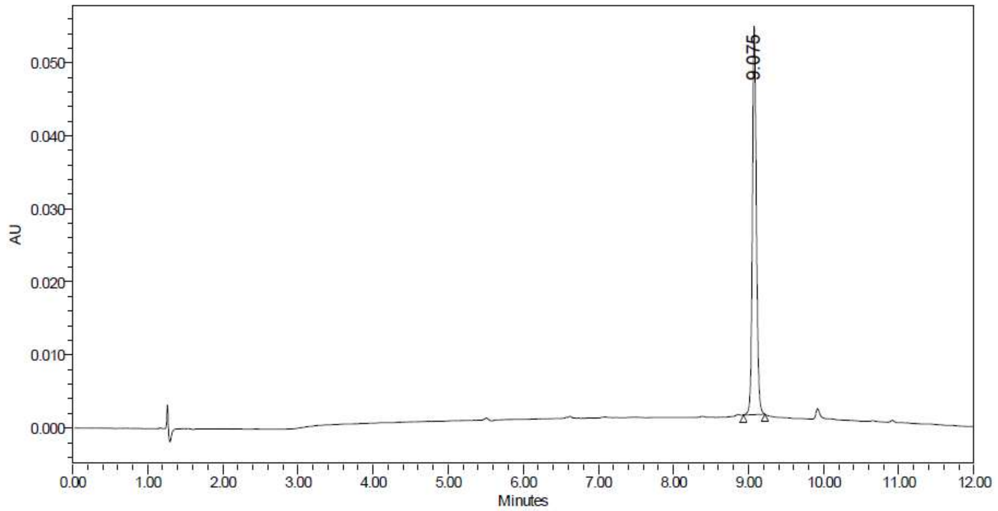

Figure S 2 Analytic HPLC trace for [Cu(3-Me-PT)$_2$].

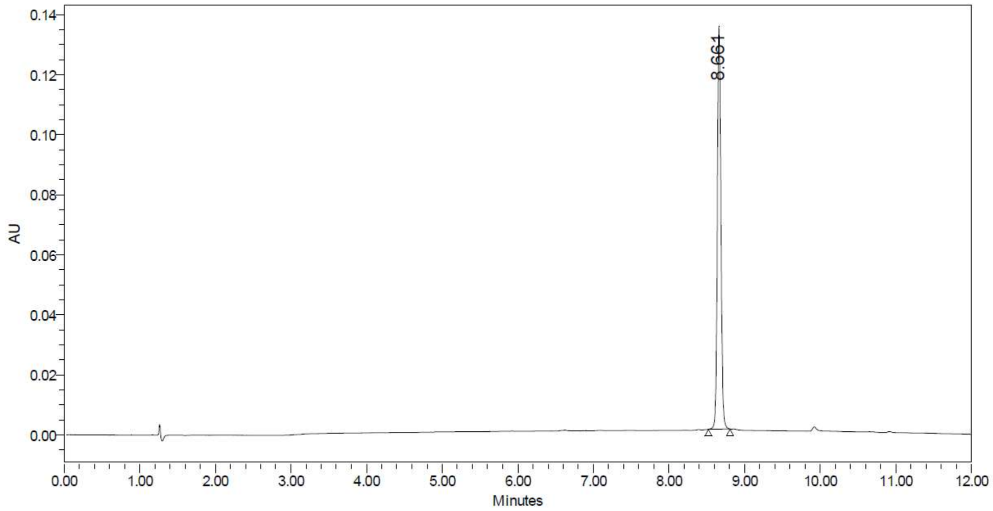

Figure S 3 Analytic HPLC trace for [Cu(4-Me-PT)$_2$].

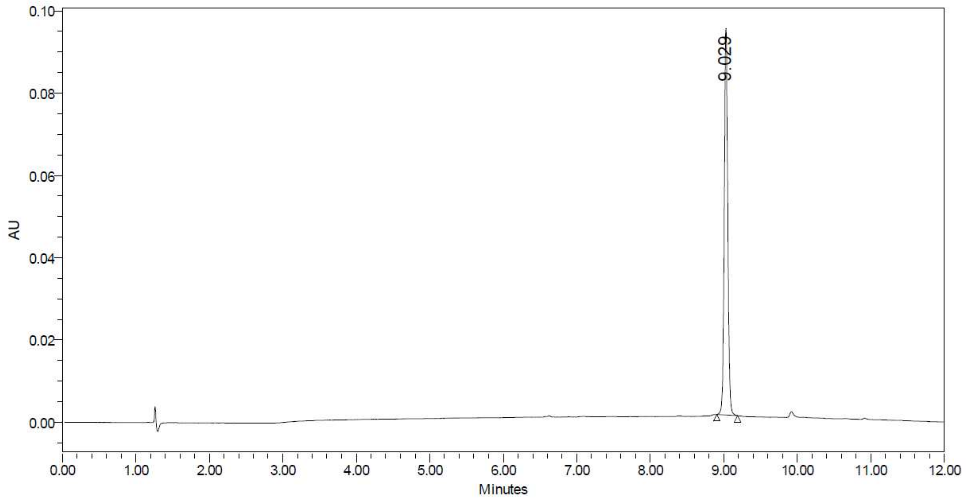

Figure S 4 Analytic HPLC trace for [Cu(6-Me-PT)$_2$].

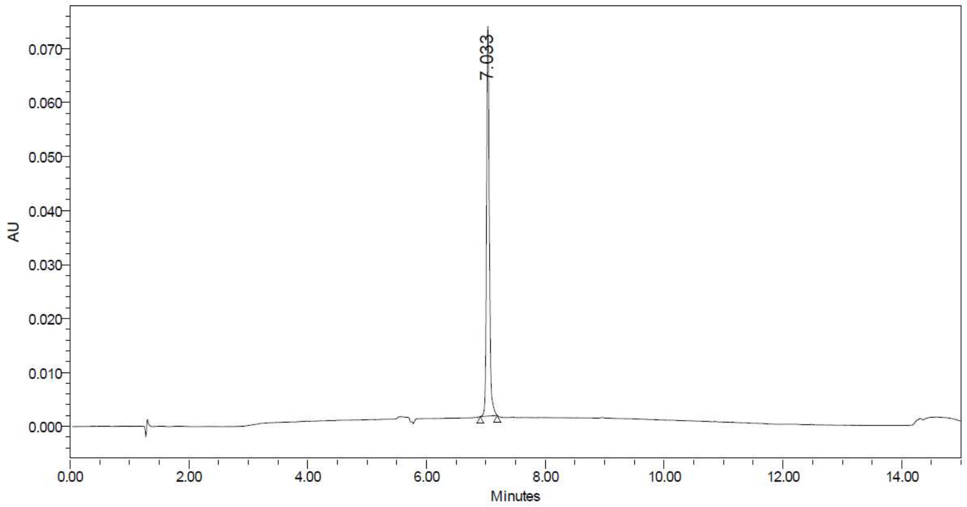

Figure S 5 Analytic HPLC trace for [Cu(3-OMe-PT)$_2$].

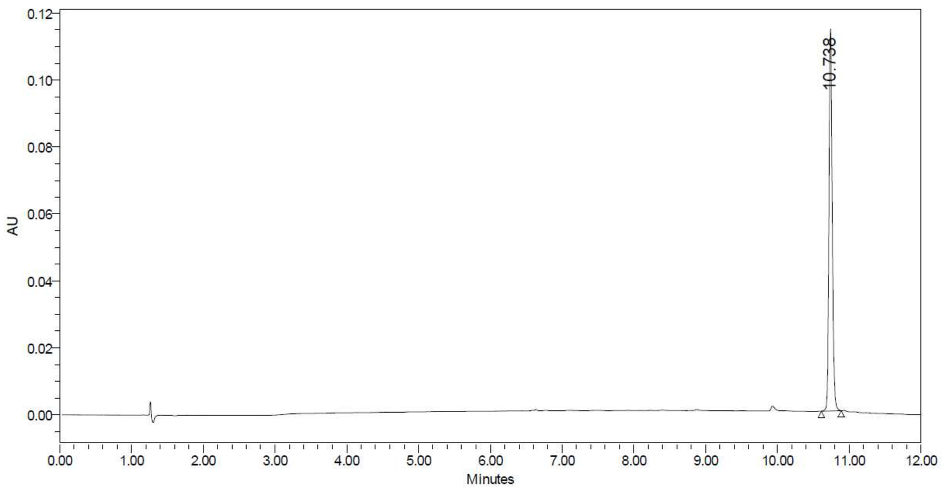

Figure S 6 Analytic HPLC trace for [Cu(4-CF$_3$-PT)$_2$].

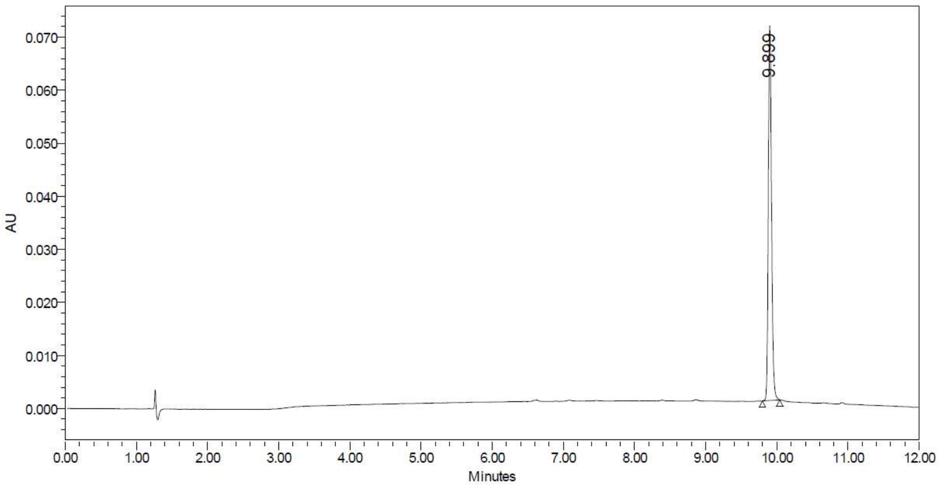

Figure S 7 Analytic HPLC trace for [Cu(6-CF$_3$-PT)$_2$].

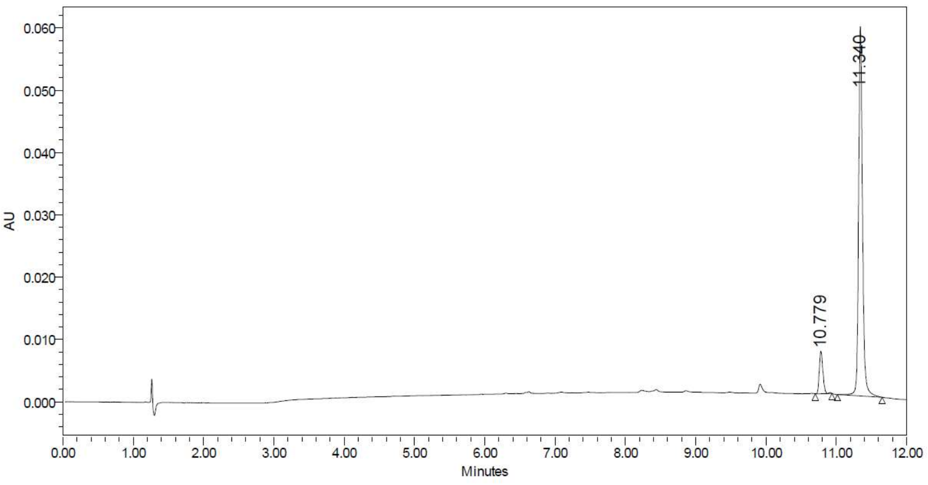

Figure S 8 Analytic HPLC trace for [Cu(Q-PT)$_2$]. Note that the peaks at 10.78 min and 11.34 min have the same mass spectrometry peaks, suggesting a mixture of cis and trans isomers that have different retention times.

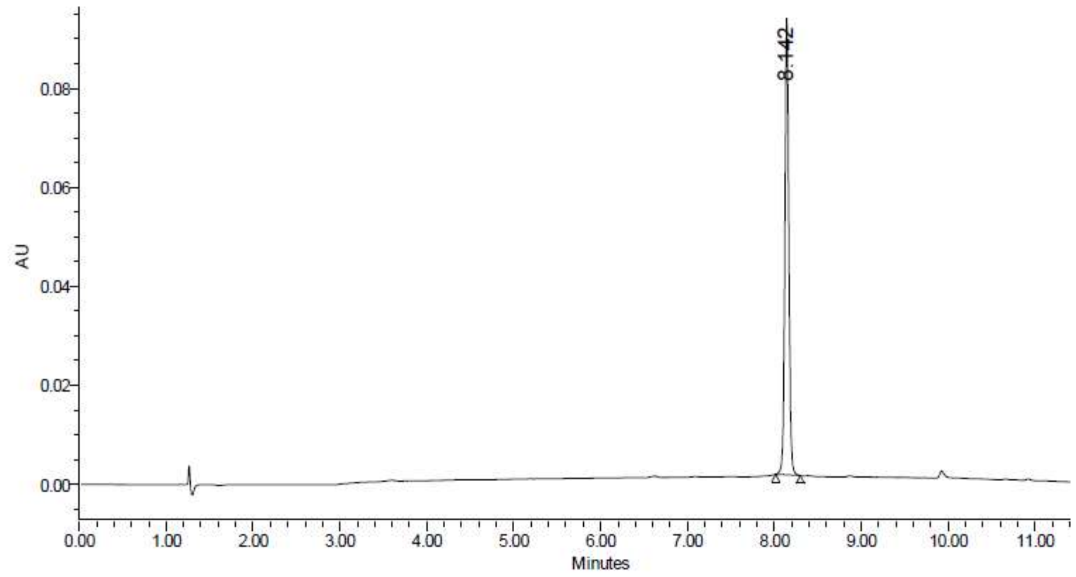

Figure S 9 Analytic HPLC trace for [Cu(alkyne-PT)$_2$].

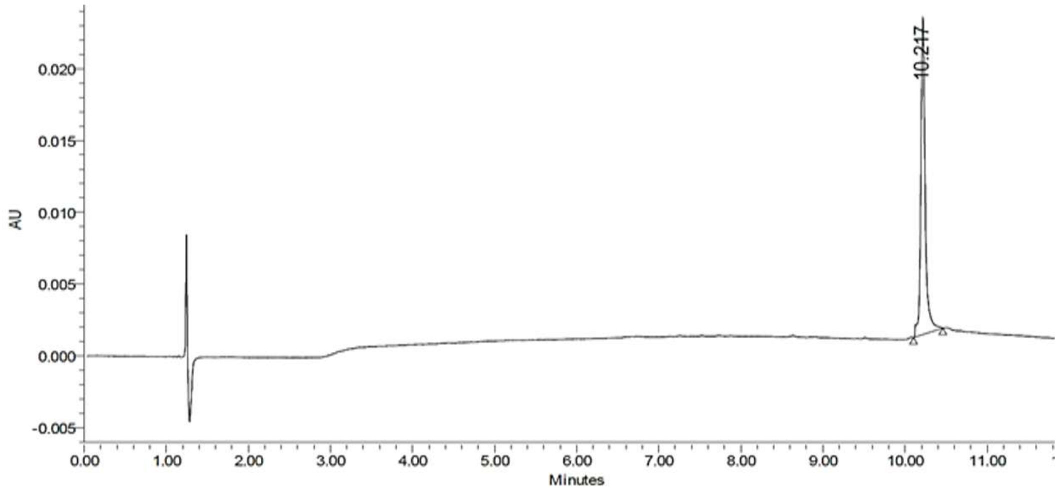

Figure S 10 Analytic HPLC trace for [Cu(BDP-PT)$_2$].

General procedure for logP measurement:

Stock solutions (10 mM) of the tested copper complexes were prepared in DMSO solvent. Water and octanol were saturated with the other solvent to match the final shake-flask conditions. Next, log P values were determined using the shake-flask method. For each complex, 60 µl of the stock solution was added to a mixture of 3 ml water and 3 ml *n*-octanol. The solution was shaken by hand and vortexed. The layers were separated and analysed by inductively coupled plasma optical emission spectroscopy (ICP-OES). For the octanol layer, 100 µl was collected in a vial and the solvent was evaporated under high vacuum. Next, the residue was dissolved in 100 µl methanol and further diluted with 10 ml of 5% aqueous nitric acid (HNO_3) solution for digestion. For the water layer, 1 ml of solution was diluted with 9 ml of 5% aqueous HNO_3 solution. After analysis through ICP-OES technique, the amount of copper in each sample was determined in ppm. To remove background copper, the exact conditions were repeated in the absence of copper pyrithione complexes and the values were subtracted from the measured values. The partition coefficient (P) and respective logP values were determined according to the following equation: All experiments and background were repeated in triplicate.

$$\log P = \log\left(\frac{[octanol]}{[water]}\right)$$

General procedure for stability study:

A stock solution of $CuCl_2.2H_2O$ (0.1 mM) was prepared in a solvent system of MeCN:H_2O = 1:1. Ligand solutions (5 mM) were prepared by combining solutions of the ligand (10 mM in MeCN:H_2O = 1:1) and NaOH (10 mM in MeCN:H_2O = 1:1) in a 1:1 ratio. The $CuCl_2.2H_2O$ solution (3.3 ml) was transferred to a cuvette and the absorption spectrum of the solution was measured using UV-vis spectrometry. Next, the basic ligand solutions were added gradually (10 µl aliquots) to the copper solution and UV-vis spectra were recorded after each addition. The addition was stopped when the spectra started to show saturation and Benessi-Hildebrand graphs were plotted using the following equation, taking the absorption maxima at wavelength 320 nm.

$$\frac{[M]_0 b}{A} = \frac{1}{K\varepsilon[L]^2} + \frac{1}{\varepsilon}$$

Where [M] is concentration of copper, [L] is concentration of the activated ligand, b is the pathlength, which was kept at 1 cm, ε is molar extinction coefficient of the complex, A is absorbance and K = binding constant.

Cell viability assay:

All cell lines were maintained as monolayers in either T75 or T175 flasks in complete high glucose DMEM media (supplemented with 10% Fetal Bovine Serum, 1% sodium pyruvate, 1% L-glutamine, 1% penstrep). All cell lines were seeded at 4000 cells/well in a clear-bottom 96-well plate and incubated for 24 h for cells to adhere. The cells were treated with varying concentrations of each complex and left to incubate for a further 24 h. MTT reagent (20 mL, 5 mg/mL) was added to each well, and the plates were incubated for 3 h. The media was removed by aspiration, followed by the addition of DMSO (150 mL) to solubilise the formazan. The absorbance was read using a Clariostar plate reader at 540 nm. Results were plotted on a logarithmic scale, and the half maximal inhibitory concentration (IC_{50}) was determined from a triplicate of triplicate repeats and reported as an IC_{50} ± Standard Deviation (SD).

Reactive oxygen species study:

Method 1: 2′,7′-dichlorodihydrofluorescein diacetate (H_2DCFDA) stain:

MCF-7 cells were seeded into a black 96 well optical bottom plate at 8000 cells/well in phenol-red free complete DMEM medium (100 µl), and incubated at 37 °C and 5% CO_2 for 24 h. After this time, the cells were treated with 1 µM and 10 µM of each complex for 1 h alongside an untreated sample (negative control). After this time, 2′,7′-dichlorodihydrofluorescein diacetate (H_2DCFDA - 20 µM – final concentration) in PBS was added for 30 min. Cells were gently washed with PBS (2 x 100 µl), and 100 µl of PBS was added to each well before reading. Fluorescence images were obtained using Zeiss Axio Observer 7 inverted microscope at excitation at 488 nm and emission at 509 nm, and analysed using ZEN 3.8 software.

Method 2: CellROX stain:

MCF-7 and ARPE cells were seeded in a black 96-well optical bottom plate (Greiner Sensoplate) at 5000 cells/well in phenol-red free complete DMEM medium (100 µl) and incubated at 37 °C and 5 % CO_2 for 24 h. Stock solutions of the compounds (5 mM) were made

in sterile DMSO (or 0.4% DMSO only for control) and diluted to 1 µM with phenol-red free complete DMEM medium (100 µl), and added to the cells for 1 h. The medium was removed, and the wells were washed with PBS (3 x 100 µl). CellROX Green (25 µM, Thermo Fisher) in phenol-red free DMEM medium was added to each well (75 µl) and incubated for 30 min. The cells were washed with PBS (3 x 100 µl) and fixed with 4% formaldehyde in PBS (100 µl/ well). After removal of the fixing agent and washing with PBS (3x 100 µl), the cells were kept in PBS (100 µL).

Comparison Menadione to [Cu(PT)$_2$]: The cells were imaged at 10x magnification (1.5385 pixels/µm) using a Zeiss Axio Observer 7 inverted fluorescence microscope equipped with a LED light source (475 nm), an EC Plan-Neofluar 10x/0.30 Ph 1 dry objective and a Hamamatsu camera. Fluorescence images are obtained (ex 450-490 nm, em 500-550 nm) with 5.5 s exposure time. All images were processed using Fiji. (*Nature Methods* 2012, 9, 676–682)

Comparison ARPE-19 to MCF-7: The cells were imaged at 10x magnification (2.2932 pixels/µm) using a Leica DMi8 inverted widefield fluorescence microscope equipped with an LED light source, a Leica HC PL APO 10x/0.45 dry objective and a K3M camera. Brightfield images were obtained at 550 nm (5 ms exposure time) using a BF filter and fluorescence images are obtained at 527 nm using a FITC green filter cube (ex 480/40 nm, dichroic 505 nm,) with 150 ms exposure time. All images were processed using Fiji. (*Nature Methods* 2012, 9, 676–682)

Results:

Incubation at 10 μM for 1 hour, following Method 1

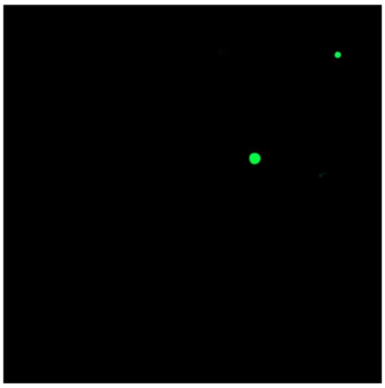

Control

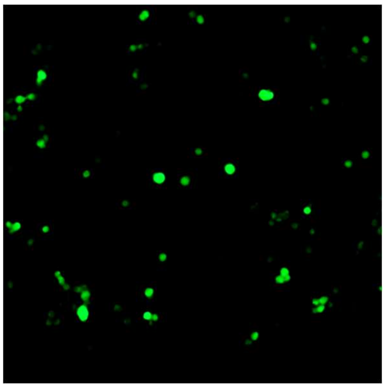

$[Cu(PT)_2]$ – 10 μM

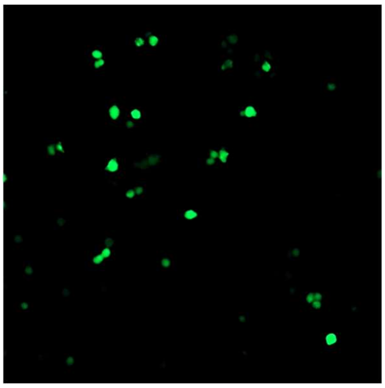

$[Cu(6\text{-}Me\text{-}PT)_2]$ – 10 μM

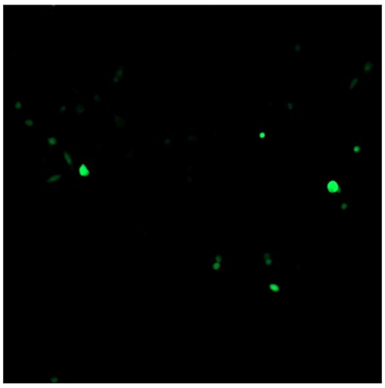

$[Cu(6\text{-}CF_3\text{-}PT)_2]$ – 10 μM

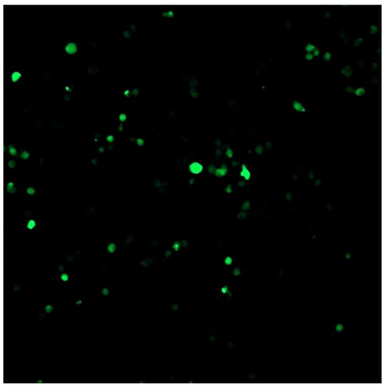

$[Cu(3\text{-}OMe\text{-}PT)_2]$ – 10 μM

Figure S 11 : Fluorescence microscopy images of the control (untreated cells) and $[Cu(PT)_2]$, $[Cu(6\text{-}Me\text{-}PT)_2]$, $[Cu(6\text{-}CF_3\text{-}PT)_2]$ and $[Cu(3\text{-}OMe\text{-}PT)_2]$ after 1 hour incubation with MCF-7 cells at 10 μM concentrations. Following Method 1, staining was conducted via a 30-minute incubation with H_2DCFDA, and images collected at a x20 magnification on a Zeiss Observer 7 inverted microscope at excitation at 488 nm and emission at 509 nm.

Dosage dependent images – 1 µM versus 10 µM at 1 hour, following Method 1

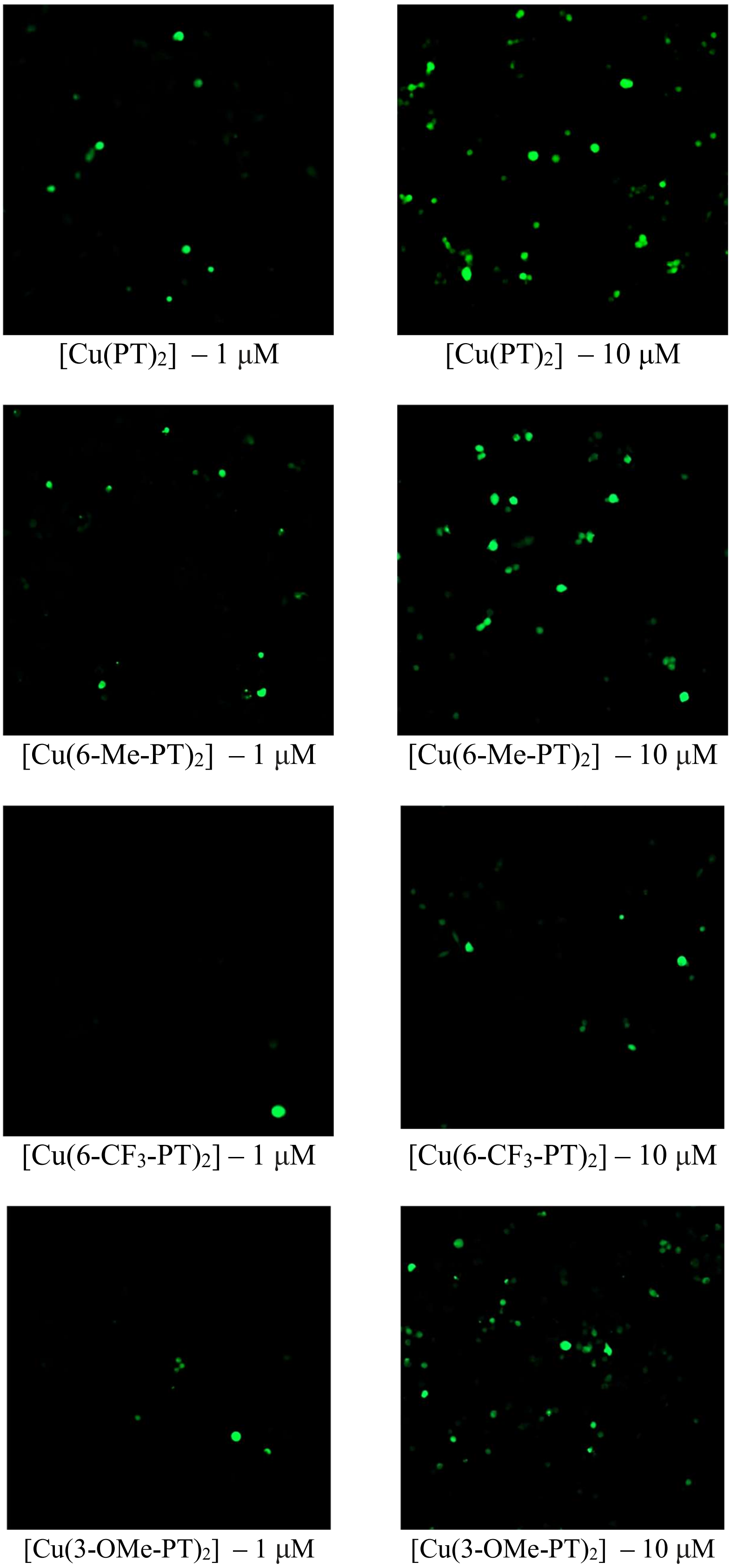

Figure S 12 Fluorescence microscopy images [Cu(PT)$_2$], [Cu(6-Me-PT)$_2$] , [Cu(6-CF$_3$-PT)$_2$] and [Cu(3-OMe-PT)$_2$] after 1 hour incubation with MCF-7 cells at both 1 µM and 10 µM concentrations. Following Method 1, staining was conducted via a 30-minute incubation with H$_2$DCFDA, and images collected at a x20 magnification on a Zeiss Observer 7 inverted microscope at excitation at 488 nm and emission at 509 nm.

ROS generation comparison between menadione and [Cu(PT)$_2$], following Method 2.

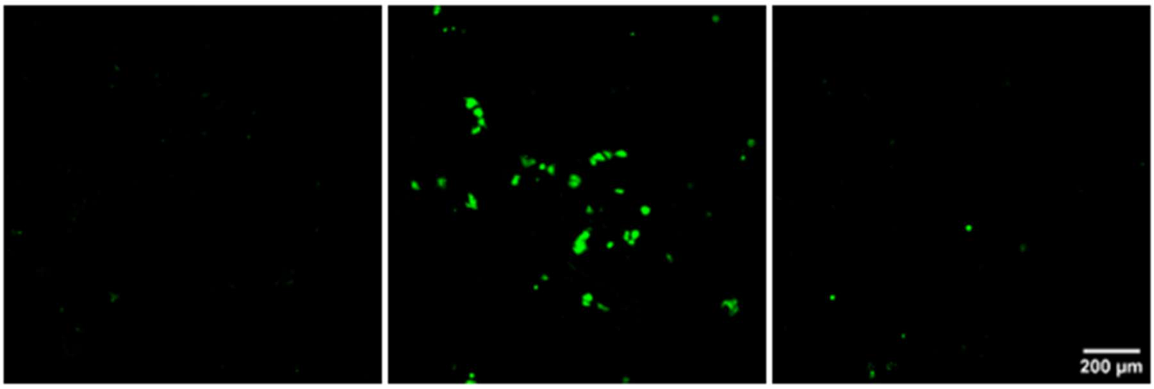

Figure S 13 Comparison of ROS formation in MCF-7 cells, showing the DMSO control (left), menadione (centre) and [Cu(PT)$_2$] (right). Following Method 2, staining was conducted via a 30-minute incubation with CellROX green and fixing with 4% PFA for 15 min. Images were collected at a x10 magnification on a Zeiss Observer 7 inverted microscope (ex 450-490 nm, em 500-550 nm).

ROS generation comparison between cancerous MCF-7 cells and healthy ARPE-19 cells, following Method 2

Table S 1 Cancerous MCF-7 cells and ARPE-19 cells dosed with selected copper complexes and stained with CellROX (Method 2), showing greater ROS generation in cancerous cells compared to healthy cells. Following Method 2, staining was conducted via a 30-minute incubation with CellROX green and fixing with 4% PFA for 15 min. Images were collected at a x10 magnification on a Leica DMi8 inverted widefield fluorescence microscope (ex 480/40 nm, em 527).

	MCF-7 Cells	ARPE-19 Cells
Control: no complex	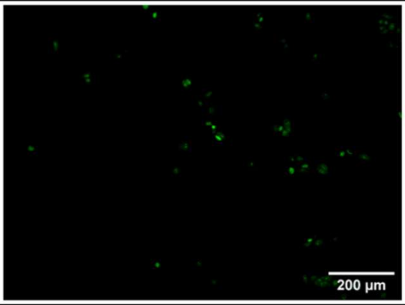	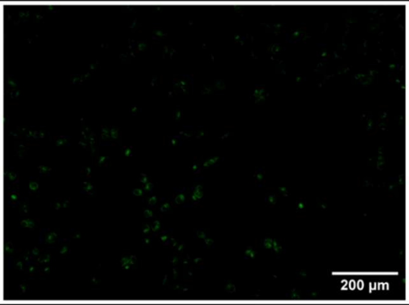
[Cu(PT)$_2$]	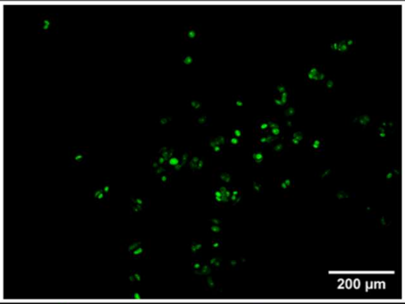	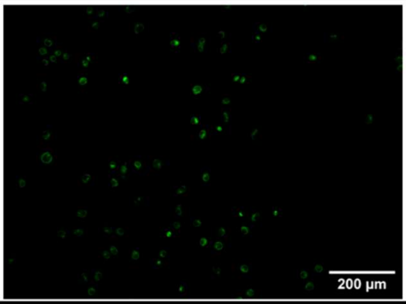
[Cu(3-Me-PT)$_2$]	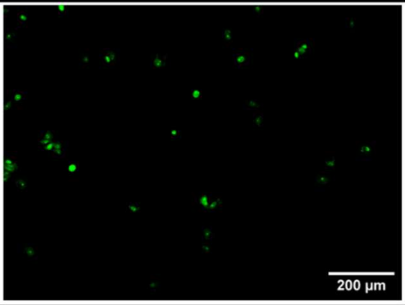	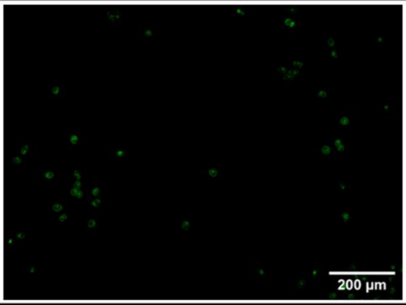
[Cu(3-OMe-PT)$_2$]	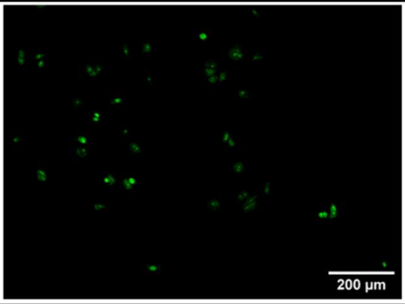	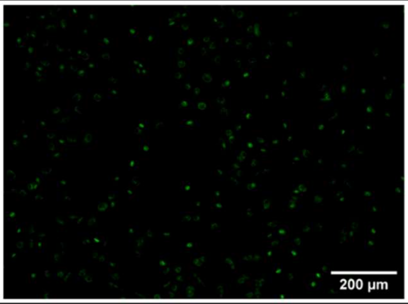
[Cu(6-CF$_3$-PT)$_2$]	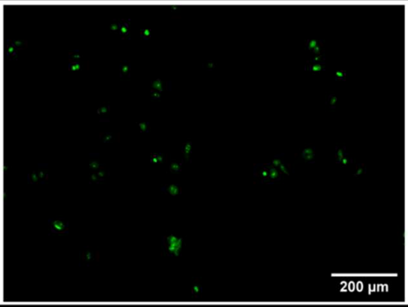	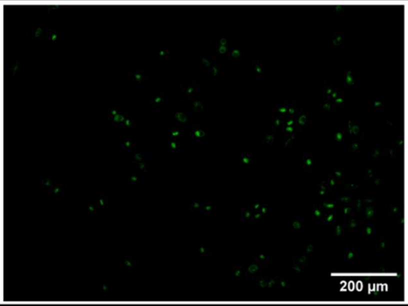

Photophysical data for [Cu-BDP-PT)$_2$]:

General Information

All samples for optical analyses were contained in quartz cuvettes with a path length of 1 cm and a polished base. Measurements were recorded at 298 K unless otherwise stated. UV-vis absorbance spectra were measured on a Cary series 5000 UV/Vis/NIR spectrophotometer using Cary WinUV v4.20 software. Samples were measured relative to a reference of pure solvent contained in a matched cell. Emission spectra were measured on a HORIBA Jobin-Yvon Fluoromax and Fluorlog using FluorEssence v3.8.0.60 software. Excitation wavelengths were selected according to the specific measurement and the excitation and emission slit width of 5 nm were used throughout. Photoluminescence quantum yield measurements were carried out using the same instrument. Measurements were performed at 298 K using solutions with absorbances of <0.1 at λ_{max}. Relative quantum yield was measured by comparison with a relevant, known standard, Rhodamine 101 (quantum yield = 1.0 in MeOH) in this case. The quantum yield was calculated by plotting the integrated emission as a function of the absorbance for both the known and unknown species according to the following equation:

$$\phi_x = \phi_s * \left(\frac{A_s}{A_x}\right) * \left(\frac{I_x}{I_s}\right) * \left(\frac{n_x}{n_s}\right)$$

Where:

- ϕ = Fluorescence quantum yield
- n = Refractive index of the solvent
- A = Absorbance of the solution
- I = Integrated fluorescence intensity of the emitted light
- Subscripts 's' and 'x' refer to the standard and unknown fluorophore respectively

Following the method described above, a photoluminescent quantum yield for [Cu(BDP-PT)$_2$] was determined as 43% in MeCN solution.

Absorption Spectrum of [Cu(BDP-PT)$_2$]

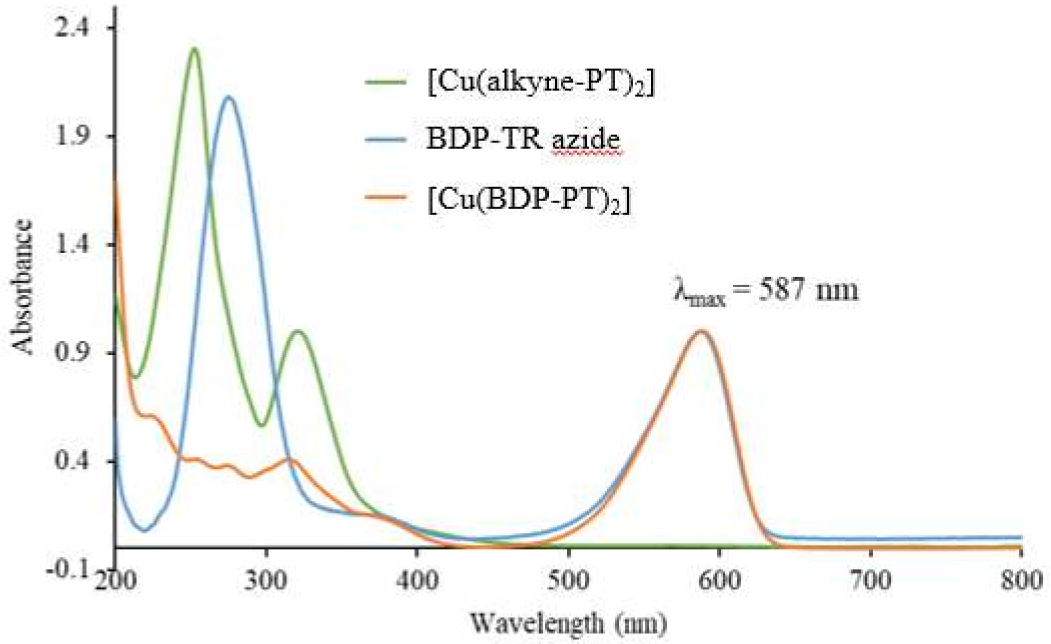

Figure S 14 Normalised UV absorbance spectra of [Cu(alkyne-PT)$_2$] (green), BDP-TR azide (blue) and [Cu(BDP-PT)$_2$] (orange) in MeCN solvent at 298 K

Molar Extinction Coefficient Calculation of [Cu(BDP-PT)$_2$]

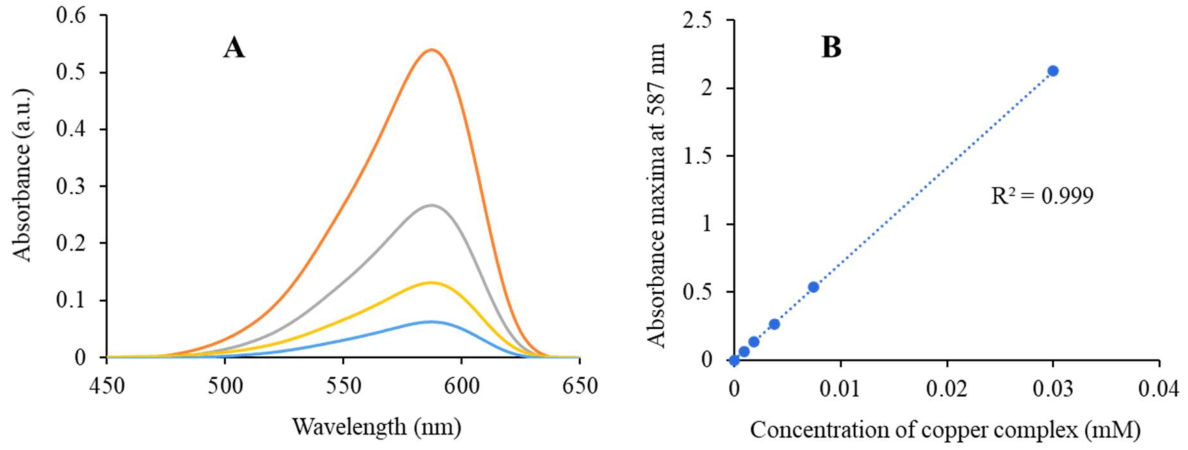

*Figure S 15 (**A**) UV absorbance spectra and (**B**) corresponding concentration graph for calculating extinction coefficient of the [Cu(BDP-PT)$_2$] in MeCN solvent, 298 K. $\varepsilon = 76800\ M^{-1}\ cm^{-1}$*

Emission Spectrum of [Cu(BDP-PT)$_2$]

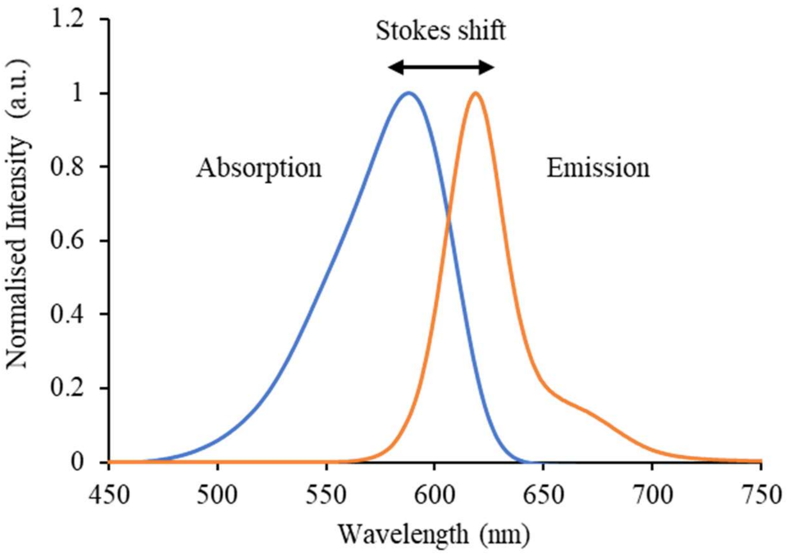

Figure S 16 Normalised absorption (blue) and emission (orange) spectra for [Cu(BDP-PT)$_2$] in MeCN solvent at 298 K. λ_{max}(em) = 619 nm.

Materials and methods for the cell localisation study:

NIH-3T3 mouse skin fibroblasts were grown as a single monolayer in Dulbecco's Modified Eagle Medium (DMEM) 1:1 supplemented with 10% foetal bovine serum (FBS). Grown in 75 cm^2 plastic culture flasks, with no prior surface treatment. Incubation was carried out at 37 °C and 5% (v:v) CO_2, and in average humidity conditions. Cell harvesting was carried out *via* washing with 10% phosphate buffered saline (PBS) prior to the addition of trypsin solution (0.25%). 5-10 minutes of incubation at 37.5 ºC was carried out prior to resuspension in fresh media by repeated aspiration with a sterile plastic pipette.

Microscopy cells were seeded in untreated iBibi 100 µl live-cell channels and allowed to grow to roughly 50% confluence, at 37 °C in 5% CO_2. Following this, DMEM was replaced, and cells were treated with the studied complex (1 µM) and additional cellular stains (ER-Tracker, Mitrotracker green 100 nM), with <0.1% DMSO present in the final imaging medium. For live-cell imaging, DMEM/F12 media (10% FBS) lacking phenol red was used from this point onward to prevent unwanted fluorescence. Following incubation, the channels were washed with live-cell imaging media and imaged using a purposely built incubator housing the microscope, maintaining 37 °C, 5% CO_2 and 10% humidity.

Steady-state fluorescence images were recorded using the PhMoNa enhanced Leica SP5 II LSCM confocal microscope equipped with a HCX PL APO 63×/1.40 NA LambdaBlue Oil immersion objective. Data were collected using 2× digital magnification at 100 Hz/line scan speed (4-line average, bidirectional scanning) at 355 nm (third harmonic NdYAG laser, set at 20 mW, 400 nJ/voxel total dwell time). In order to achieve excitation with maximal probe emission, the microscope was equipped with a triple-channel imaging detector, comprising a conventional PMT system and two HyD hybrid avalanche photodiode detectors. The frame size was determined at 1024 × 1024 pixel, with ×2 digital magnification to ensure illumination flatness of field and 0.6 airy disc unit determining the applied pinhole diameter rendering on voxel to correspond to 62 × 62 nm^2 (frame size 125 × 125 μm^2 with a section thickness set at 188 nm (at 355 nm excitation). A HeNe or Ar ion laser was used to aid parallel transmission image capture of the PI signals, used to follow the onset of necrosis. All imaging parameters are kept constant across experiments. This includes voxel size, laser power, line speed, and averaging sequences, unless otherwise noted.

All post image processing was carried out on the open source, plugin prepacked FIJI (ImageJ 1.52p Java 1.8.0_172 64 Bit). All adjustments to voxel brightness and contrast were kept at constant values within each image set. Manders' overlap coefficient (MOC) and Pearson's correlation coefficient (PCC) values were calculated using the JACoP v2.1.4 plugin for imageJ (S. Bolte and F. P. Cordelieres, *J. Microscopy*, 2006, *224*, 213). Overlap between Mitotracker Green™ and [Cu(BDP-PT)$_2$] : MOC = 0.40, PCC = 0.38. Overlap between LysoTracker Green and [Cu(BDP-PT)$_2$] : MOC = 0.45, PCC = 0.33. Overlap between ER Tracker Green™ and [Cu(BDP-PT)$_2$] : MOC = 0.87, PCC = 0.70.

Co-localisation study of [Cu(BDP-PT)$_2$] with Mitotracker Green

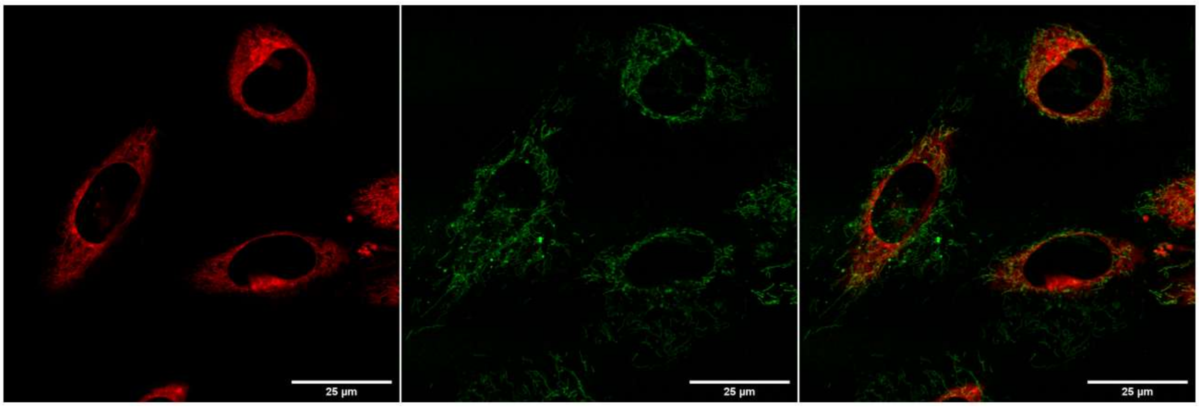

Figure S 17 Co-localisation study with [Cu(BDP-PT)$_2$] and Mitotracker Green™. Complex dosed at 1 μM for 30 min incubation. Mitotracker Green™ dosed for 5 minutes. Left: red fluorescence from [Cu(BDP-PT)$_2$] (λ_{ex} = 543 nm, λ_{em} = 600-650 nm. Middle: green fluorescence from Mitotracker Green™ (λ_{ex} = 488 nm, λ_{em} = 500 -550 nm) Right: overlayed image showing poor overlap

Co-localisation study of [Cu(BDP-PT)$_2$] with LysoTracker Green

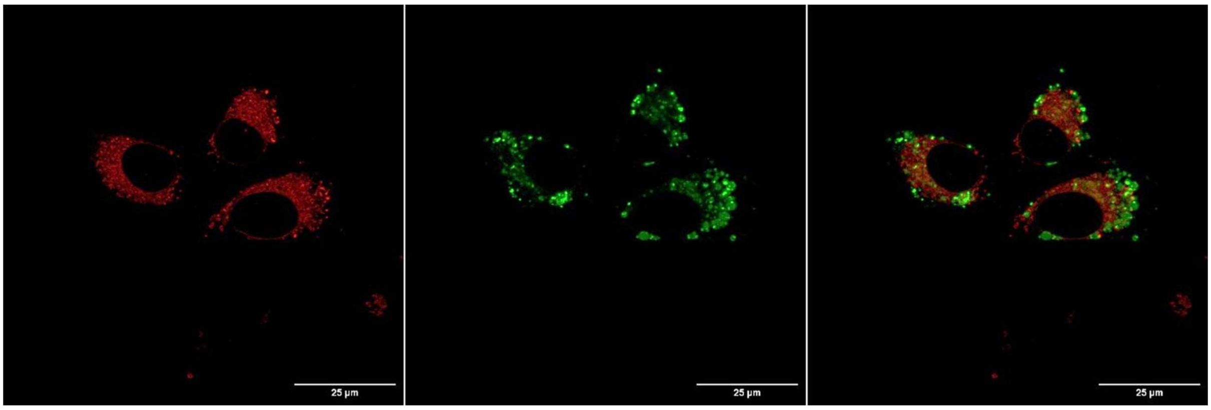

Figure S 18 Co-localisation study with [Cu(BDP-PT)$_2$] and Lysotracker Green™. Complex dosed at 1 µM for 30 min incubation. Lysotracker Green™ dosed for 5 minutes. Left: red fluorescence from [Cu(BDP-PT)$_2$] (λ_{ex} = 543 nm, λ_{em} = 600-650 nm. Middle: green fluorescence from Lysotracker Green™ (λ_{ex} = 488 nm, λ_{em} = 500 -550 nm) Right: overlayed image showing poor overlap

Isomerisation in the Bulk Material:

Density functional theory (DFT) was used to estimate the cis/trans ratio of selected copper pyrithione complexes. All DFT calculations were performed using the Gaussian 16 program package.[1] Geometry optimisations and vibrational frequency calculations were carried out using the B3LYP functional,[2-4] together with Grimme's D3 dispersion correction.[5,6] The 6-311+G(d) basis set was employed for all atoms using the default Gaussian integration grid (FineGrid, 75 radial shells × 302 angular points). Frequency calculations confirmed that all optimised structures correspond to true minima (no imaginary frequencies). Thermodynamic corrections were obtained at 298.15 K from the frequency analyses.

Both cis and trans isomers were optimised for each complex. Relative free energies (ΔG) were calculated by subtracting the energy (electronic energy + free energy corrections) of the cis isomer from that of the trans isomer. The results in Table S2 shows that in the gas state small differences in energy are observed, between 1.31 and 4.2 kJ mol^{-1}. Using the Boltzmann distribution, the energy differences were converted to percentages of cis and trans isomers. As seen, all complexes are predicted to show a mixture of cis and trans isomers in the bulk material, with small preference for cis geometry. Next, calculated IR spectra (from frequency analyses, unscaled) of isomerically pure cis-[Cu(PT)$_2$] (Figure S19) and trans-[Cu(PT)$_2$] (Figure S20) were generated. Each show two strong peaks in the region 1000-1500

cm^{-1}. This region of the spectrum corresponds to the N–O asymmetric stretch and the S–O asymmetric stretch. Note that the corresponding symmetric stretches are not observed due to little-to-no change in the dipole moment. The FTIR spectrum of the experimental bulk sample of $[Cu(PT)_2]$ was then measured (Figure S21). In this experimental sample, four peaks are present in the region 1000-1500 cm^{-1}, which implies that the bulk sample is a mixture of cis and trans isomers, in line with the prediction from computation.

1. M. J. Frisch, G. W. Trucks, H. B. Schlegel, G. E. Scuseria, M. A. Robb, J. R. Cheeseman *et al.*, *Gaussian 16, Revision C.01*, Gaussian, Inc., Wallingford CT, 2016.
2. A. D. Becke, *Phys. Rev. A*, 1988, **38**, 3098–3100.
3. C. Lee, W. Yang and R. G. Parr, *Phys. Rev. B*, 1988, **37**, 785–789.
4. P. J. Stephens, F. J. Devlin, C. F. Chabalowski and M. J. Frisch, *J. Phys. Chem.*, 1994, **98**, 11623–11627.
5. S. Grimme, J. Antony, S. Ehrlich and H. Krieg, *J. Chem. Phys.*, 2010, **132**, 154104.
6. S. Grimme, S. Ehrlich and L. Goerigk, *J. Comput. Chem.*, 2011, **32**, 1456–1465.

Table S 2 DFT computational estimate of energy difference between cis and trans isomers of all complexes. Conversion to cis/trans percentages using the Boltzmann distribution.

Complex	ΔG (kJ/mol)	% cis	% trans
$[Cu(PT)_2]$	4.20	84	16
$[Cu(3\text{-}Me\text{-}PT)_2]$	3.94	83	17
$[Cu(4\text{-}Me\text{-}PT)_2]$	2.36	72	28
$[Cu(6\text{-}Me\text{-}PT)_2]$	2.36	72	28
$[Cu(3\text{-}OMe\text{-}PT)_2]$	3.41	80	20
$[Cu(4\text{-}CF_3\text{-}PT)_2]$	2.36	72	28
$[Cu(6\text{-}CF_3\text{-}PT)_2]$	2.10	70	30
$[Cu(Q\text{-}PT)_2]$	1.31	63	37

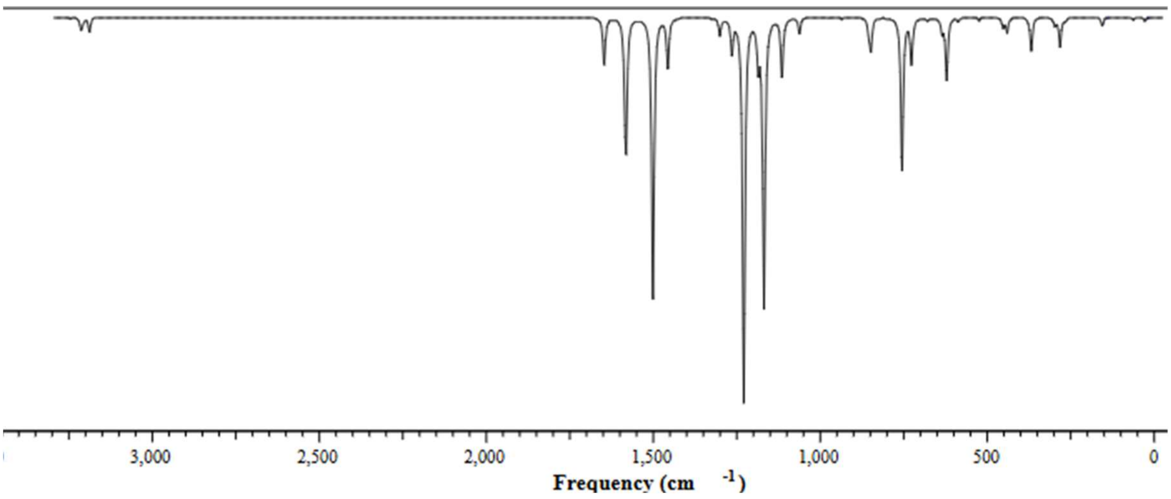

Figure S 19 Predicted FTIR spectrum of cis-[Cu(PT)$_2$], with two strong peaks in the region 1000-1500 cm^{-1}, corresponding to the N–O asymmetric stretch and the S–O asymmetric stretch.

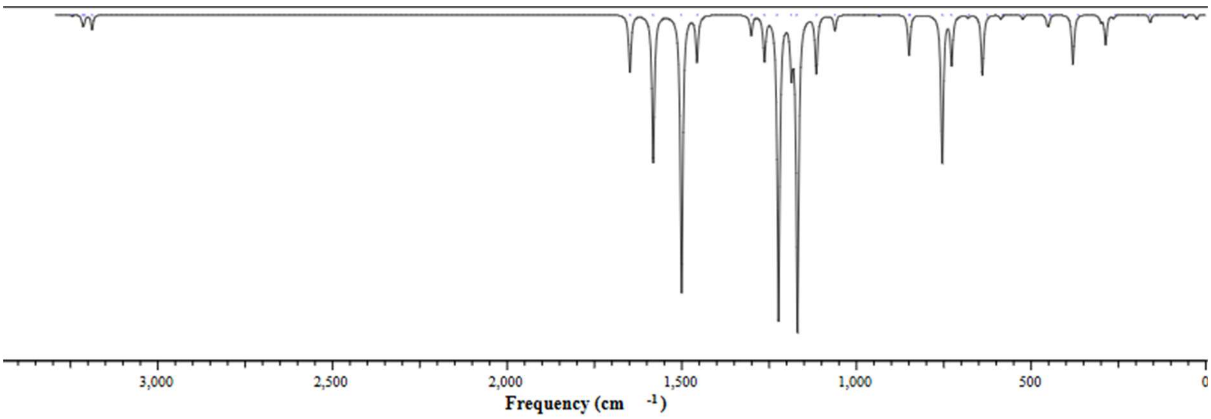

Figure S 20 Predicted FTIR spectrum of trans-[Cu(PT)$_2$], with two strong peaks in the region 1000-1500 cm^{-1}, corresponding to the N–O asymmetric stretch and the S–O asymmetric stretch

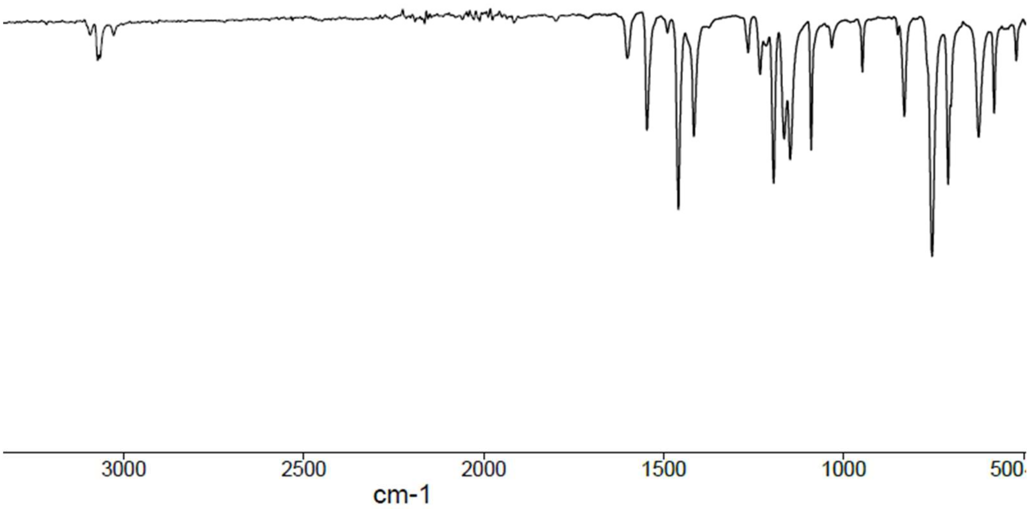

Figure S 21 Experimentally measured FTIR spectrum of bulk sample of [Cu(PT)$_2$], showing four peaks in the 1000-1500 cm^{-1} indicating a mixture of cis and trans complexes in the bulk.

Crystallography data:

The X-ray single crystal data have been collected for [Cu(3-Me-PT)$_2$] (CCDC-2377353), [Cu(4-Me-PT)$_2$] (CCDC-2377357), [Cu(6-Me-PT)$_2$] (CCDC- 2377356), *cis*-[Cu(3-OMe-PT)$_2$] (CCDC-2377352), *cis-trans*-[Cu(3-OMe-PT)$_2$] (CCDC-2377355) and [Cu(6-CF$_3$-PT)$_2$] (CCDC-2377354) using MoKα radiation (λ =0.71073Å) on an Bruker D8 Venture (Photon III MM C7 CPAD detector, IμS micro-source, focusing mirrors) 3-circle diffractometer equipped with a Cryostream-700 (Oxford Cryosystems) open-flow nitrogen cryostat at the temperature 130.0 (2)K. The structure was solved by direct method and refined by full-matrix least squares on F^2 for all data using Olex2[1] and SHELXTL[2] software. All non-hydrogen atoms were refined in an anisotropic approximation; hydrogen atoms were placed in the calculated positions and refined in riding mode. Crystal data and parameters of refinement are listed below. Some views of crystal packing are given in the Figures below.

1. O. V. Dolomanov, L. J. Bourhis, R. J. Gildea, J. A. K. Howard and H. Puschmann, J. *Appl. Cryst.* (2009), **42**, 339-341.

2. G.M. Sheldrick, *Acta Cryst.* (2008), **A64**, 112-122

Data for Complex [Cu(6-CF_3-PT)$_2$]

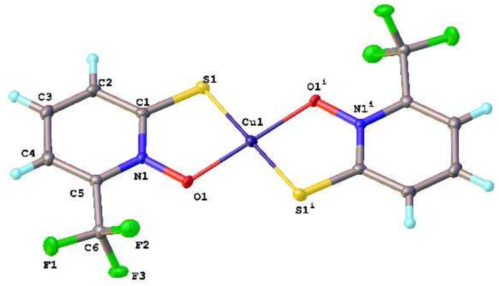

Table S 3 Crystal data and structure refinement for [Cu(6-CF_3-PT)$_2$]

Empirical formula	$C_{12}H_6CuF_6N_2O_2S_2$
Formula weight	451.85
Temperature/K	120.0
Crystal system	triclinic
Space group	P-1
a/Å	4.4334(2)
b/Å	12.6398(6)
c/Å	13.3645(7)
α/°	98.982(2)
β/°	94.392(2)
γ/°	90.911(2)
Volume/Å^3	737.26(6)
Z	2
ρ_{calc}g/cm^3	2.035
μ/mm^{-1}	1.844
F(000)	446.0
Crystal size/mm^3	0.32 × 0.05 × 0.03
Radiation	Mo Kα (λ = 0.71073)
2Θ range for data collection/°	4.128 to 58.998
Index ranges	$-6 \leq h \leq 6$, $-17 \leq k \leq 17$, $-18 \leq l \leq 18$
Reflections collected	15705
Independent reflections	4100 [R_{int} = 0.0550, R_{sigma} = 0.0520]
Data/restraints/parameters	4100/0/229
Goodness-of-fit on F^2	1.048
Final R indexes [I>=2σ (I)]	R_1 = 0.0297, wR_2 = 0.0709
Final R indexes [all data]	R_1 = 0.0426, wR_2 = 0.0754
Largest diff. peak/hole / e Å^{-3}	0.48/-0.47

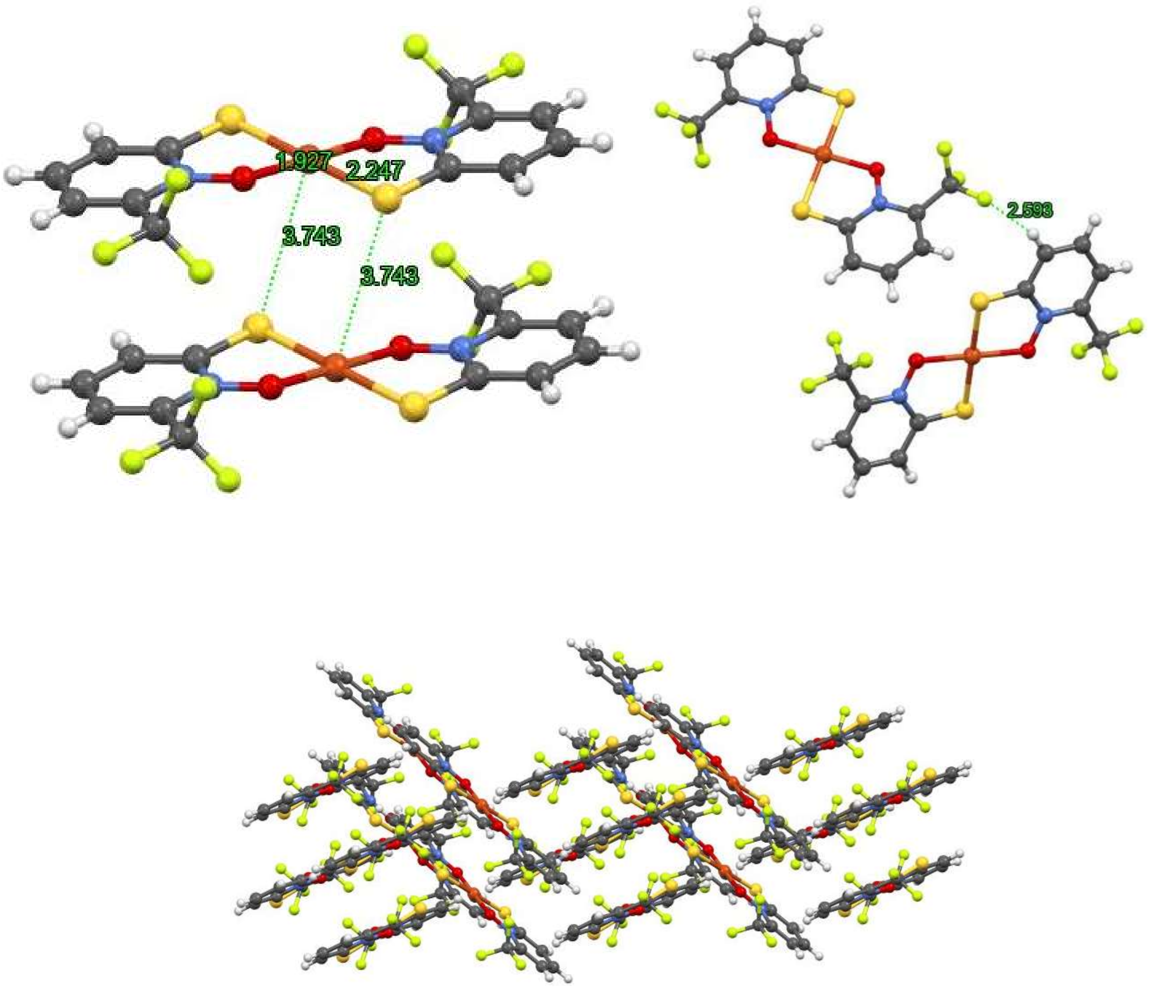

Figure S 22 Crystal packing structures of [Cu(6-CF_3-PT)$_2$].

Data for Complex [Cu(3-Me-PT)$_2$]

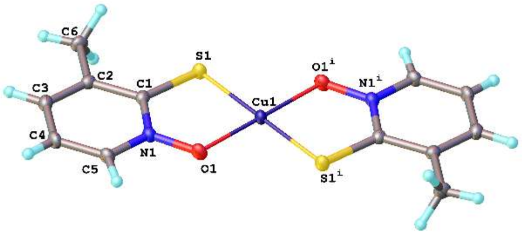

Table S 4 Crystal data and structure refinement for [Cu(3-Me-PT)$_2$]

Empirical formula	$C_{12}H_{12}N_2O_2S_2Cu$
Formula weight	343.90
Temperature/K	120.0
Crystal system	triclinic
Space group	P-1
a/Å	3.9634(2)
b/Å	7.5851(4)
c/Å	10.8927(6)
α/°	82.893(2)
β/°	86.756(2)
γ/°	80.578(2)
Volume/Å^3	320.34(3)
Z	1
ρ_{calc}g/cm^3	1.783
μ/mm^{-1}	2.027
F(000)	175.0
Crystal size/mm^3	0.14 × 0.13 × 0.02
Radiation	Mo Kα (λ = 0.71073)
2Θ range for data collection/°	7.006 to 59.988
Index ranges	$-5 \leq h \leq 5$, $-10 \leq k \leq 10$, $-15 \leq l \leq 15$
Reflections collected	5538
Independent reflections	1859 [R_{int} = 0.0255, R_{sigma} = 0.0288]
Data/restraints/parameters	1859/0/112
Goodness-of-fit on F^2	1.078
Final R indexes [I>=2σ (I)]	R_1 = 0.0295, wR_2 = 0.0697
Final R indexes [all data]	R_1 = 0.0332, wR_2 = 0.0711
Largest diff. peak/hole / e Å^{-3}	0.94/-0.44

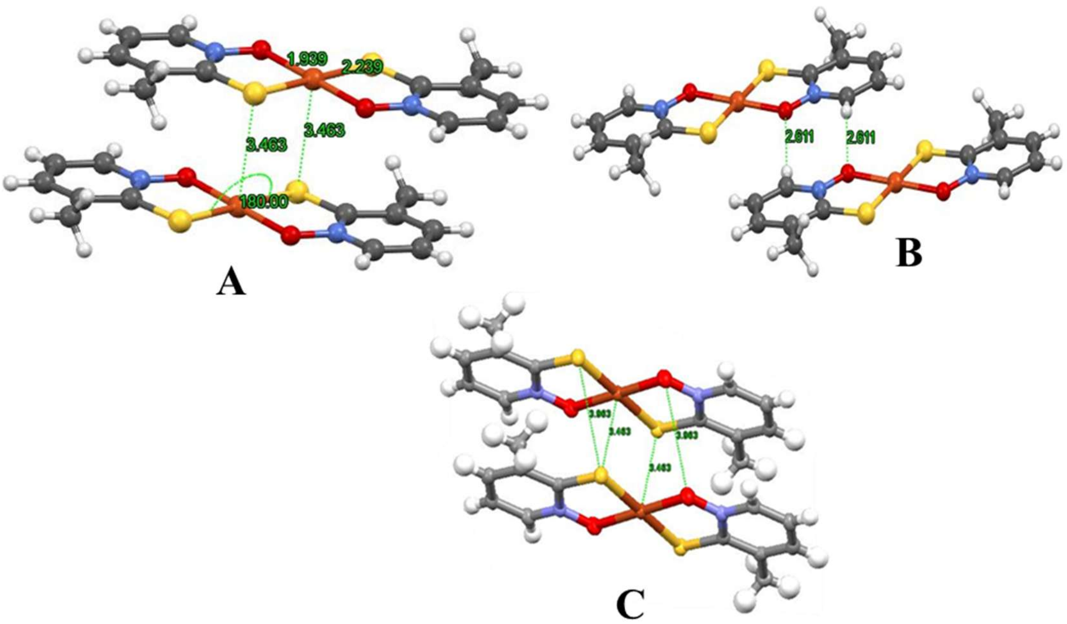

Figure S 23 Crystal packing structure of [Cu(3-Me-PT)$_2$].

Data for Complex [Cu(4-Me-PT)$_2$]

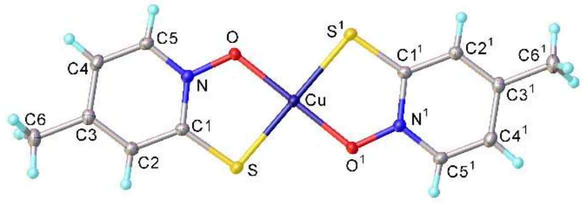

Table S 5 Crystal data and structure refinement for [Cu(4-Me-PT)$_2$]

Empirical formula	$C_{12}H_{12}N_2O_2S_2Cu$
Formula weight	343.90
Temperature/K	120.0
Crystal system	triclinic
Space group	P-1
a/Å	4.1289(3)
b/Å	7.5872(5)
c/Å	10.9528(7)
α/°	10.9528(7)
β/°	89.980(3)
γ/°	83.141(3)
Volume/Å^3	323.88(4)
Z	1
ρ_{calc}g/cm^3	1.763
μ/mm^{-1}	2.005
F(000)	175.0
Crystal size/mm^3	0.265 × 0.082 × 0.081
Radiation	Mo Kα (λ = 0.71073)
2Θ range for data collection/°	5.82 to 69.914
Index ranges	$-6 \le h \le 6$, $-12 \le k \le 12$, $-17 \le l \le 17$
Reflections collected	7639
Independent reflections	2818 [R_{int} = 0.0320, R_{sigma} = 0.0424]
Data/restraints/parameters	2818/0/112
Goodness-of-fit on F^2	1.061
Final R indexes [I>=2σ (I)]	R_1 = 0.0334, wR_2 = 0.0701
Final R indexes [all data]	R_1 = 0.0471, wR_2 = 0.0747
Largest diff. peak/hole / e Å^{-3}	0.54/-0.53

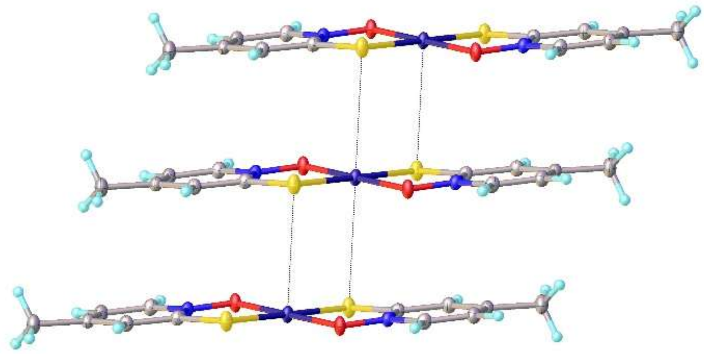

Figure S 24 Crystal packing structure of [Cu(4-Me-PT)$_2$].

Data for Complex *trans*-[Cu(6-Me-PT)₂]

[Cu(6-Me-PT)$_2$] shows both *cis* and *trans* isomers as separate crystals within the same crystallisation vial. The *cis* isomer appears as brown, plate-shaped crystals that make up the majority of crystals that form, while the *trans* isomer also forms as a minor species, appearing as small, iridescent plates. The *trans* isomer data is given below. The cis isomer data was lower quality, but the structure has been previously published.[3]

(3) D.X.West, C.A.Brown, J.P.Jasinski, J.M.Jasinski, R.M.Heathwaite, D.G.Fortier, R.J.Staples, R.J.Butcher, *Journal of Chemical Crystallography*, 1998, 28, 853, DOI: 10.1023/A:1022886116342

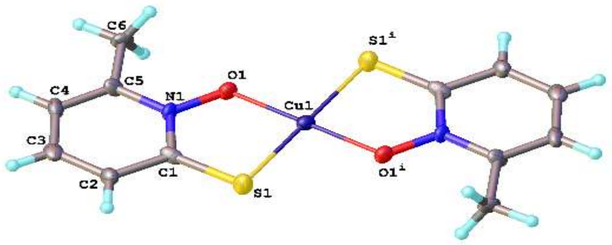

Table S 6 Crystal data and structure refinement for trans-[Cu(6-Me-PT)$_2$]

Empirical formula	$C_{12}H_{12}CuN_2O_2S_2$
Formula weight	343.90
Temperature/K	120.0
Crystal system	monoclinic
Space group	$P2_1/c$
a/Å	7.478(3)
b/Å	13.630(5)
c/Å	6.982(2)
α/°	90
β/°	106.895(9)
γ/°	90
Volume/Å^3	680.9(4)
Z	2
ρ_{calc}g/cm^3	1.677
μ/mm^{-1}	1.907
F(000)	350.0
Crystal size/mm^3	0.14 × 0.08 × 0.005

Radiation	MoKα (λ = 0.71073)
2Θ range for data collection/°	5.694 to 52.976
Index ranges	-9 ≤ h ≤ 9, -17 ≤ k ≤ 17, -8 ≤ l ≤ 8
Reflections collected	10114
Independent reflections	1416 [R_{int} = 0.1711, R_{sigma} = 0.1211]
Data/restraints/parameters	1416/0/89
Goodness-of-fit on F^2	1.095
Final R indexes [I>=2σ (I)]	R_1 = 0.0976, wR_2 = 0.2402
Final R indexes [all data]	R_1 = 0.1806, wR_2 = 0.2772
Largest diff. peak/hole / e $Å^{-3}$	2.70/-0.85

Data for Complex *cis*-[Cu(3-OMe-PT)$_2$]

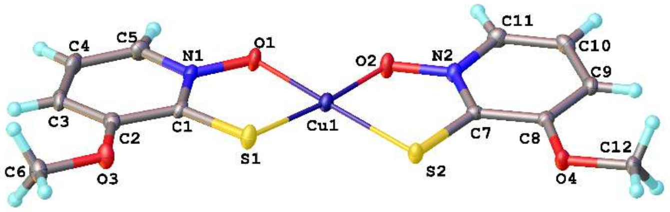

Table S 7 Crystal data and structure refinement for cis-[Cu(3-OMe-PT)$_2$]

Empirical formula	$C_{12}H_{12}CuN_2O_4S_2$
Formula weight	375.90
Temperature/K	120.0
Crystal system	triclinic
Space group	P-1
a/Å	7.0485(2)
b/Å	7.2975(2)
c/Å	14.1070(5)
α/°	78.535(2)
β/°	78.6840(10)
γ/°	79.0980(10)
Volume/Å^3	688.75(4)
Z	2
ρ_{calc}g/cm^3	1.813
μ/mm^{-1}	1.905
F(000)	382.0
Crystal size/mm^3	0.21 × 0.06 × 0.03
Radiation	MoKα (λ = 0.71073)
2Θ range for data collection/°	5.768 to 59.99
Index ranges	$-9 \le h \le 9$, $-10 \le k \le 10$, $-19 \le l \le 19$
Reflections collected	10359
Independent reflections	3987 [R_{int} = 0.0370, R_{sigma} = 0.0509]
Data/restraints/parameters	3987/0/238
Goodness-of-fit on F^2	1.050
Final R indexes [I>=2σ (I)]	R_1 = 0.0410, wR_2 = 0.0754
Final R indexes [all data]	R_1 = 0.0549, wR_2 = 0.0797
Largest diff. peak/hole / e Å^{-3}	0.45/-0.41

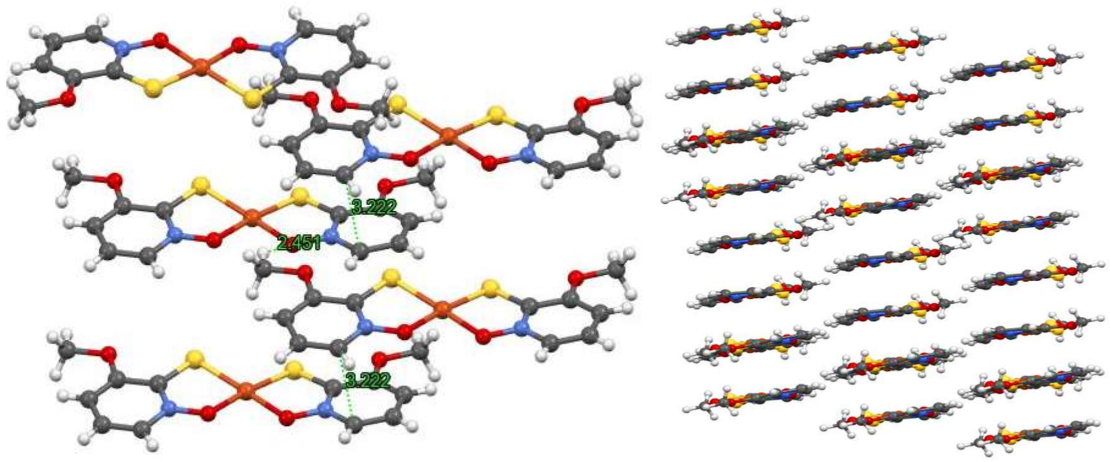

Figure S 25 Crystal packing structure of cis-[Cu(3-OMe-PT)$_2$].

Data for Complex *cis-trans*-[Cu(3-OMe-PT)2]

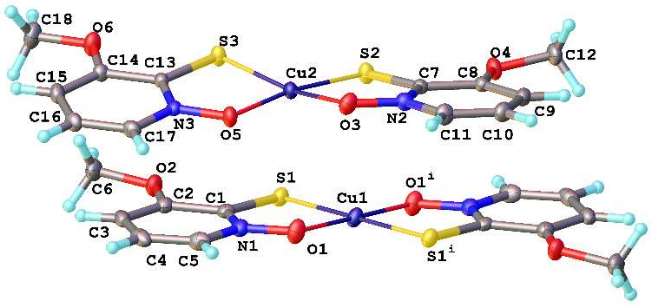

Table S 8 Crystal data and structure refinement for mixed cis-trans-[Cu(3-OMe-PT)2]

Empirical formula	$C_{12}H_{12}CuN_2O_4S_2$
Formula weight	375.90
Temperature/K	120.0
Crystal system	orthorhombic
Space group	Pbca
a/Å	16.8078(5)
b/Å	13.2442(4)
c/Å	18.6661(5)
α/°	90
β/°	90
γ/°	90
Volume/Å^3	4155.2(2)
Z	12
ρ_{calc}g/cm^3	1.803
μ/mm^{-1}	1.894
F(000)	2292.0
Crystal size/mm^3	0.09 × 0.08 × 0.001
Radiation	MoKα (λ = 0.71073)
2Θ range for data collection/°	4.364 to 59.998
Index ranges	$-23 \leq h \leq 23$, $-18 \leq k \leq 18$, $-26 \leq l \leq 26$
Reflections collected	96553
Independent reflections	6056 [R_{int} = 0.0716, R_{sigma} = 0.0284]
Data/restraints/parameters	6056/0/289

Goodness-of-fit on F^2	1.123
Final R indexes [I>=2σ (I)]	R_1 = 0.0438, wR_2 = 0.0854
Final R indexes [all data]	R_1 = 0.0538, wR_2 = 0.0887
Largest diff. peak/hole / e $Å^{-3}$	0.86/-0.41

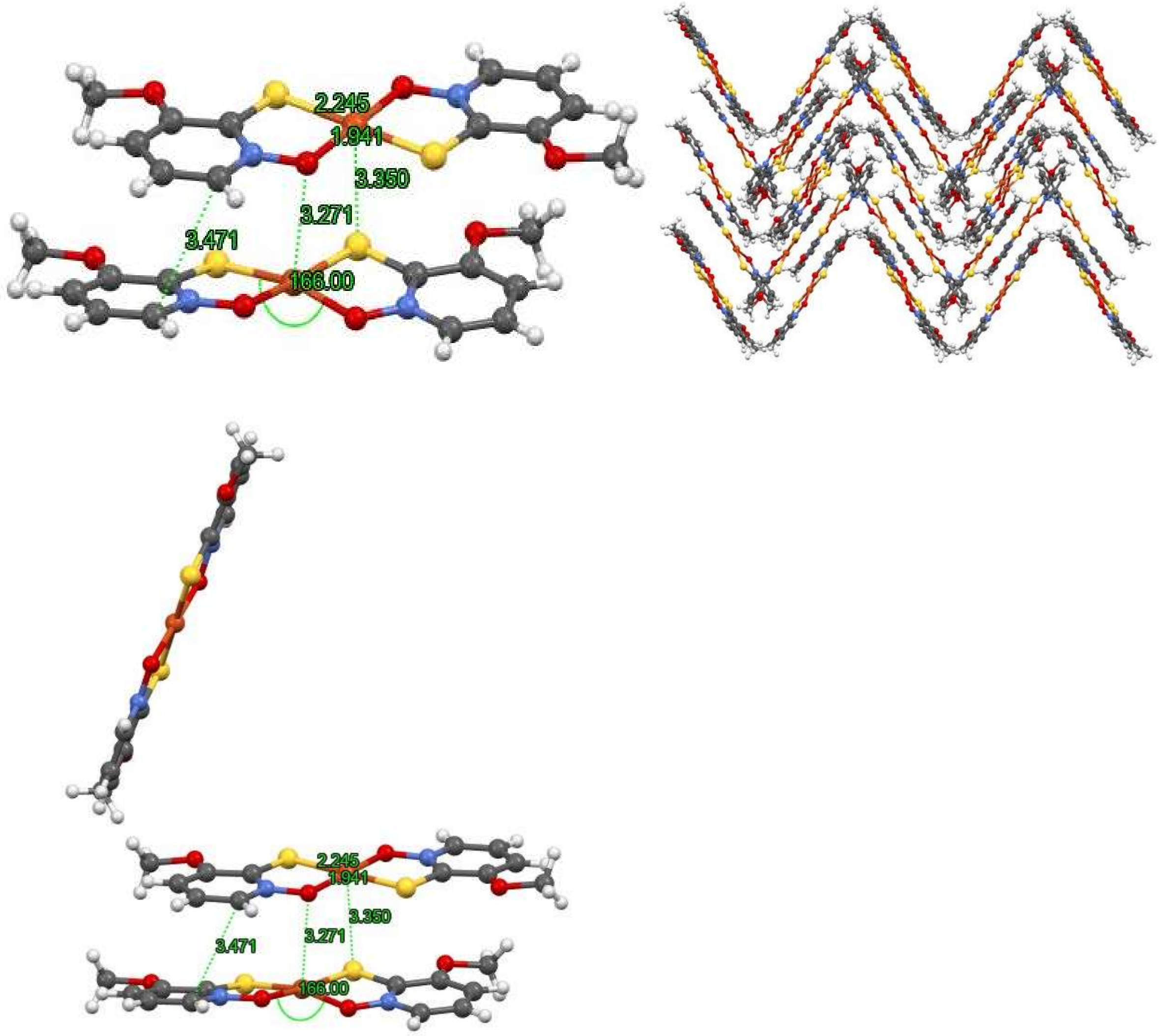

Figure S 26 Crystal packing structure of mixed cis-trans-[Cu(3-OMe-PT)$_2$].

NMR Data

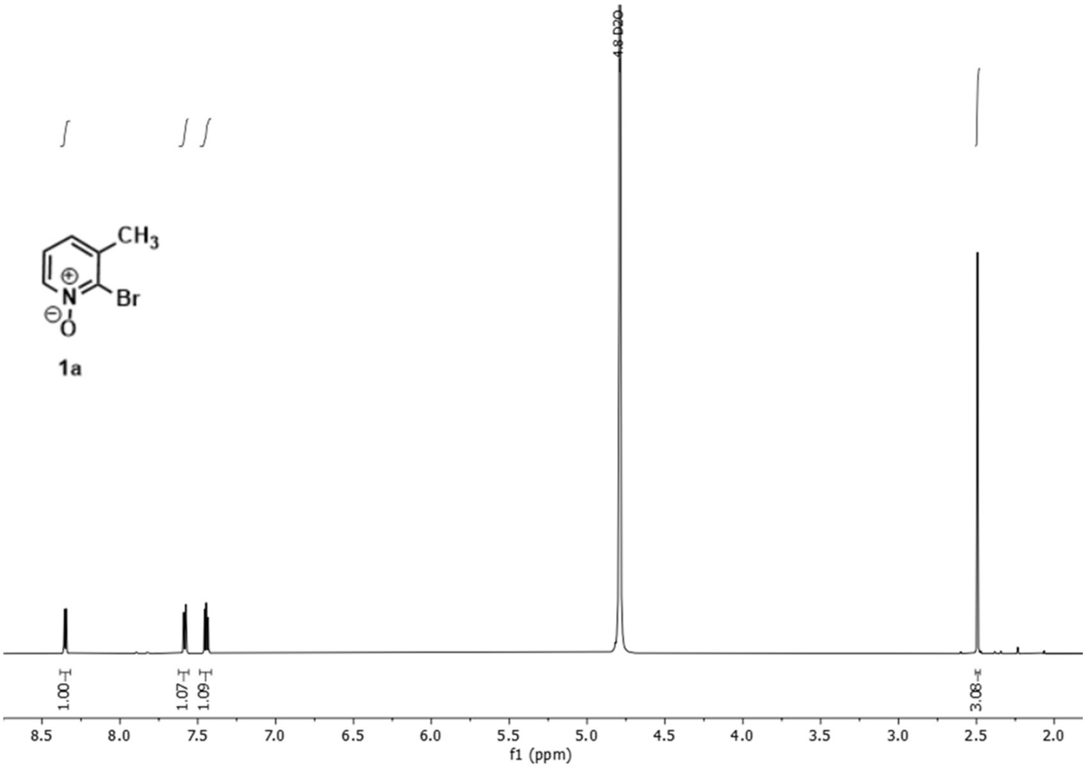

*Figure S 27: ^{1}H NMR spectra of compound **1a** in D_2O (599 MHz) at 295 K*

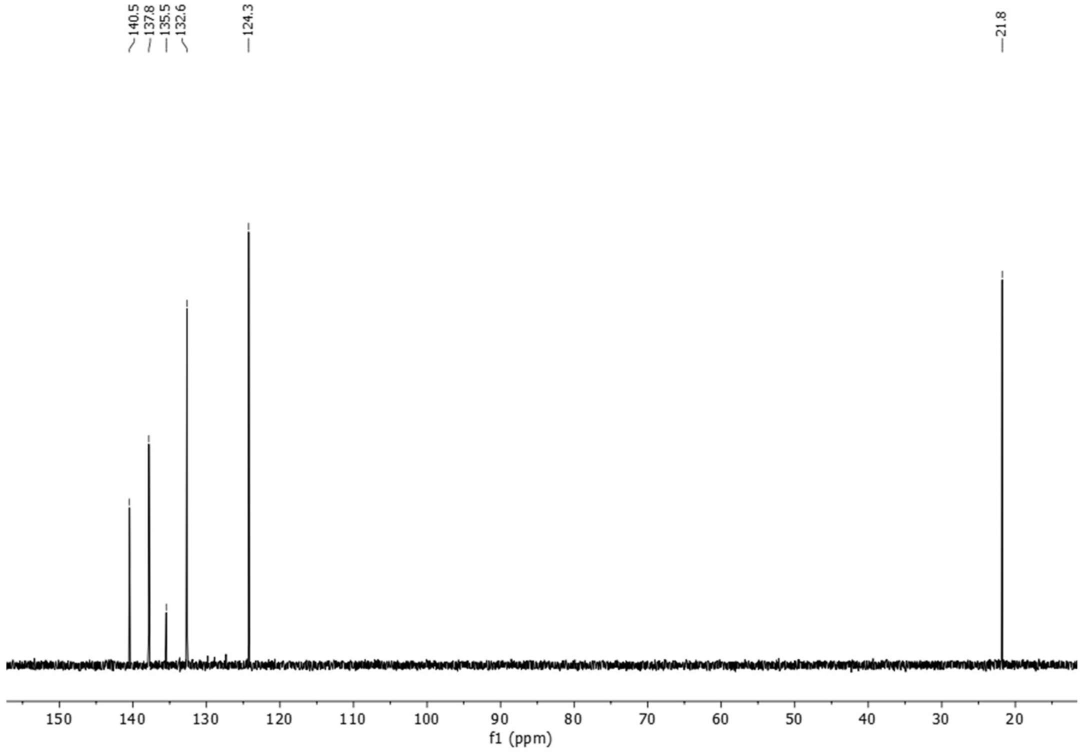

*Figure S 28 $^{13}C\{^1H\}$ NMR spectrum of compound **1a** in D_2O (151 MHz) at 295 K*

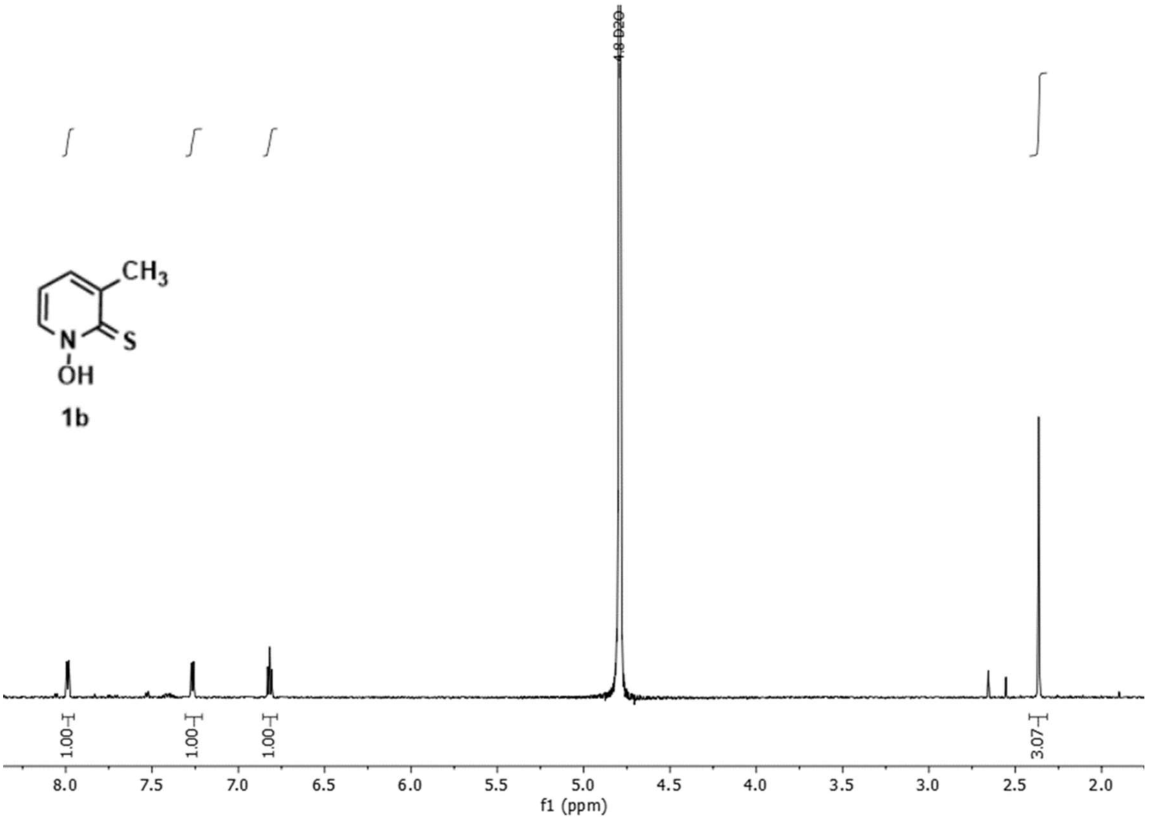

Figure S 29: ^{1}H *NMR spectrum of compound **1b** in* D_2O *(599 MHz) at 295 K*

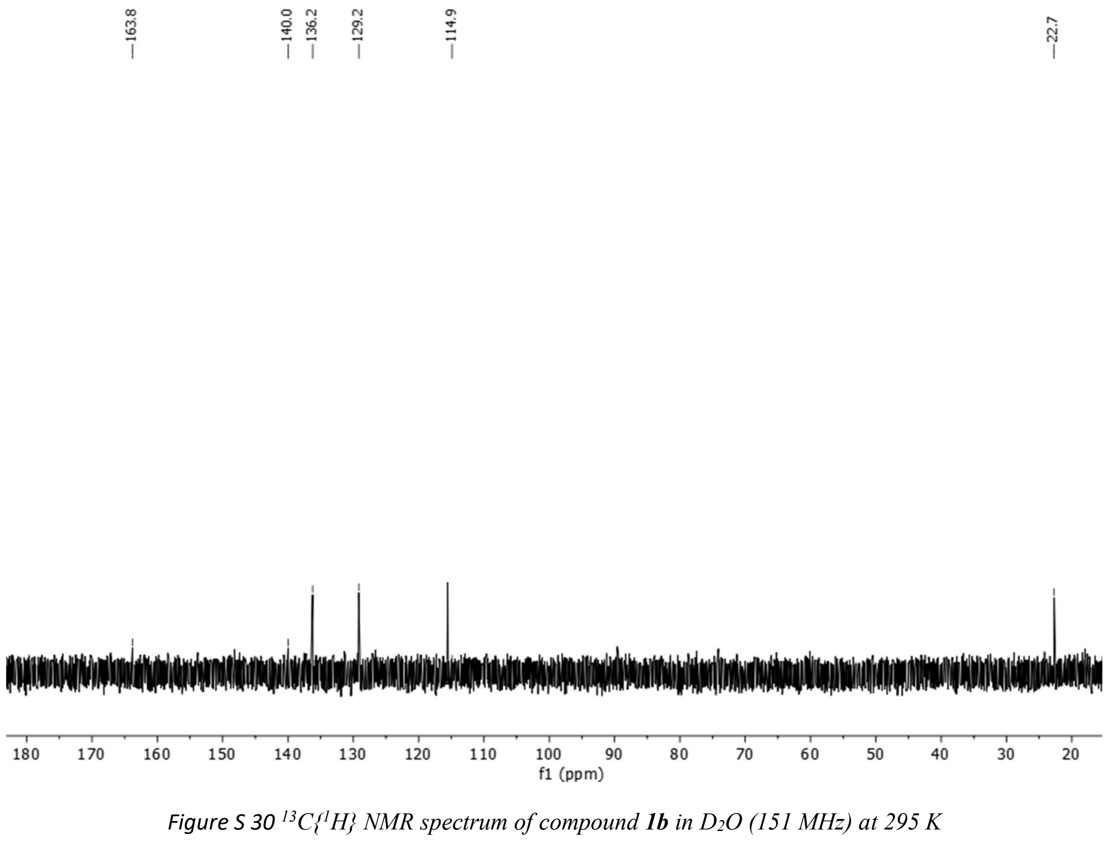

Figure S 30 $^{13}C\{^{1}H\}$ *NMR spectrum of compound **1b** in* D_2O *(151 MHz) at 295 K*

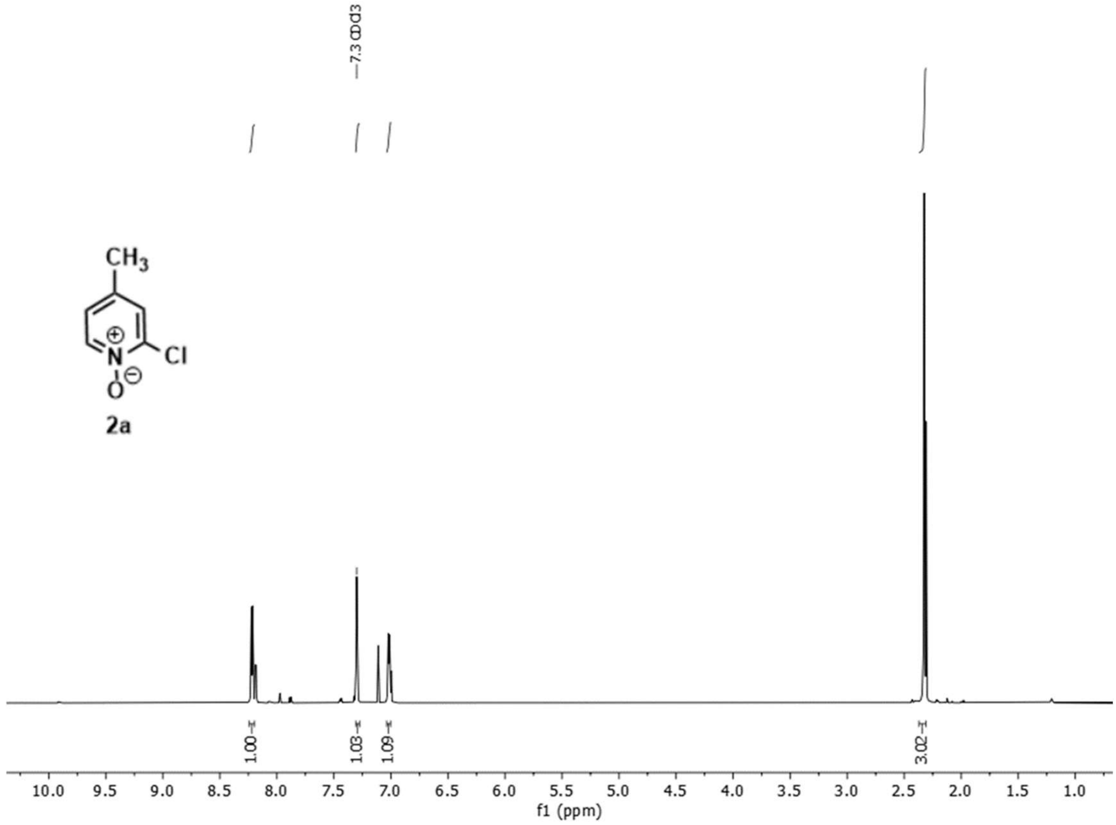

Figure S 31: *^{1}H NMR spectrum of compound* ***2a*** *in chloroform-d (599 MHz) at 295 K*

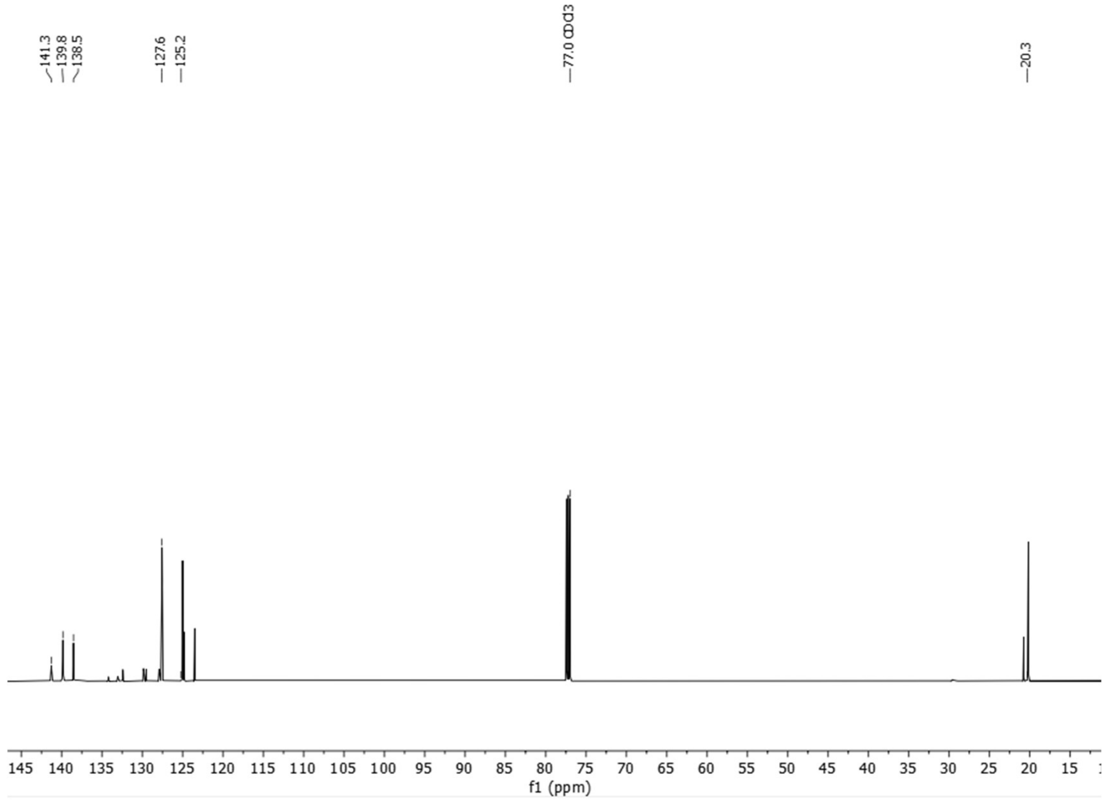

Figure S 32: *^{13}C{^{1}H} NMR spectrum of compound* ***2a*** *in chloroform-d (151 MHz) at 295 K*

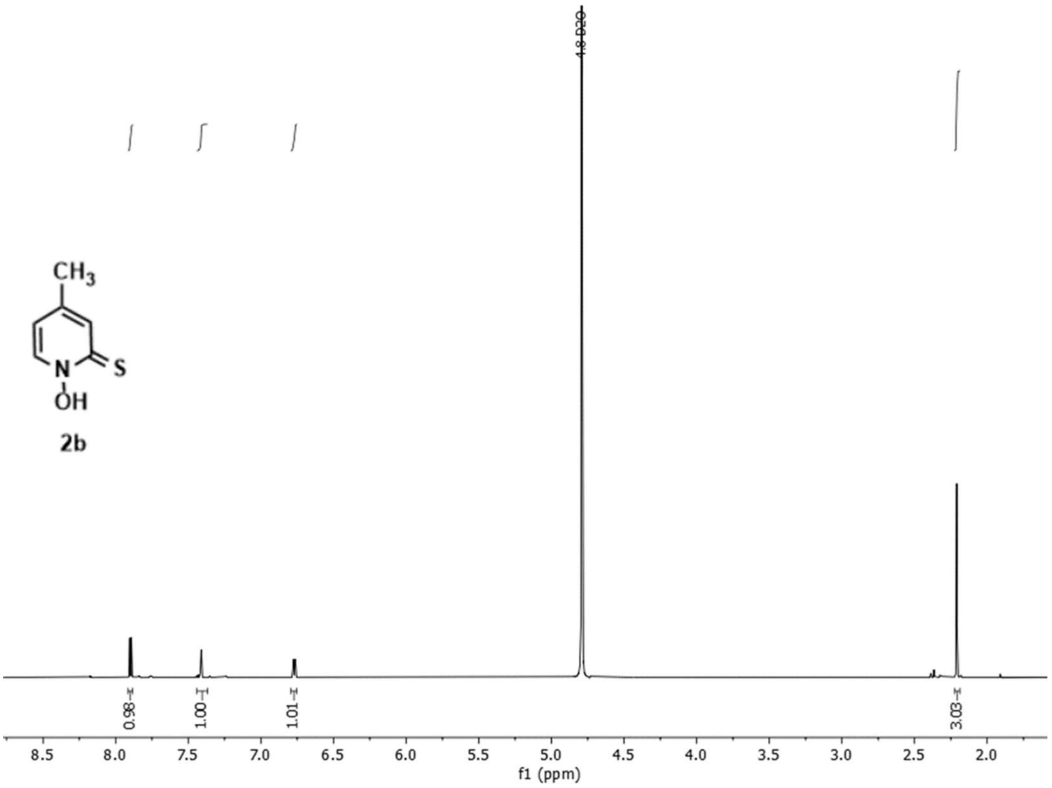

Figure S 33: ^{1}H *NMR spectrum of compound* ***2b*** *in* D_2O *(599 MHz) at 295 K*

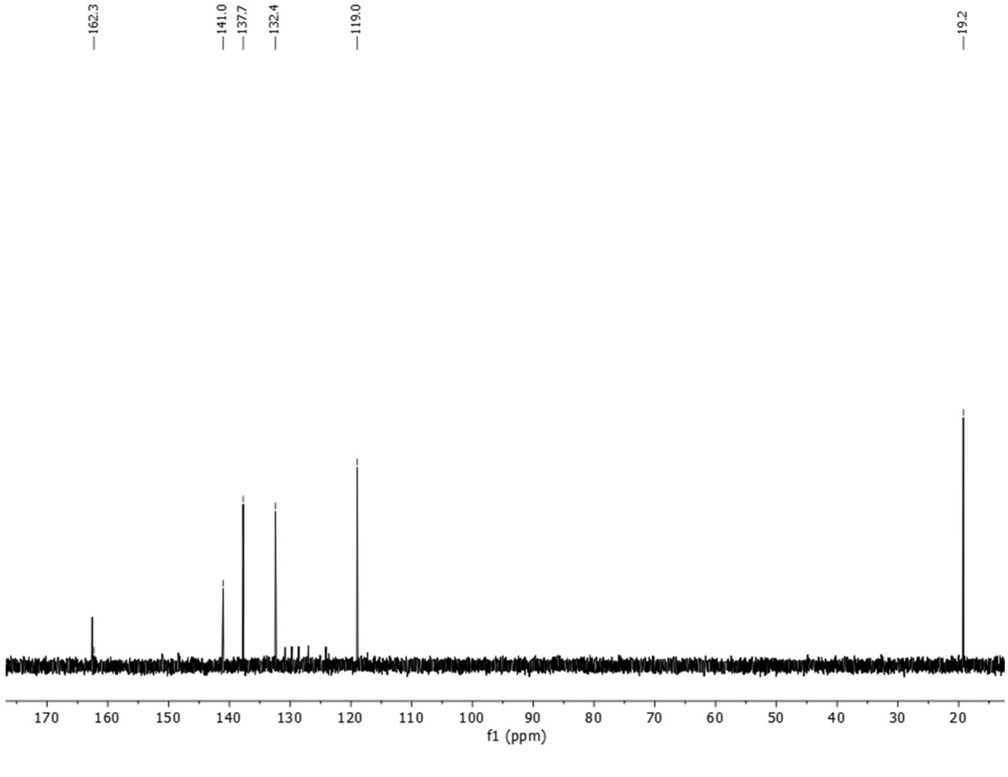

Figure S 34 $^{13}C\{^{1}H\}$ *NMR spectrum of compound* ***2b*** *in* D_2O *(151 MHz) at 295 K*

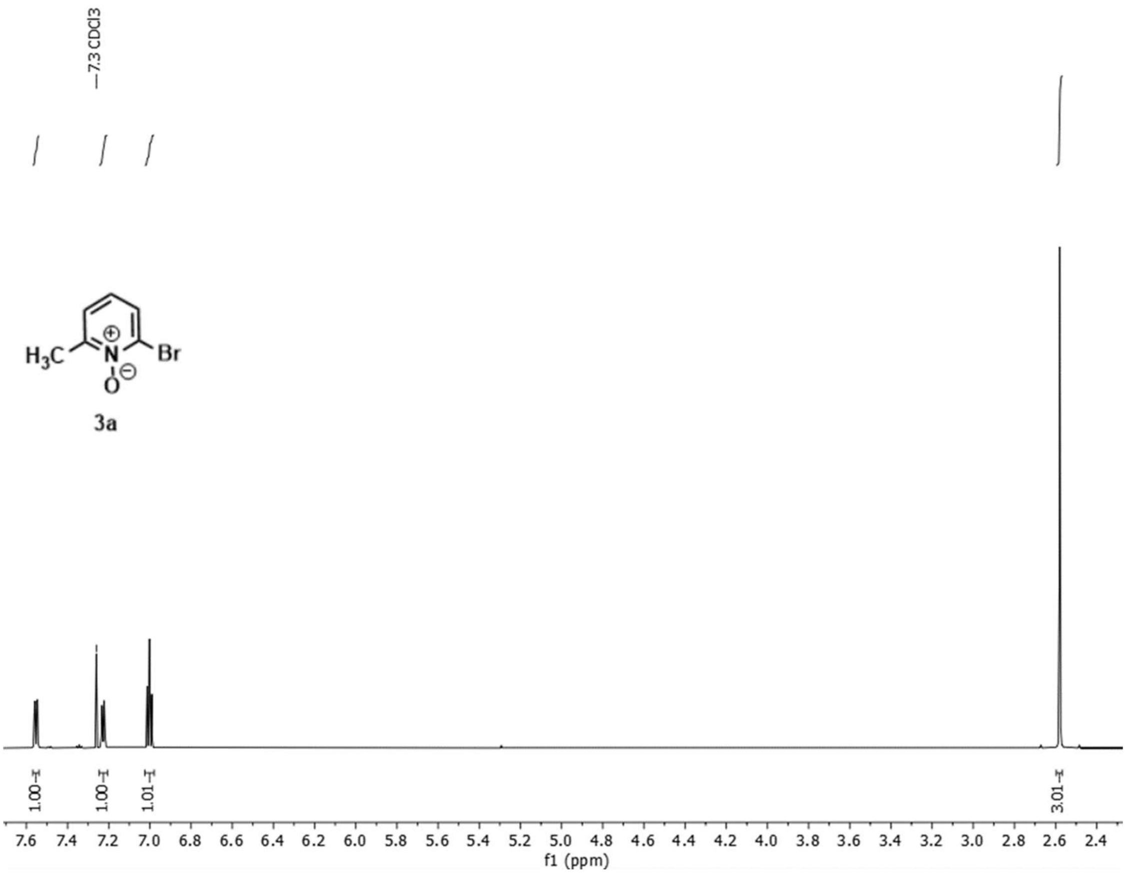

Figure S 35: ^1H *NMR spectrum of compound* ***3a*** *in chloroform-d (599 MHz) at 295 K*

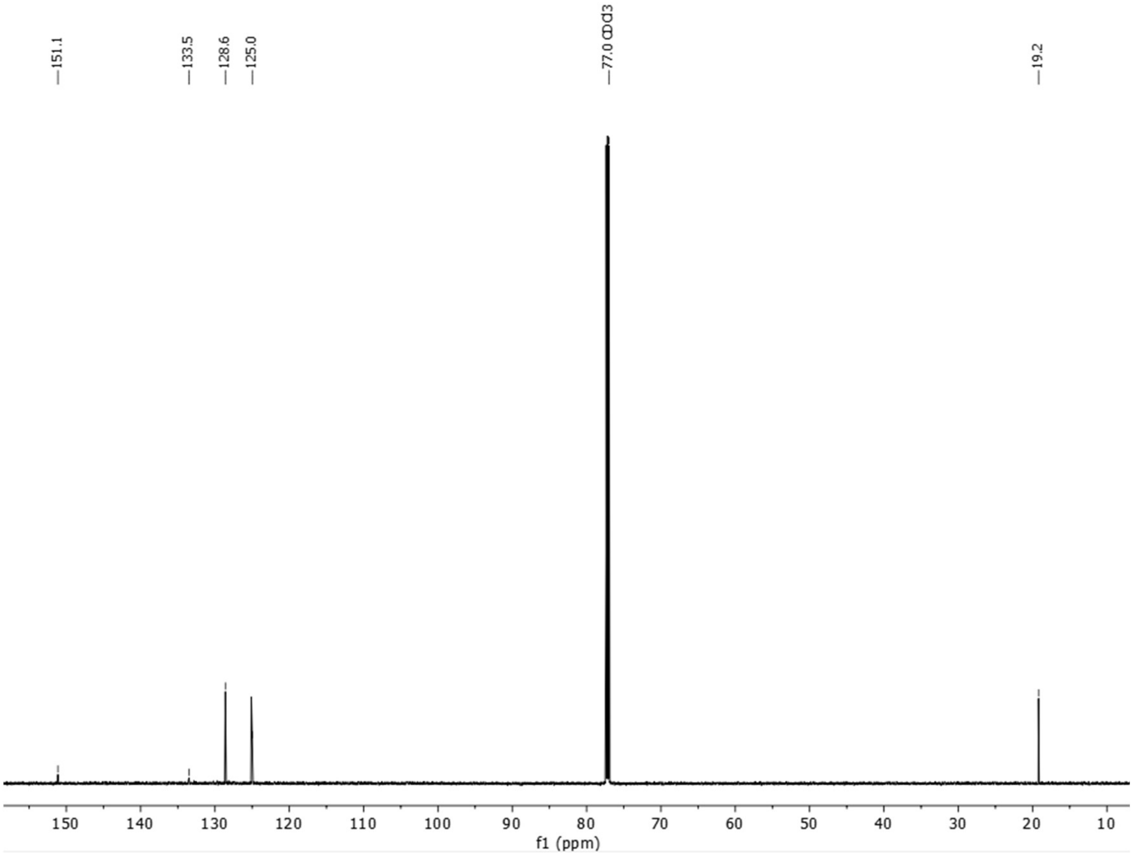

Figure S 36: $^{13}C\{^1H\}$ *NMR spectrum of compound* ***3a*** *in chloroform-d (151 MHz) at 295 K*

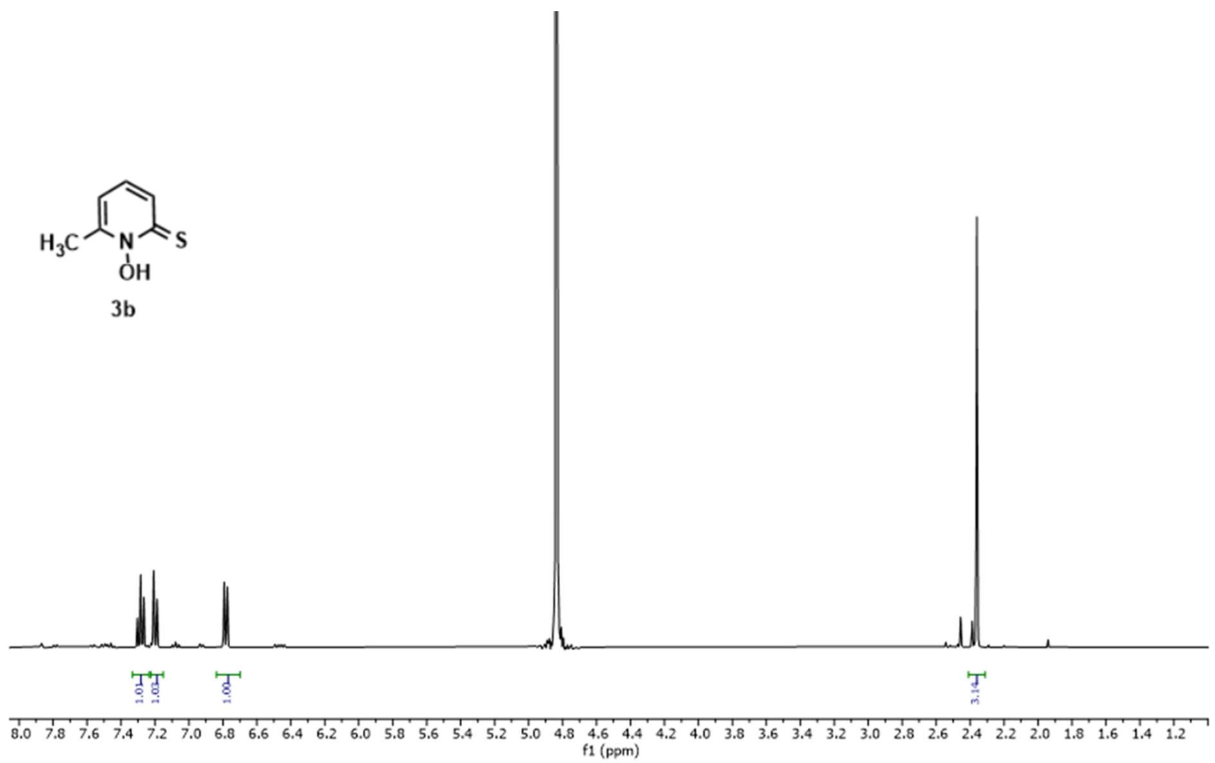

Figure S 37 [1]*H NMR spectrum of compound* ***3b*** *in* D_2O *(599 MHz) at 295 K*

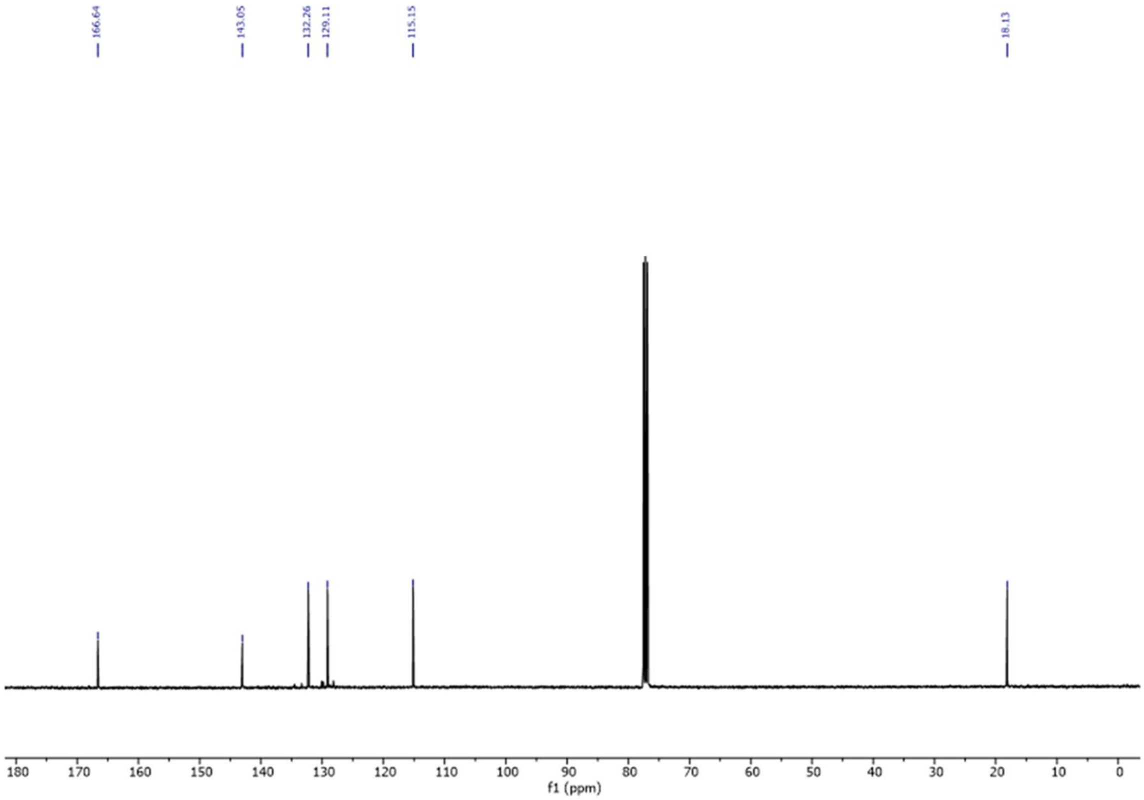

Figure S 38 $^{13}C\{^1H\}$ *NMR spectrum of compound* ***3b*** *in* D_2O *(151 MHz) at 295 K*

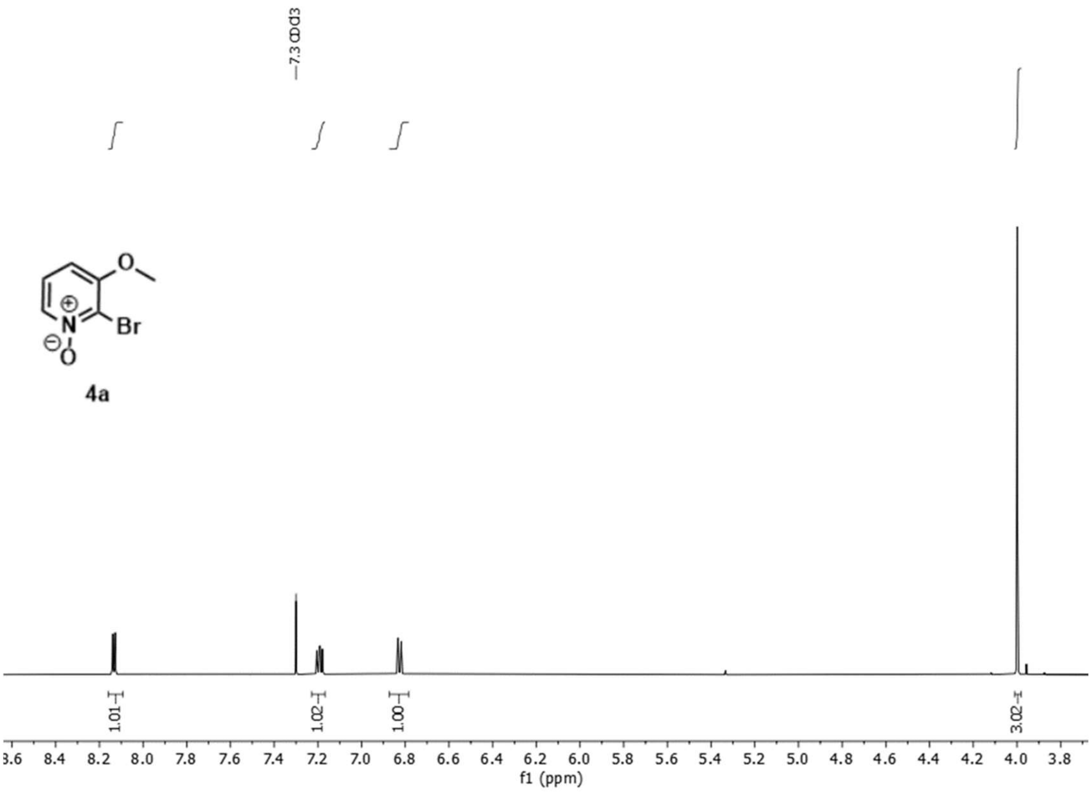

*Figure S 39: ^{1}H NMR spectrum of compound **4a** in chloroform-d (599 MHz) at 295 K*

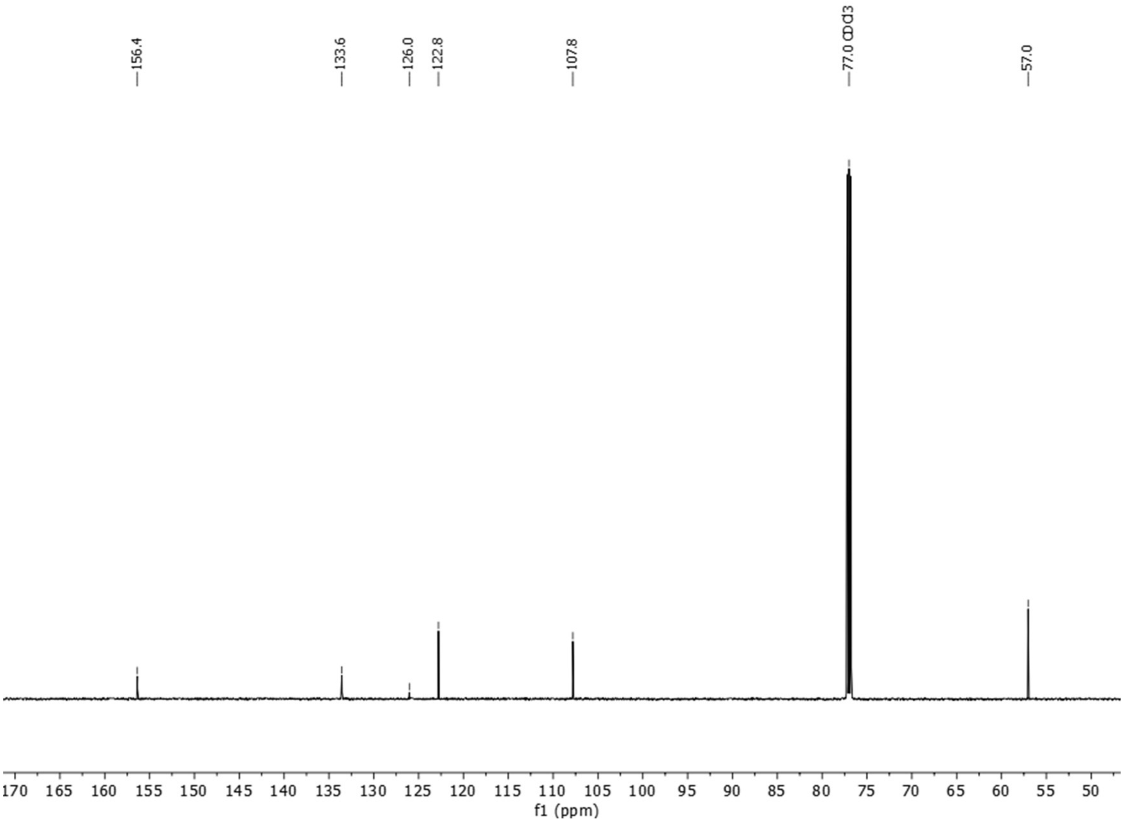

*Figure S 40 $^{13}C\{^{1}H\}$ NMR spectrum of compound **4a** in chloroform-d (151 MHz) at 295 K*

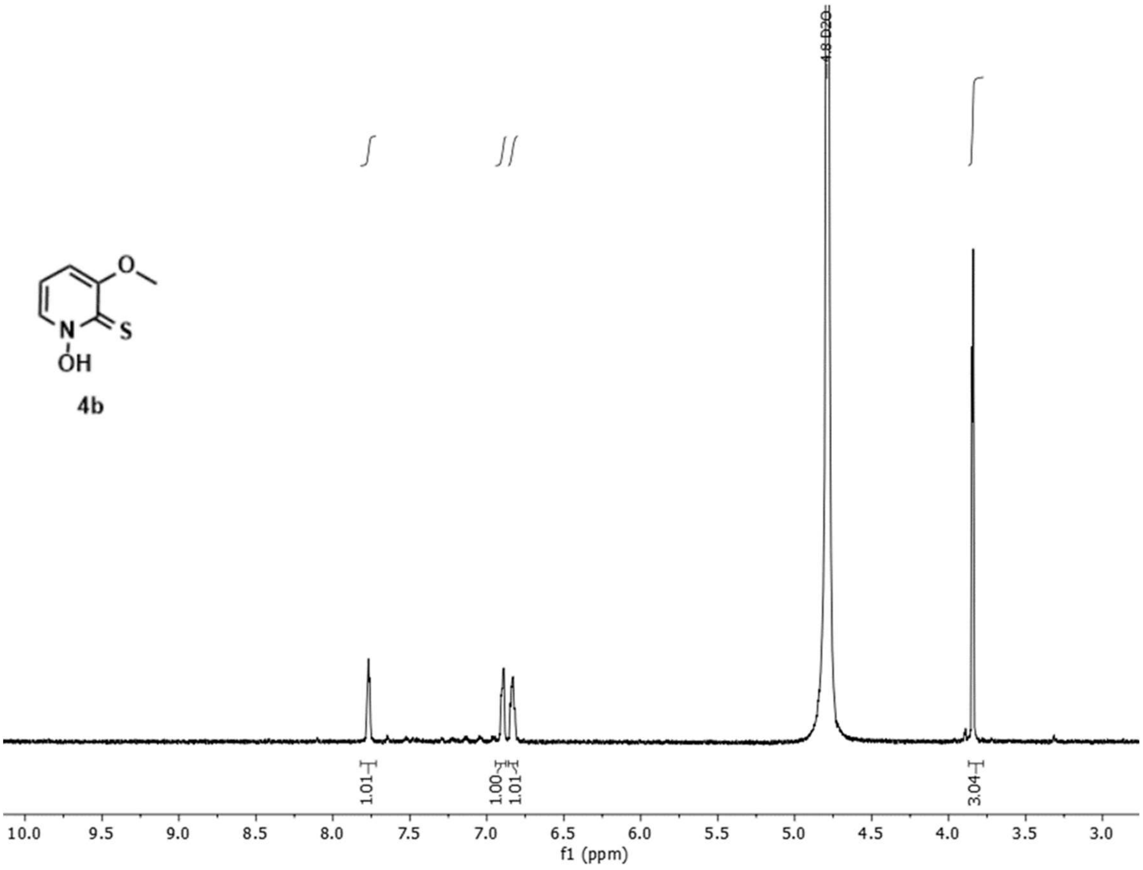

Figure S 41: ^{1}H *NMR spectrum of compound* ***4b*** *in* D_2O *(599 MHz) at 295 K*

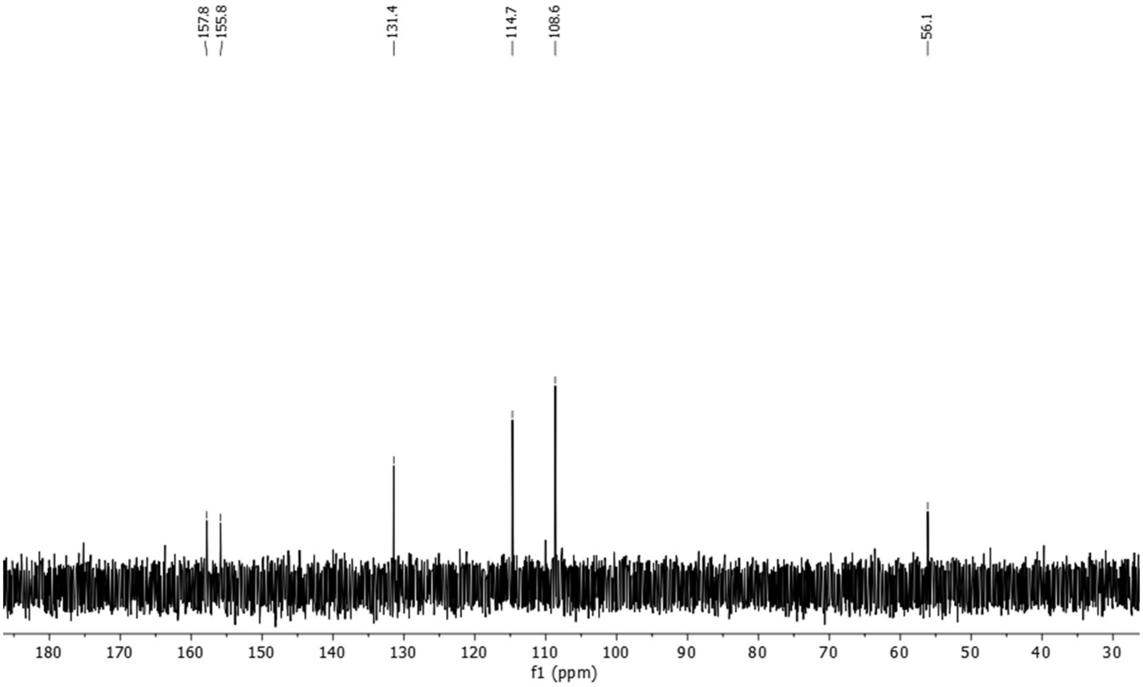

Figure S 42 $^{13}C\{^{1}H\}$ *NMR spectrum of compound* ***4b*** *in* D_2O *(151 MHz) at 295 K*

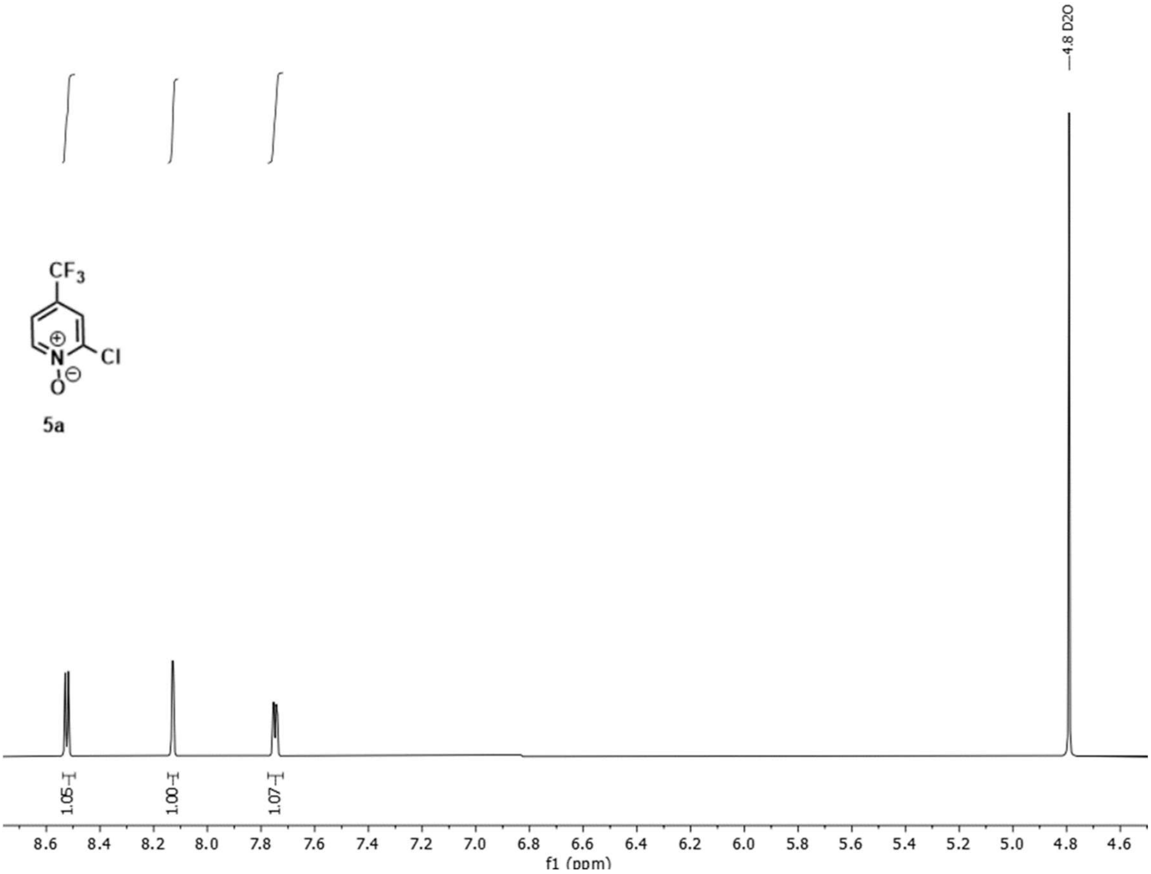

*Figure S 43: ^{1}H NMR spectrum of compound **5a** in D_2O (599 MHz) at 295 K*

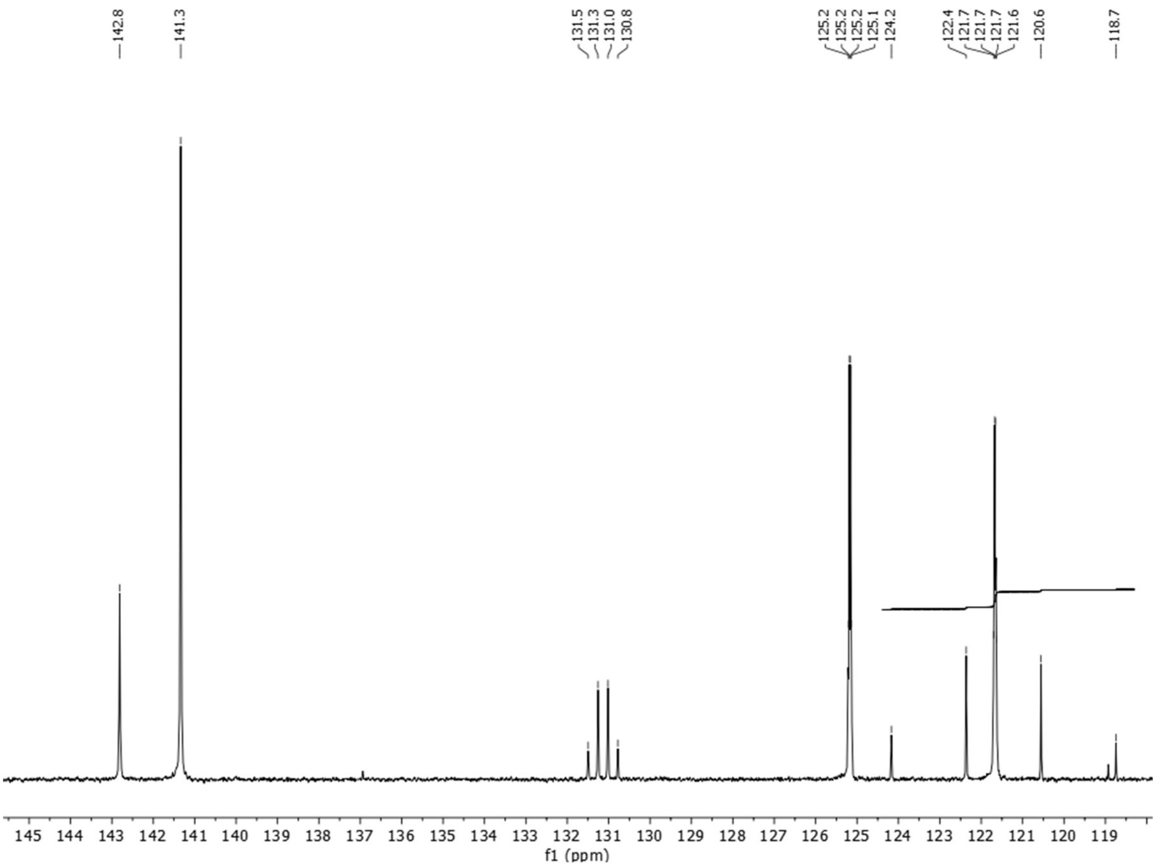

*Figure S 44 $^{13}C\{^{1}H\}$ NMR spectrum of compound **5a** in D_2O (151 MHz) at 295 K*

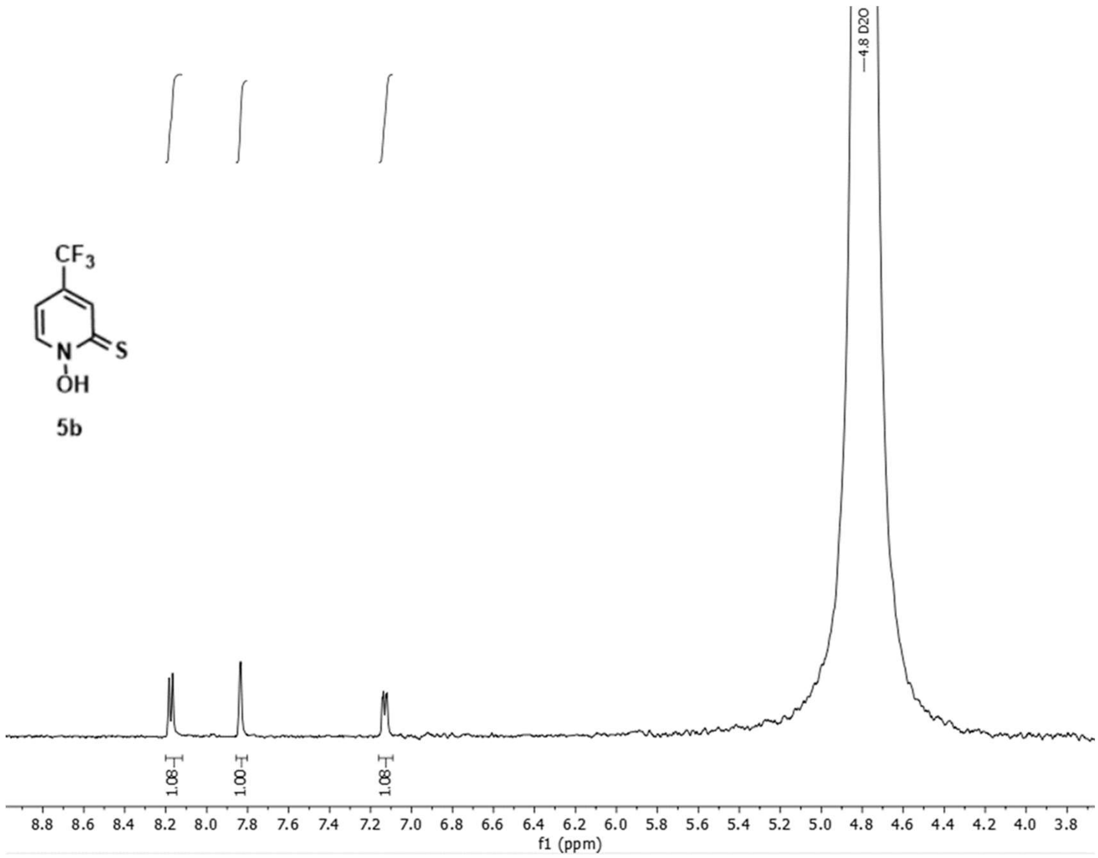

Figure S 45: ^{1}H *NMR spectrum of compound* ***5b*** *in* D_2O *(599 MHz) at 295 K*

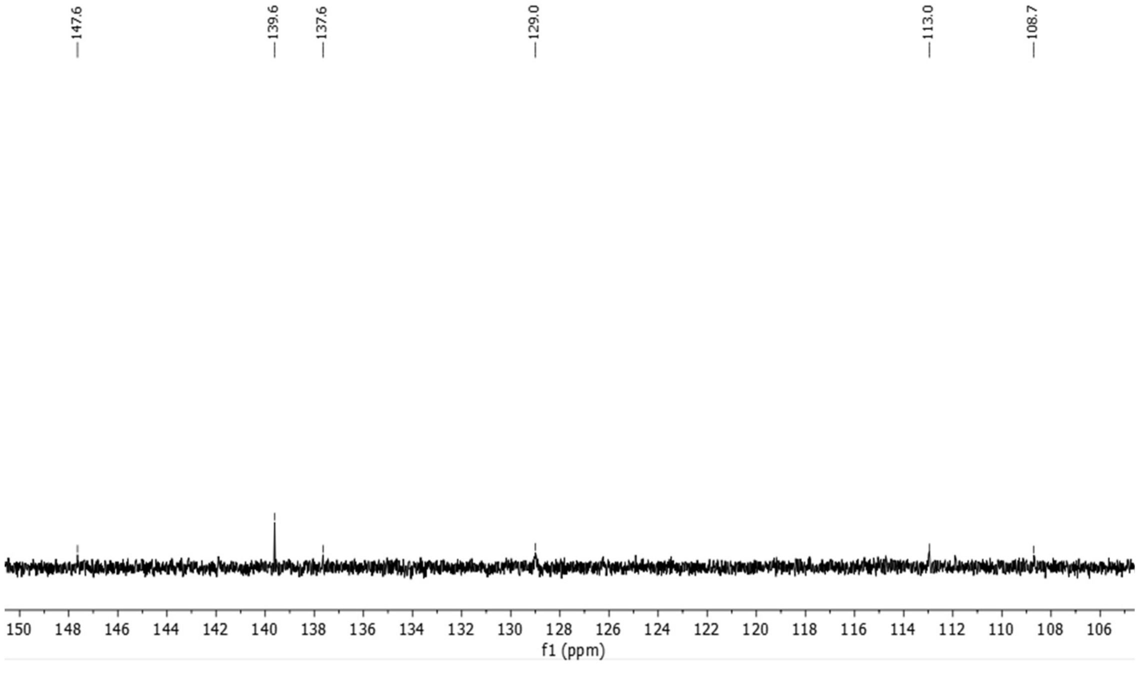

Figure S 46: $^{13}C\{^{1}H\}$ *NMR spectrum of compound* ***5b*** *in* D_2O *(151 MHz) at 295 K*

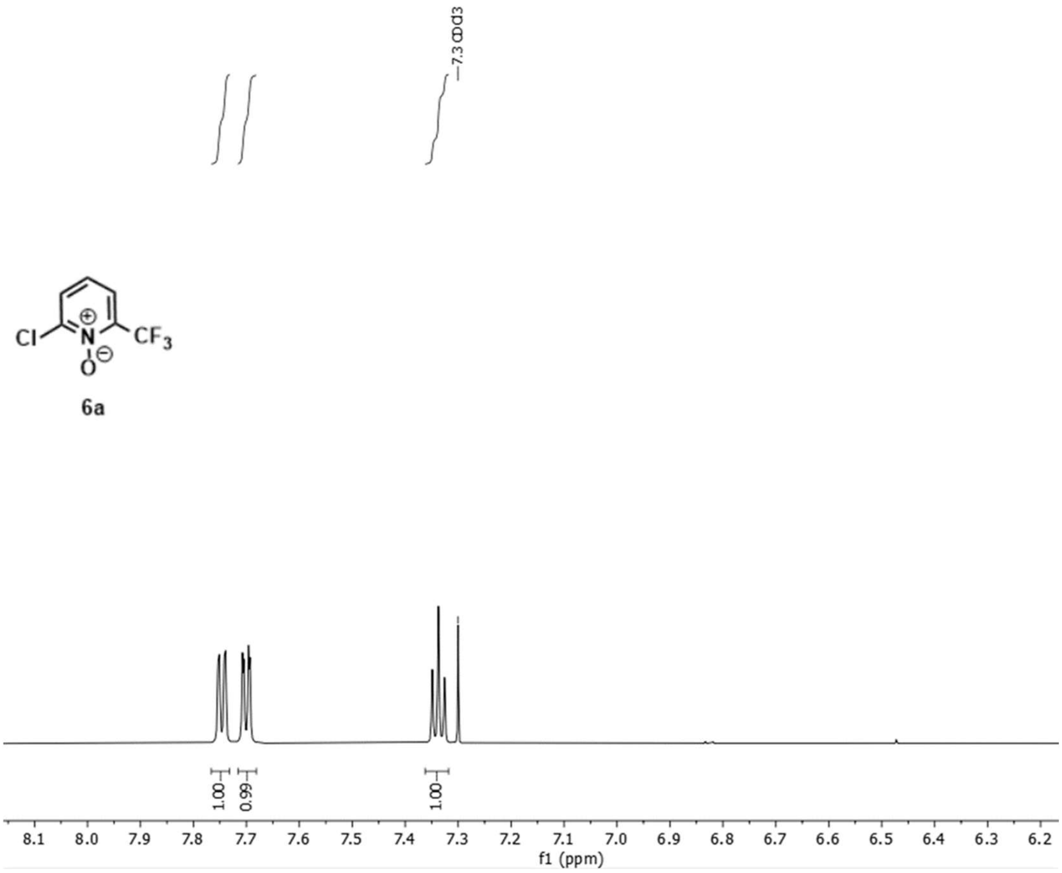

Figure S 47: ^{1}H *NMR spectrum of compound* ***6a*** *in chloroform-d (599 MHz) at 295 K*

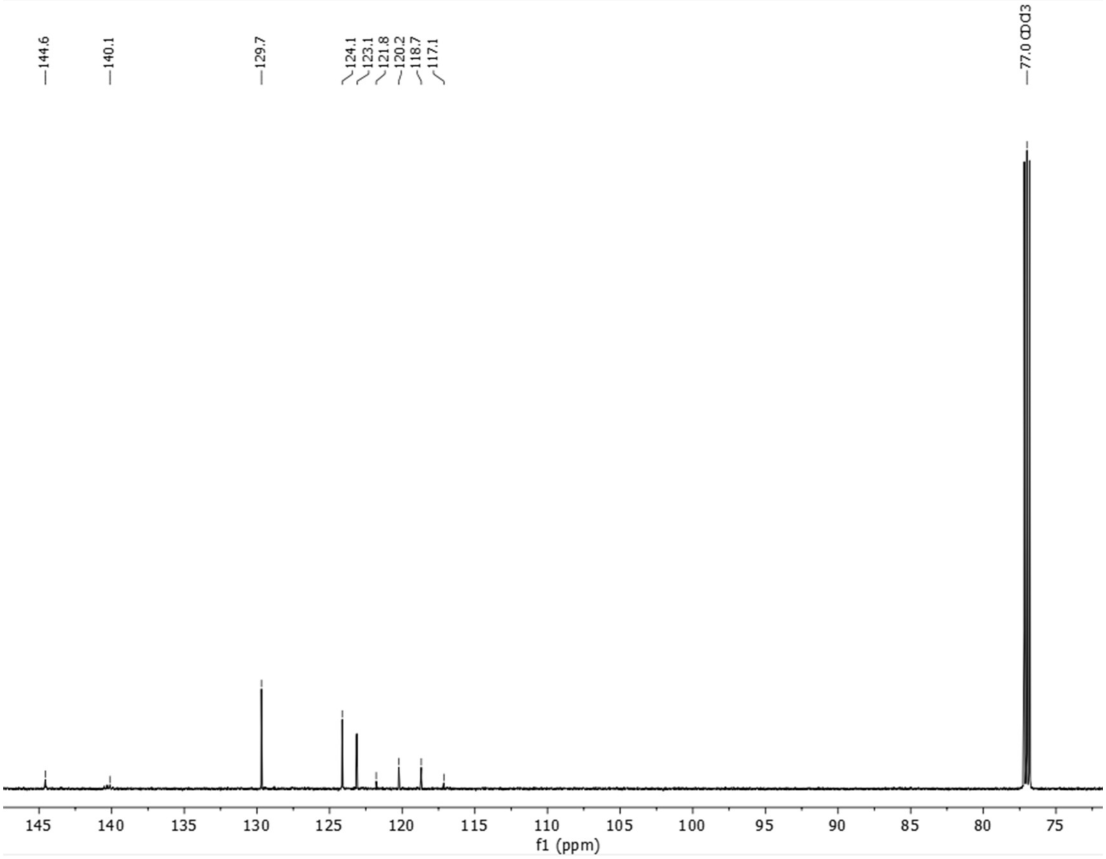

Figure S 48: $^{13}C\{^{1}H\}$ *NMR spectrum of compound* ***6a*** *in chloroform-d (151 MHz) at 295 K*

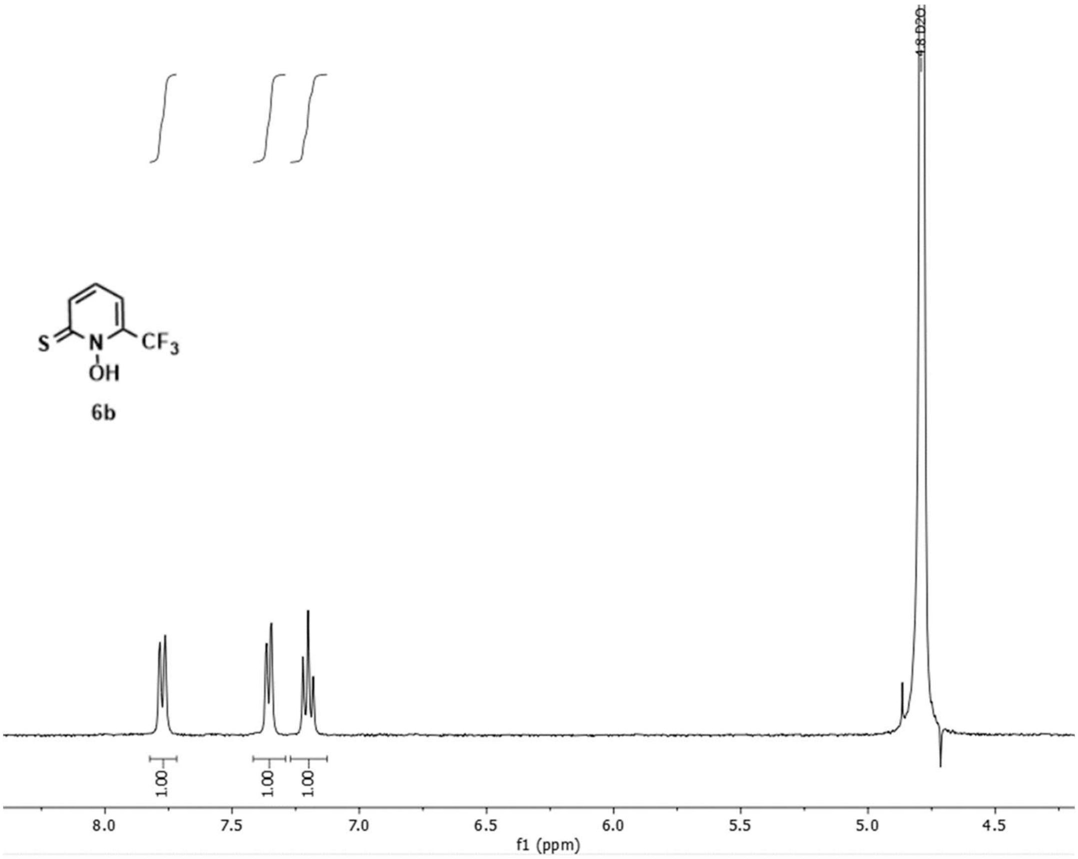

*Figure S 49: ^{1}H NMR spectrum of compound **6b** in D_2O (599 MHz) at 295 K*

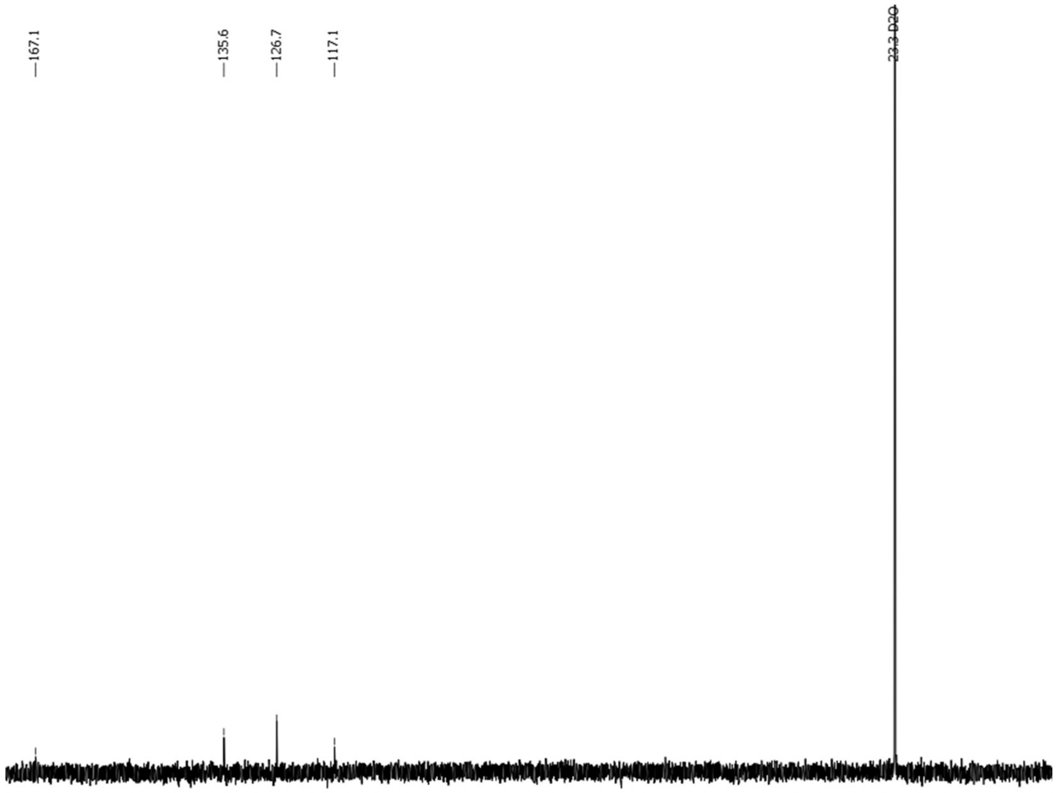

*Figure S 50: $^{13}C\{^{1}H\}$ NMR spectrum of compound **6b** in D_2O (151 MHz) at 295 K*

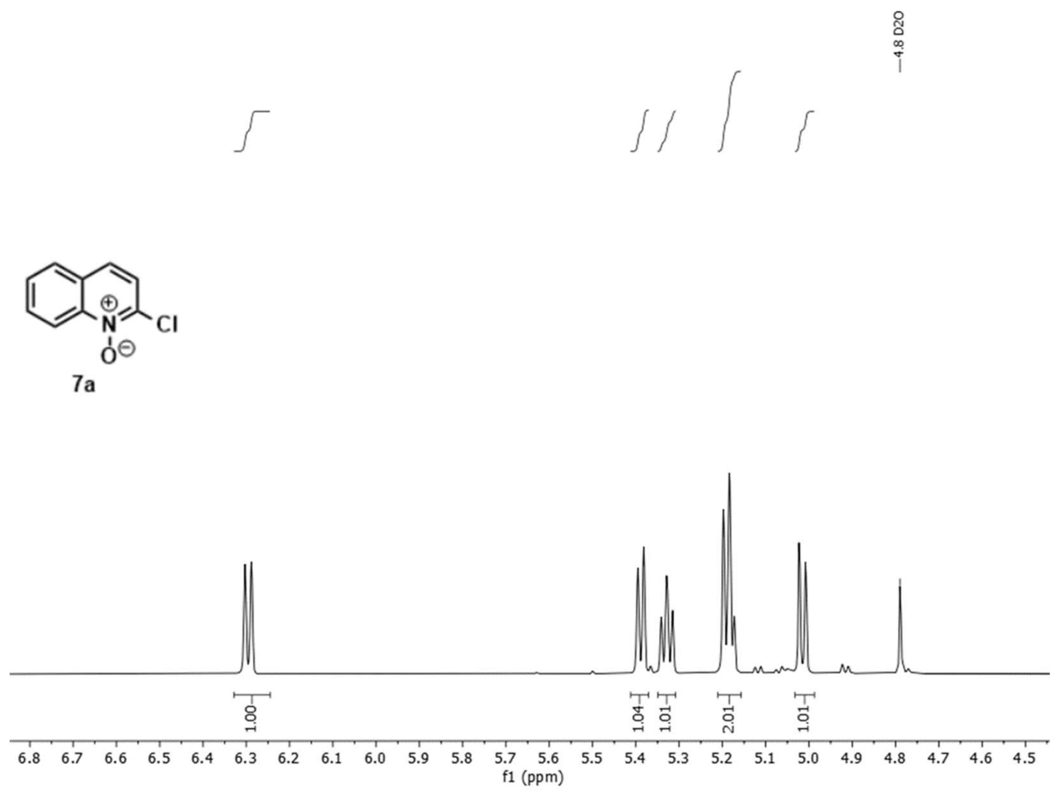

*Figure S 51: ^{1}H NMR spectrum of compound **7a** in D_2O (599 MHz) at 295 K*

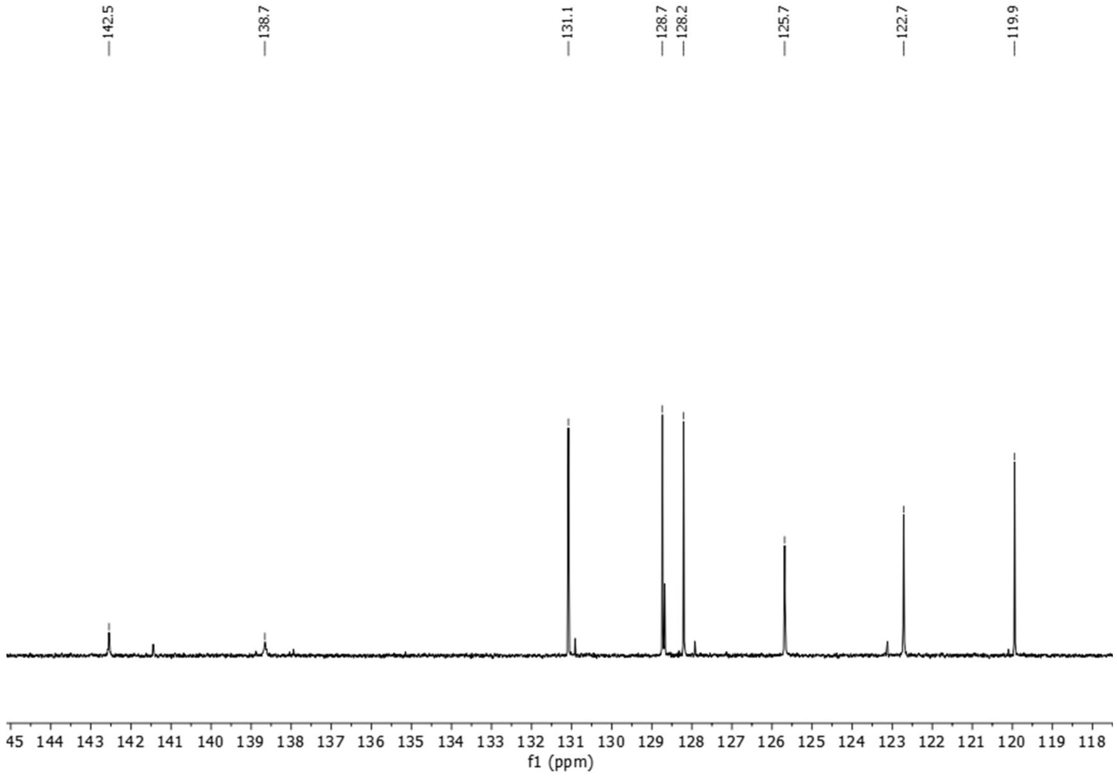

*Figure S 52: $^{13}C\{^{1}H\}$ NMR spectrum of compound **7a** in D_2O (151 MHz) at 295 K*

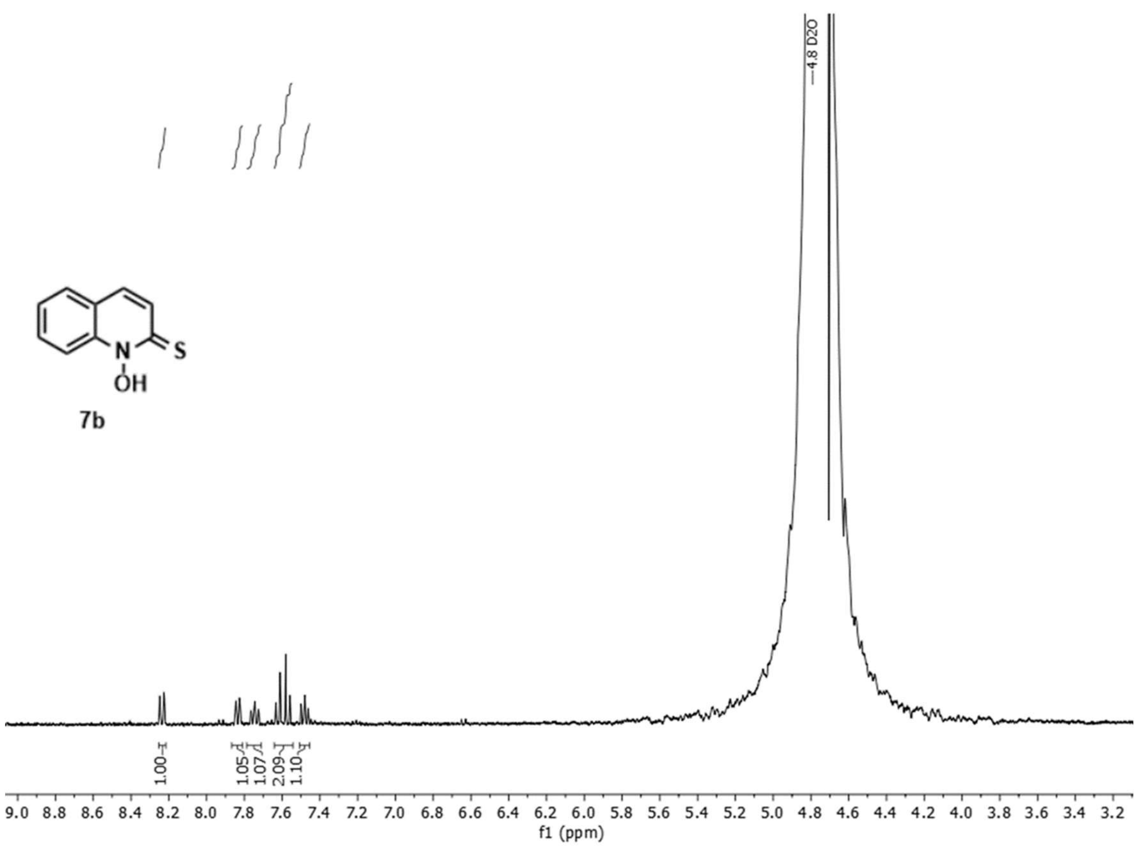

*Figure S 53: ^{1}H NMR spectrum of compound **7b** in D_2O (599 MHz) at 295 K*

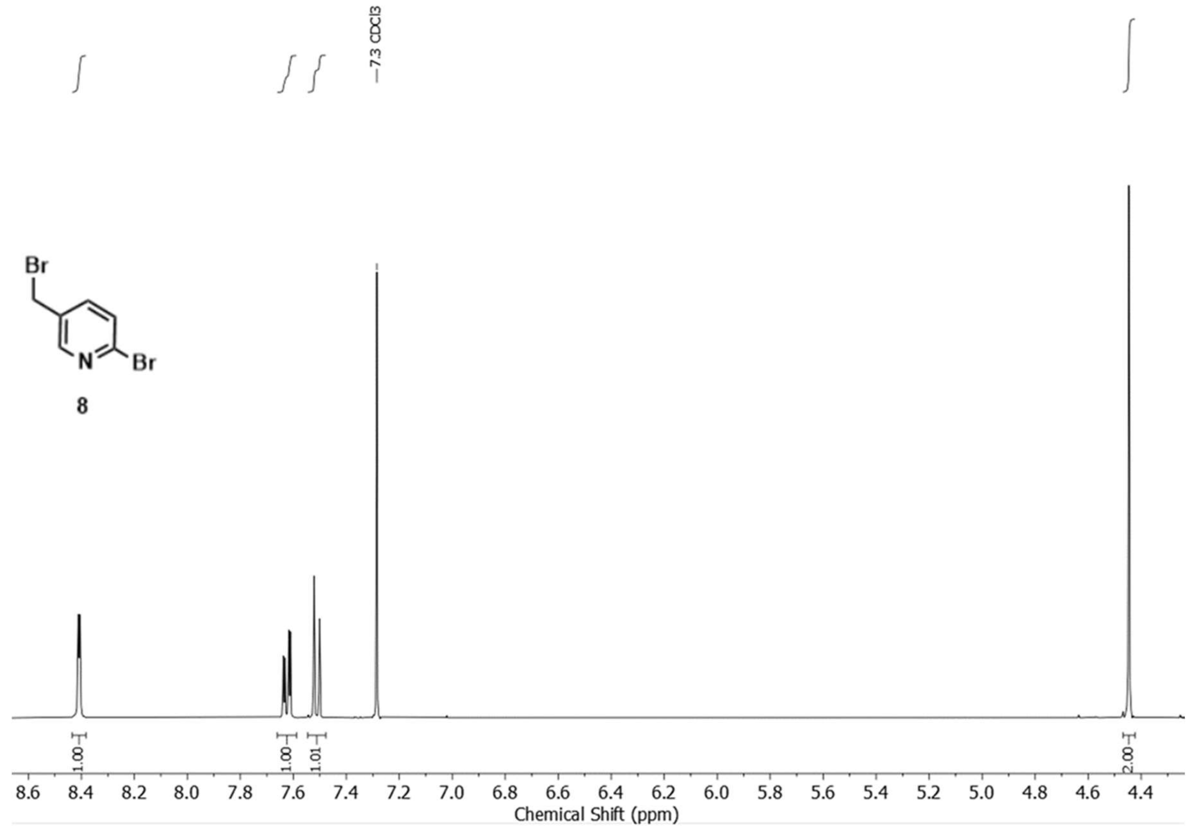

*Figure S 54: ^{1}H NMR spectrum of compound **8** in chloroform-d (599 MHz) at 295 K*

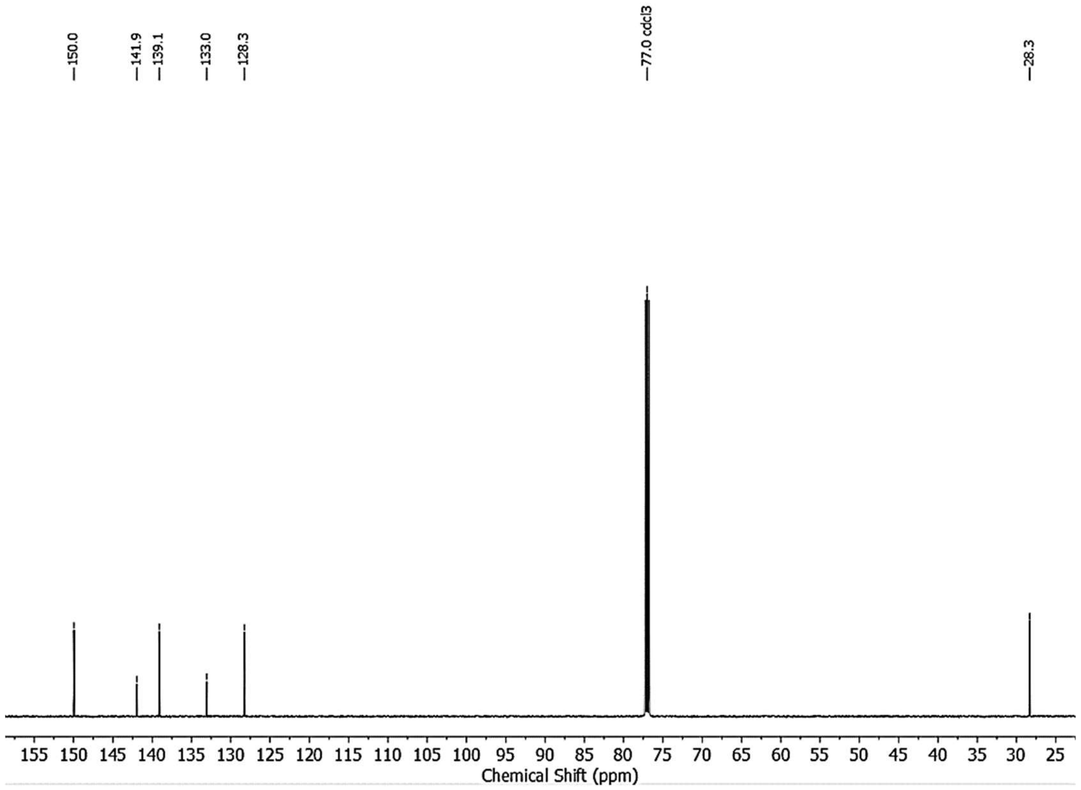

*Figure S 55: $^{13}C\{^{1}H\}$ NMR spectrum of compound **8** in chloroform-d (151 MHz) at 295 K*

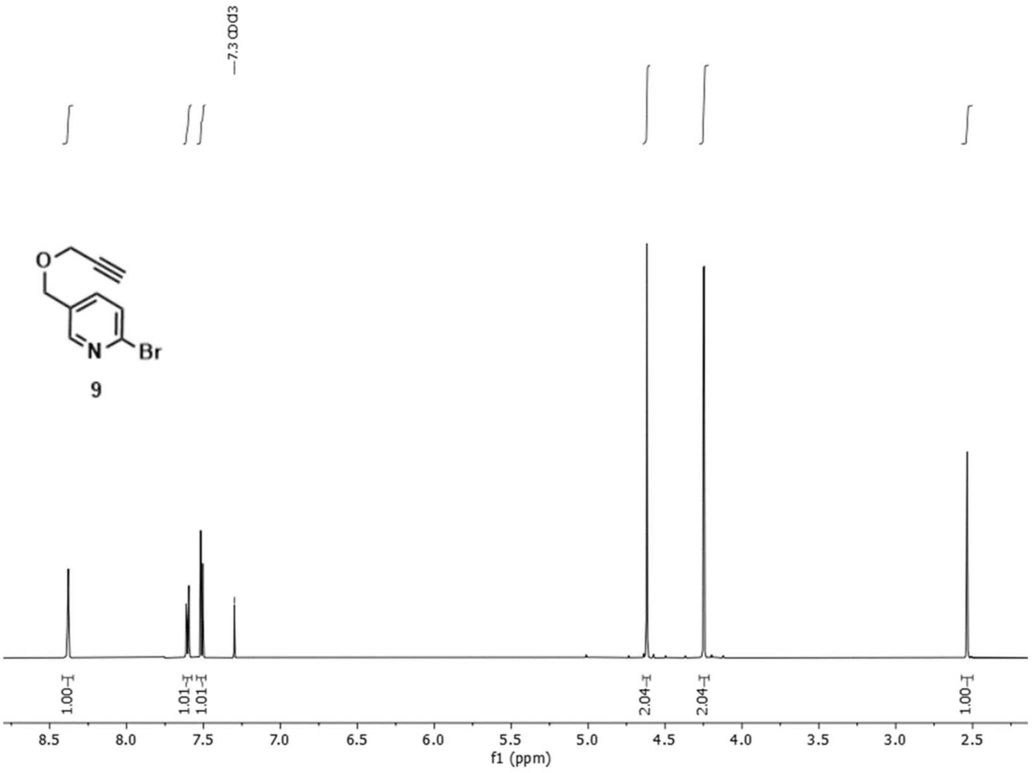

*Figure S 56: ^{1}H NMR spectrum of compound **9** in chloroform-d (599 MHz) at 295 K*

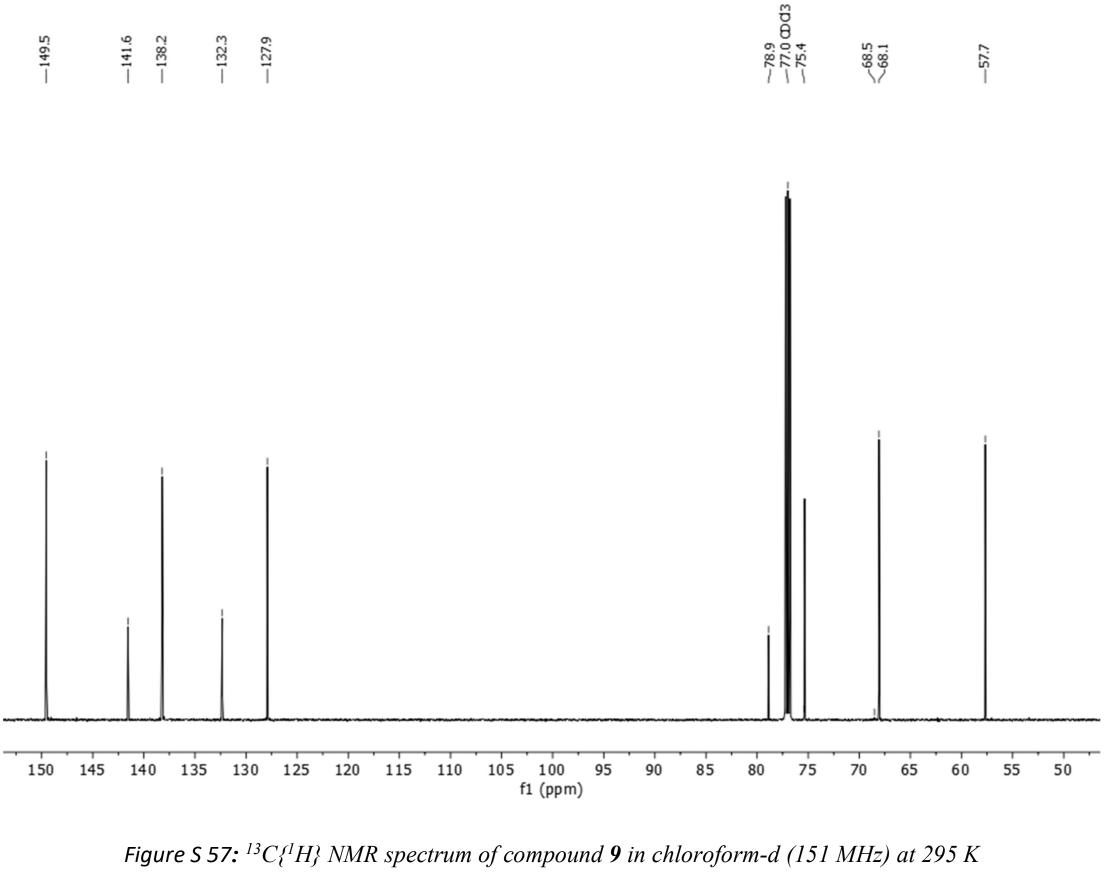

*Figure S 57: $^{13}C\{^{1}H\}$ NMR spectrum of compound **9** in chloroform-d (151 MHz) at 295 K*

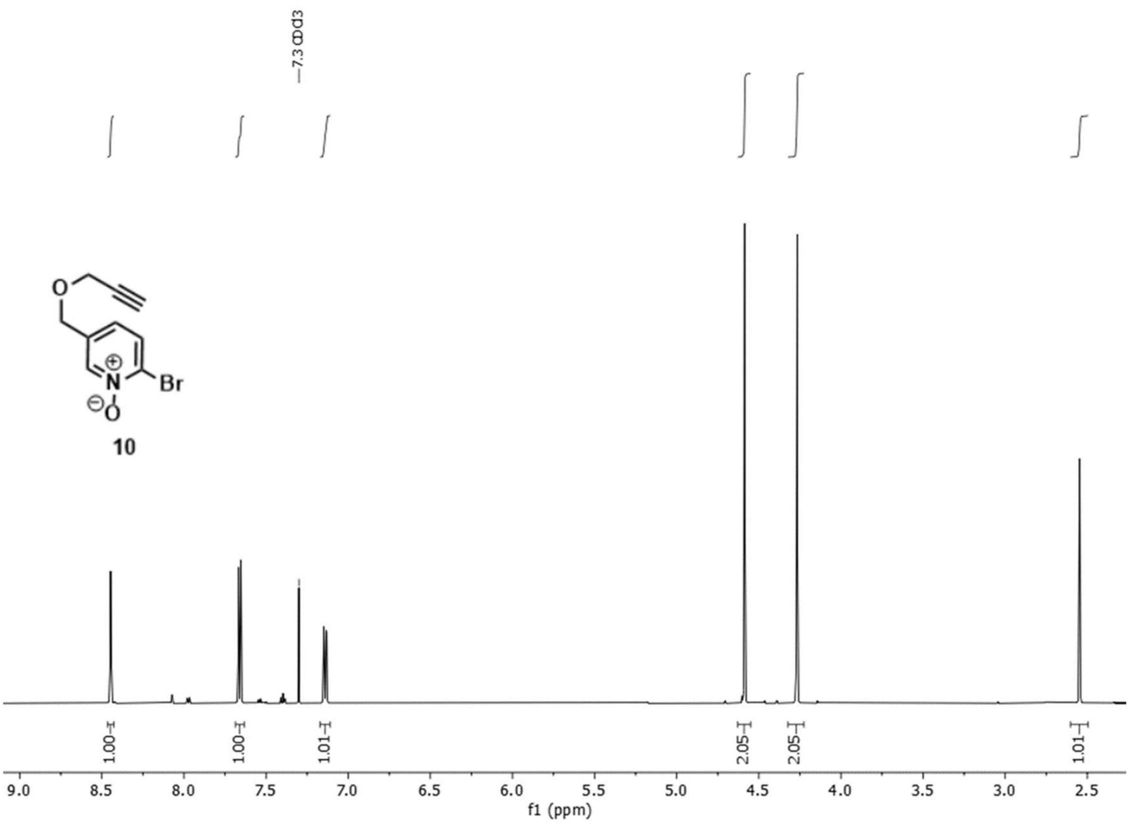

Figure S 58: *^{1}H NMR spectrum of compound* ***10*** *in chloroform-d (599 MHz) at 295 K*

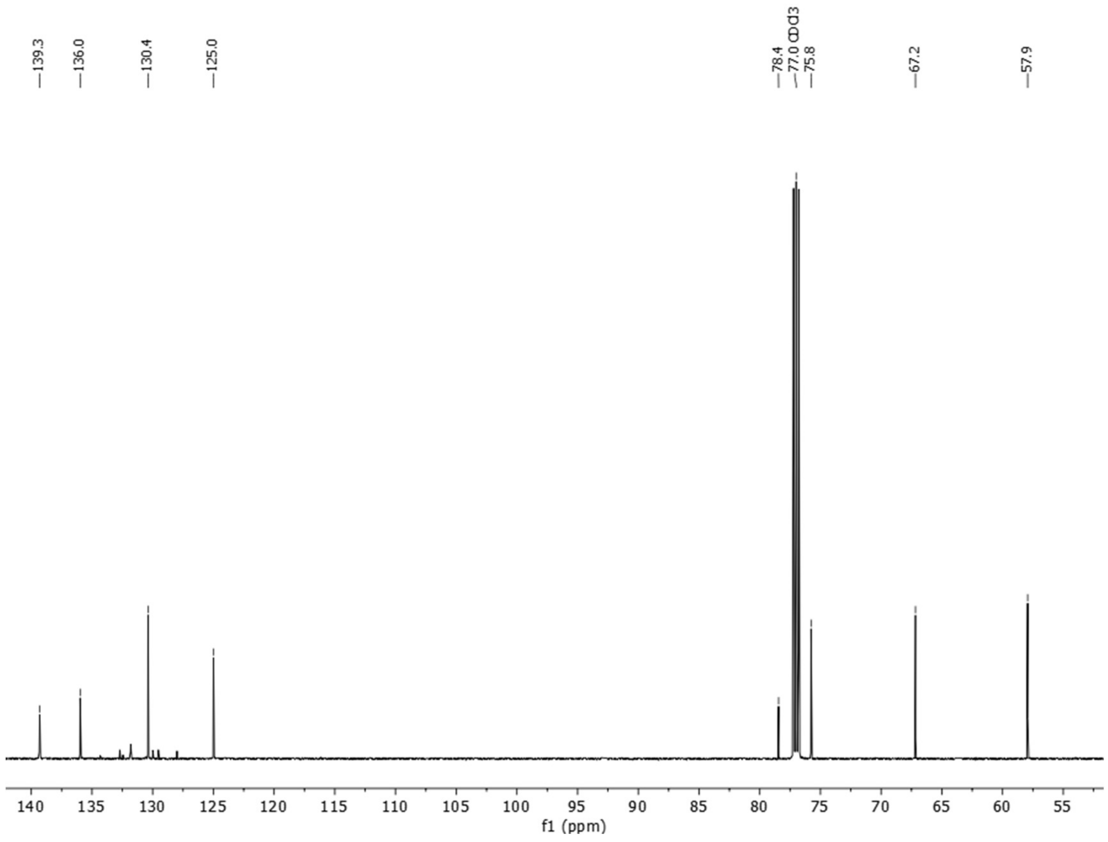

Figure S 59: *^{13}C{^{1}H} NMR spectrum of compound* ***10*** *in chloroform-d (151 MHz) at 295 K*

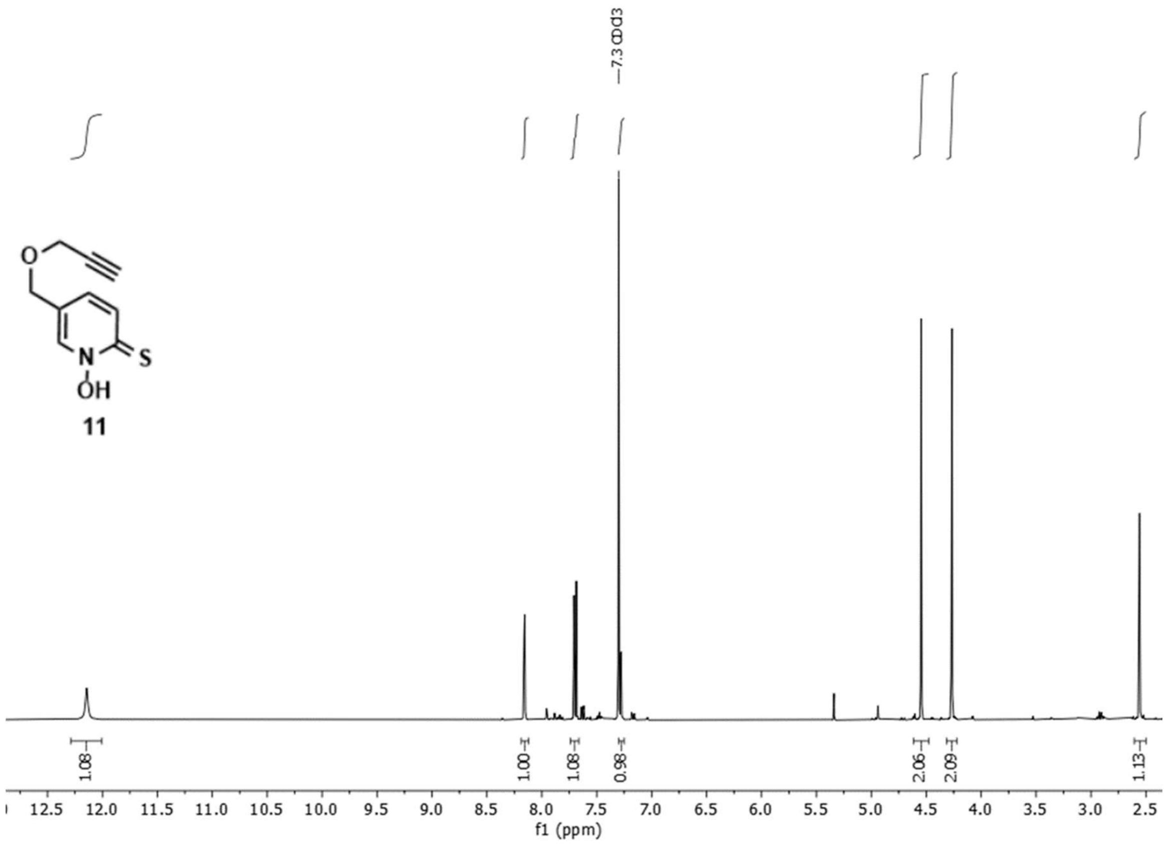

Figure S 60: *^{1}H NMR spectrum of compound **11** in chloroform-d (599 MHz) at 295 K*
